
JOURNAL INFORMATION
==============================
NLM Title Abbreviation: J Child Orthop
Journal ID: J Child Orthop
NLM Journal ID: 101313582

Journal of Children's Orthopaedics

ISSN: 1863-2521
EISSN: 1863-2548
Publisher: SAGE Publications

ARTICLE INFORMATION
==============================
PMCID: PMC4353816
PMID: 25754189
DOI: 10.1007/s11832-015-0637-0
Article ID: 637
Article version: 1
Subjects: Abstracts

EPOS 34th Annual Meeting

Electronic publication date: 2015 Mar 10
Print publication date: 2015 Apr
Volume: 9
Issue: Suppl 1
First page: 11
Last page: 56
Copyright: © The Author(s) 2015
License: This article is distributed under the terms of the Creative Commons Attribution License which permits any use, distribution, and reproduction in any medium, provided the original author(s) and the source are credited.
License URL: https://creativecommons.org/licenses/by/4.0/

issue-copyright-statement: © The Author(s) 2015

==============================

**April 16**
Scientific session: Club-foot
08:10–09:00
AUDITORIUM
**OP1**/08:10–08:16

## How important is brace compliance in ponseti-treated idiopathic clubfeet?

B. Stephens Richards, MD[1], Shawne Faulks, MSR, RN, CNS,[1], Donald Virostek, CPO[1], Kevin Felton, CPO[2], Karl Rathjen, MD[1], Lori Karol, MD[1]

[1]Texas Scottish Rite Hospital, Dallas, Texas, United States
[2]Texas Scottish Hospital, Dallas, Texas, United States

**LEVEL-II**

*Purpose:* For Club feet treated by the Ponseti technique, strict adherence to bracing is considered essential for successful outcomes. The purpose of this paper is to determine treatment outcomes relative to the amount of time bracing was actually utilized.
*Methods:* Infants with idiopathic clubfeet were braced with foot abduction orthoses and a temperature data logger imbedded in a shoe. Parents were not told that compliance was being monitored. The orthoses were prescribed 22 h per day for the first 3 months followed by 12 h per night until 2 years of age. Compliance was monitored for time intervals: (1) 0–3 months, (2) 4–6 months, (3) 7–12 months, and (4) 13–18 months. Parents rarely returned with shoes to allow assessment of the 19–24 month interval. Compliance was defined as wear >80 % of prescribed time. The outcomes were assessed as *good* (plantigrade foot ± TAL only), *fair* (limited procedure), or *poor* (full PMR).
*Results:* 53 patients with 78 clubfeet averaged 2.5 years follow-up (range 1.8–4.3 years). All feet had a Dimeglio score 0 or 1 when bracing was initiated. 36 % of patients were compliant with the bracing protocol the entire time while 64 % were found to be noncompliant during one or more of the time intervals. Compliance decreased over time as seen in the intervals: (1) 83 %, (2) 78 %, (3) 64 %, and (4) 50 %. 75 of the 78 feet (96 %) were rated *good* when bracing was discontinued. However, during these 2 years of bracing, 9 feet (seven patients) relapsed. Of these 9 feet, 6 feet (5 patients) were successfully treated with recasting and TAL. 4 of 6 feet were in three compliant patients when relapse occurred while two were in noncompliant patients. The remaining 3 of the 9 relapsed feet required surgery. 2 feet in a noncompliant patient had posterior releases and 1 noncompliant patient had PMR.Two more feet in brace-compliant patients, which were rated good when bracing was completed, required surgery at ages 3 and 4 years.
*Conclusion:* No clear patterns were established between objectively-measured brace compliance and outcome. Compliance decreases over time. Most patients will wear the brace <80 % recommended time during some periods of the bracing protocol. Nevertheless, achieving a *good* outcome can be expected. Some will require recasting for relapses whether or not they are compliant with brace use.
*Significance:* Only one-third of patients will remain fully compliant with brace wear following Ponseti cast treatment. Despite this, most will complete their time of bracing with good outcomes.

**April 16**
Scientific session: Club-foot
08:10–09:00
AUDITORIUM
**OP2**/08:16–08:22

## Ponseti protocol for relapse after posteromedial release (Pmr). Is it an optimal and durable option for this challenging complication?

Ana Maria Ey Batlle[1], Imma Vilalta Vidal[1], Melisa Stitzman[1], Norberto Ventura Gómez[2]

[1]Hospital Sant Joan de Deu, Esplugues (Barcelona), Spain
[2]Hospital Sant Joan de Déu, Esplugues(Barcelona), Spain

**LEVEL-III**

*Introduction:* The application of Ponseti Protocol in PMR relapses was presented for the first time in Sorrento EPOS 2007, and published in CORR in 2009 as a multicentric study. The efficacy of this protocol was proved, but the perdurability of the results at midterm follow-up wasńt.
*Aim:* To evaluate the outcome and follow-up of this treatment for PMR relapses.
*Materials and methods:* All patients have been treated with the Ponseti Protocol for relapse of previous treatment. We excluded patients younger than 3 year, neurological feet, arthrogrypotic and just Posterior release.
*Protocol:* Cast to 40° abduction +anterior tibial tendon transfer(ATTT) with titanium anchor ± Achilles tenotomy ± plantar fasciotomy.
*Results:* A total of 48 patients with 71 feet were included, 29 male, 19 female. Age 3.2–15 year (average 7.61). The number of previous surgeries: 56 feet had 1 PMR, 12 feet 2 PMR, 3 feet 3 PMR, 7 feet had other added surgeries. All were proposed for another PMR or bone surgery. Previous casting: None in 15 feet, 1 cast in 33 feet 2 casts in 16 feet, 3 in 5 feet, 4 in 2 feet had associated surgeries to ATTT: 70 feet Achilles tenotomy, 67 feet fasciotomy, 2 feet Jones procedure, 1 foot calcaneal osteotomy, 1 foot first metatarsal osteotomy, 4 feet replacement of previous hemi-transfer.
Complications: 4 superficial infections Re-relapses: 1 foot due to the loosening of the anchor (1.4 %) 10 feet needed repeated Achilles tenotomy because of equinus relapse (14 %).
Functional results at last follow-up: dorsiflexion before treatment: average −13° (range −35 to +20)
Post-transfer: average 11.5° (range −15 to +30)
Pain: 2 feet in the same patient had morning pain.
Activities: 5 feet daily activity limitations, 51 feet practice sports Laaveg Ponseti Score average 78 (88 max–56 min) (average in 2009 : 72)
*Conclusions:* The Ponseti Protocol is effective in PMR relapses. The results are manteined at mid-term follow-up. There is a risk of equinus relapse that needs re-tenotomy (14 %).

**April 16**
Scientific session: Club-foot
08:10–09:00
AUDITORIUM
**OP3**/08:22–08:28

## Measuring consistency of gait performance over 3 speeds with ponseti treated clubfoot patients

Deborah Eastwood[1], Lucy Alderson[1], Jenny Kemp[1]

[1]Great Ormond St Hospital, London, United Kingdom

**LEVEL-III**

*Aim:* The Ponseti management of clubfoot is the best approach for maximising foot function. A recent study produced gender specific gait centile charts. Age matched comparison of typically developing (TD) and Ponseti patients preferred speed data showed no statistically significant difference for velocity, cadence of step length (SL). Gait abnormalities observed during the fast and slow passes suggested speed modulation was difficult for some participants. This study assesses the ability of Ponseti patients to maintain consistent gait centiles at different speeds relative to their peers.
*Methods:* 20 Ponseti patients were studied: range of movement (ROM), muscle strength and balance were assessed before completing 4 passes along the GAITRite Electronic Walkway (CIR Systems Inc), at preferred, fast and slow walking speeds, and temporo-spatial data exported. LMS* growth was used to calculate Z scores and centiles for each patient at each speed. The sample was divided into consistent and inconsistent performers depending on their ability to maintain consistent centiles compared to peers across the speeds. Inconsistent performers were identified as those with a large discrepancy between centile results. Each group was examined in relation to TD peers using Z scores, before exploring their own variations in their centile results.
*Results:* Each category consisted of 10 children (7 male, 3 female). Consistent group: median age 43.5 m (range 20–65 m), 5 unilateral and 5 bilateral cases. Inconsistent group: median age 39 m (range 23–70 m) 3 unilateral and 7 bilateral cases. Muscle imbalance, weakness or reduced ROM were recorded in 3 consistent performers compared to 6 inconsistent performers. Z score analyis (one-tailed $t$ test) revealed no significant difference at any speed for consistent performers and significant results for inconsistent performers at fast speed (velocity $p = 0.037$; cadence $p = 0.003$). Examining groups separately using the preferred centile as a control revealed no statistically significant difference between fast/slow speeds with consistent performers. Inconsistent performers exhibited statistically significant differences between preferred and fast centiles (velocity $p = 0.017$; cadence $p = 0.005$).
*Conclusion:* The Ponseti method corrects the deformity; however, residual abnormalities continue to affect the ability of some patients to generate higher walking velocities. Children rarely walk at one speed. We have identified a proportion of Ponseti patients who experience difficulty with velocity modulation.
*Significance:* Gait velocity is dependent on cadence and SL. Inability to modulate one component affects overall gait performance. GAITRite and centile assessment reinforces clinical observations with meaningful performance evaluation and supports clinical reasoning.
** L = skewness. M = median S = coefficient of variation*

**April 16**
Scientific session: Club-foot
08:10–09:00
AUDITORIUM
**OP4**/08:36–08:42

## Lateral tibiocalcaneal angle as a determinant for percutaneous achilles tenotomy in the patients with idiopathic congenital clubfeet

Seungcheol Kang[1], Soo-Sung Park[1]

[1]Asan Medical Center, Seoul, Republic of Korea

**LEVEL-III**

*Introduction:* The aim of the present study is to evaluate the clinical value of the lateral tibio-calcaneal (LTiC) angle on a maximum ankle-dorsiflexion lateral radiograph, instead of ankle dorsiflexion (ADF) angle on physical examination, as a determinant indicator for percutaneous Achilles tenotomy (PAT) in the patients with idiopathic congenital clubfoot.
*Methods:* We reviewed 125 idiopathic congenital clubfeet treated at our institute since 2006 with the exclusion of clubfeet with Dimeglio grade I or with a follow-up duration less than 2 years. The clubfeet were grouped according to the LTiC angle (≤80° vs. >80°) and ADF angle (≥15° vs. <15°). We sought to present the clinical outcomes of each group and investigated the prognostic effects of each angle-based decision.
*Results:* The patients with favorable LTiC angle (≤80°) showed no sagittal relapse although none of them received PAT. Whereas, the patients with unfavorable LTiC angle (>80°) had high chance of sagittal relapse if PAT had not been performed, although they had favorable ADF angle (≥15°). For the relapse-free survival and surgery-free survival, the adherence to LTiC angle-based decision showed most significant prognostic values in multivariate analyses, but the adherence to ADF angle-based decision did not.
*Conclusion:* This is the first study reporting the objective determinant which can indicate the performance of Achilles tenotomy more accurately in clubfoot patients. The LTiC angle on a plain radiograph is believed to be more objective and prognostic indicator for Achilles tenotomy than the ADF angle on physical examination.

**April 16**
Scientific session: Club-foot
08:10–09:00
AUDITORIUM
**OP5**/08:42–08:48

## Clubfoot patients thought to be idiopathic that aren't: how do they do?*

B. Stephens Richards, MD[1], Shawne Faulks, MSR, RN, CNS[1]

[1]Texas Scottish Rite Hospital, Dallas, Texas, United States

**LEVEL-II**

*Purpose:* Over time, 7 % of infants thought to be normal with idiopathic clubfeet when treatment begins are found to have another diagnosis. The purpose is to compare the clinical outcomes of these "non-idiopathic" patients with idiopathic clubfoot patients.
*Methods:* Infants with clubfeet who were thought to be normal (idiopathic) at presentation, <3 months old, had no prior treatment, and had 2 year follow-up were studied. Treatment consisted of either the Ponseti method or the French physical therapy method. 591 patients with 889 clubfeet were identified. Over time, 39 of these patients with 64 clubfeet were identified as nonidiopathic due to neurological or syndromic abnormalities. The remaining 552 idiopathic patients with 825 clubfeet (Group 1) were compared to the 39 non-idiopathic patients (Group 2). The pretreatment Dimeglio severity scores were recorded for each foot. The outcomes at 2 years were assessed as *good* (plantigrade foot ± TAL only), *fair* (limited procedures), or *poor* (full PMR).
**Clinical results at 2 years**

Group 1 Good: 663 feet (80 %)—Avg score 12.7 Fair: 89 feet (9 %)—Avg score 14.4 Poor: 73 feet (11 %)—Avg score 16
Group 2 Good: 49 feet (77 %)—Avg score 13.2 Fair: 5 feet (8 %)—Avg score 15.6 Poor: 10 feet (15 %)—Avg score 13.6

The 49 non-idiopathic feet rated *Good* at 2 years were then reviewed with a minimum 3 years follow-up to determine whether further intervention was required (range 3–11.8 years, avg 7.5). 29/49 non-idiopathic feet (59 %) remained *good*. 14/49 feet (29 %) required limited procedures to maintain plantigrade feet. 6/49 feet (12 %) required PMRs.
*Conclusions:* At 2 years follow-up, patients who eventually were determined to have non-idiopathic clubfeet had similar outcomes to those with idiopathic clubfeet. Beyond that time, approximately 40 % of those non-idiopathic clubfeet that had *good* 2-year outcomes will need further intervention. This percentage exceeds that of idiopathic clubfeet with *good* 2-year outcomes needing further intervention (27 % reported at POSNA 2014).
*Significance:* Those infants with clubfeet who are initially thought to be idiopathic, but later are found to have a neurological or syndromic abnormality, can be expected to respond to nonoperative treatment similar to the idiopathic population at the 2-year follow-up. However, with extended follow-up, these patients will require more surgical intervention than those with idiopathic clubfeet.

**April 16**
Scientific session: Club-foot
08:10–09:00
AUDITORIUM
**OP6**/08:48–08:54

## Anterior distal tibial guided growth using eight-plates for the treatment of residual equinus deformity in children with clubfoot

Konstantinos Tsitskaris[1], Tim M Parratt[1], Daniel Williams[1], Christopher Bradish[1], Deborah M Eastwood[1]

[1]Great Ormond Street Hospital, London, United Kingdom

**LEVEL-IV**

*Aim:* The treatment of residual equinus deformity in children with clubfoot can be very challenging. When the non-operative management fails, the surgical treatment can extend from an Achilles tenotomy or lengthening to procedures as invasive as triple arthrodesis or gradual correction using the Ilizarov fixator. We evaluate the effect of guided growth using anterior distal tibial eight-plates in the treatment of residual equinus deformity in children with clubfoot.
*Methods:* We evaluated 5 children (6 feet) with residual equinus deformity after surgical treatment of clubfoot in our institution from 2010 to 2014. Clinical and radiographic parameters were used to assess the correction of the residual equinus deformity. Measurements using a goniometer were recorded pre-operatively, then to confirm the plantigrade position of the foot and at the latest follow-up. We also used preoperative and follow-up radiographs and measured the anterior distal tibial angle (ADTA). Pain and function were assessed with 10-cm visual analogue scale (VAS) scores before the implantation of the eight-plates and at the latest follow-up. The plates were removed when the foot was clinically plantigrade.
*Results:* The mean follow-up after the removal of the plates was 14.2 months. The preoperative equinus deformity was measured to be a mean of 30° (range 20°–40°). The final amount of equinus, at the latest follow-up after removal of the eight-plates, was measured to be a mean of 5° (range 0°–10°). The ADTA was measured to be a preoperative mean of 84.3° (range 82°–92°). At the time of removal of the eight-plates, the ADTA averaged 76.5° (range 73°–84°). The preoperative VAS score for pain was reported to be a mean of 6.75 (range 6–8), improving to a mean of 2.25 (range 1–4) at the last follow-up. The pre-operative VAS score for function was a mean of 3.5 (range 3–5), improving to a mean of 8.25 (range 8–9) by the last follow-up. All these changes were statistically significant.
*Conclusions:* Guided growth of the anterior distal tibia is an effective treatment for residual equinus in children with clubfeet. Follow-up to assess for recurrence of equinus is advised. If it occurs the process could be potentially repeated.

**April 16**
Scientific session: Foot & Ankle + Cerebral palsy09:02 - 10:00
AUDITORIUM
**OP75** / 09:02 - 09:08

### Treatment of Calcaneal Apophysitis: a Pragmatic Therapeutic Randomized Clinical Trial*

Johannes Wiegerinck[1], Peter AA Struijs[2]

[1]Academic Medical Center, Amsterdam, Netherlands [2]AMC Amsterdam, Amsterdam, Netherlands

**LEVEL-I**

**Introduction:** Calcaneal apophysitis is a frequent cause of heel pain in children and is known to have a significant negative effect on the quality of life in affected children. The most effective treatment is currently unknown. The purpose of this study is to evaluate three frequently used conventional treatment modalities for calcaneal apophysitis.
**Methods:** Three treatment modalities were evaluated and compared in a prospective randomized single blind setting: a pragmatic "wait and see" protocol versus a heel raise inlay (ViscoHeel, Bauerfeind, Thüringen, Germany) versus an eccentric exercise regime under physiotherapeutic supervision. Treatment duration was ten weeks. Inclusion criteria: age between eight and fifteen years old, at least four weeks of heel pain due to calcaneal apophysitis, with a minimal VAS-pain of three points. Primary exclusion criteria included other causes of heel pain and previous similar treatment. Primary outcome was VAS-pain at three months. Secondary outcomes: patient satisfaction and Oxford Ankle Foot Questionaire(OAFQ). Points of measure were at baseline, six weeks and three months. Analysis was performed according to the intention-to-treat principles.
**Results:** 101 subjects were included. Three subjects were lost to follow-up. At six weeks: the heel raise subjects were more satisfied compared to both other groups ($p < 0.01$). The heel raise group improved significantly compared to the "wait and see" group for OAFQ Children ($p < 0.01$); the physical therapy group showed significant improvement compared to the "wait and see" group for OAFQ Parents ($p < 0.01$). Each treatment modality showed significant improvement of all outcome measures during follow-up ($p < 0.005$). No clinical relevant differences were found between the respective treatment modalities at final follow-up.
**Conclusion:** Treatment with "wait and see", a heel raise inlay or physical therapy each result in a clinical relevant and statistical significant reduction of heel pain due to calcaneal apophysitis. No significant difference in heel pain reduction was found between individual treatment regimes. Calcaneal apophysitis is effectively treated by the evaluated regimes. Physicians should deliberate with patients and parents regarding the preferred treatment.

**April 16**
Scientific session: Foot & Ankle + Cerebral palsy
09:02 - 10:00
AUDITORIUM
**OP8** / 09:08 - 09:14

### Arthroscopic Talocalcaneal Coalition Resection in Children

Jorge Knorr[1], Francisco Soldado[1], Paula Diaz-Gallardo[1], Jerome Sales de Gauzy[2]

[1]Hospital Sant Joan de Déu, Barcelona, Spain [2]Hopital des Enfants Toulouse, Toulouse, France

**LEVEL-II**

***Purpose:***To describe an arthroscopic technique for talocalcaneal coalitions (TCC) resection in children and to report the outcomes.
***Methods:*** *A* prospective study in 17 consecutive persistent symptomatic feet with TCC in 16 children. The mean age was 11.8 years and mean follow-up was 28 months. A posterior arthroscopic TCC resection was performed. Pre- and post-operatively plantar footprint, subtalar motion, pain, AOFAS Ankle-Hindfoot scale were evaluated. Preoperative CT scans were used to classify the coalition, measure the percentage of involvement of the surface area and to determine the degree of hindfoot valgus. Postoperative CT scans at 1 and 3 years were used to assess recurrences. Patient satisfaction was also evaluated.
***Results:*** In all cases, the arthroscopic approach enabled complete resection of the TCC and the visualization of the posterior talocalcaneal joint. All patients increased at least one stage in the footprint and showed clinical subtalar mobility after surgery. All patients showed a statistically significant improvement in pain after surgery except for one patient developing a complex regional pain syndrome (CRPS) ($p < 0.001$). Mean preoperative AOFAS score was 56.8 while it was 90.9 postoperatively revealing a statistically significant increase ($p < 0.001$). Preoperative CT scans showed that all TCCs involved the medial subtalar joint facets with a mean involvement of 40,8 % of the articular surface. All postoperative CT scans revealed complete synostosis resection with no recurrences at final follow-up. Except for the patient who developed CRPS, all patients were either satisfied or extremely satisfied with the procedure.
***Conclusion:*** Although the arthroscopic TCC resection is a demanding technique, it allowed for precise coalition resection, an easy visualization of the posterior talocalcaneal joint and a less invasive approach that might ultimately lead to faster recovery and better outcomes.

**April 16**
Scientific session: Foot & Ankle + Cerebral palsy
09:02 - 10:00
AUDITORIUM
**OP9** / 09:14 - 09:20

### Resection of Navicular Bone as an Option in the Operative Treatment of Severe and Contracted Neurogenic Equinovalgus Feet

Chakravarthy U Dussa[1], Leonhard Doederlein[1]

[1]Orthopaedische Kinderklinik, Aschau, Germany

**LEVEL-II**

**Introdution:** Severe and contracted equino valgus feet (EVF) are often seen in severe cases of cerebral palsy. They usually cause either pain during standing or wearing orthosis or conflict with foot wear.
**Methods and Materials:** In a prospective study, 30 consecutive patients (48 feet) who underwent naviculectomy as a procedure in correction of EVF were included. Patients with follow-up under 1 year were excluded from the study. The commonest etiology of

EVF is cerebral palsy and sometimes psychomotoric retardation. During the surgical procedure, Navicular bone was removed to adjust for the discripany in length between the long medial column and the short lateral column. Then a Talo-Cuneaform & Calcaneo-cuboid arthrodesis was performed. Several other procedures such as lengthening of tendo achillis, great toe surgery for valgus deformity, supramalleolar torsion correction, calcaneal shift were performed. All the patients were immobilised in a plaster cast for 6 weeks and subsequently mobilised using a temporary orthosis.

**Results:** Average follow-up was 3,8 years. One patient (2 feet) was lost for follow-up. . Primary or interval wound closure was achieved in all cases. Plantigrade foot position was achieved in all cases. Complications included: non-union – 1, revision surgery – 1, skin necrosis – 0, residual equinus – 1, infection – 0. 46 feet were assessed based on clinical assessments and radiological measurements. The results were classified as: poor – 2 feet, fair – 6 feet, good – 18 feet, and excellent – 20 feet.

**Discussion:** Usually severe and rigid EVF are treated either by Chopart or Triple arthrodesis. Literature on Naviculectomy as a procedure in the treatment of severe and rigid EVF is very limited. In our experience naviculectomy offers an excellent oportunity for correction. It also has several advantages in treating these problems. Resected navicular bone is an excellent source of bone graft. Moreover a additional deformity in the naviculo-cuneaform joint can be simultaneously corrected. In these cases, the skin on the lateral side of the foot is contracted due to longstanding forefoot abduction. Correction of this deformity by additive arthrodesis leads to wound closure problems. The only side-effect of naviculectomy is foot shortening which is about 2 cm which does not cause any functional disability or cosmetic abnormality.

**Conclusion:** Naviculectomy allows excellent correction of a severe and contracted EVF in all three dimentions. There is no loss of correction at 3.8 years of follow-up

**April 16**
Scientific session: Foot and ankle + cerebral palsy
09:02–10:00
AUDITORIUM
**OP10**/09:20–09:26

## Foot and ankle function at maturity after ilizarov treatment for atrophic-type congenital pseudarthrosis of the tibia (CPT). A comprehensive outcome comparison with the norms

Sang Gyo Seo[1], In Ho Choi[2], Yeon Soo Kim[2], Dong Yeon Lee[2], Won Joon Yoo[2], Tae-Joon Cho[2]

[1]SNU Children's Hospital, Seoul, Republic of Korea
[2]SNUCH, Seoul, Republic of Korea

**LEVEL-II**

*Purpose:* The Authors' fibula status-based Ilizarov treatment for atrophic-type CPT targets for preserving the integrity of ankle mortise with fibular stabilization allows free ankle motion after treatment. This prospective study compared the biomechanical function and outcomes of the foot and ankle at skeletal maturity in children with atrophic-type CPT treated by the Ilizarov method to healthy young adults controls with normal feet.

*Methods:* Participants were recruited from a consecutive series of 47 children who underwent Ilizarov treatment for unilateral atrophic-type CPT between 1992 and 2010. Only those who reached skeletal maturity, and who consented to participate in this IRB approved study were included. Twenty patients (mean 19.3 years, 12–24) who met the inclusion criteria were compared to 20 normal controls (mean 22.6 years, 19–25). All participants were analyzed with full 3D gait analysis of the lower limb including inter-segmental motion of the foot using a multi-segment foot models (3D FEM gait analysis), pedobarograph, physical examination, radiographic measurements, and validated outcome questionnaires (AOFAS hindfoot, Oxford). Foot and ankle biomechanical function of the patients' treated limbs were compared to that of the healthy young adults, also to that of patients' contralateral normal limbs. Non-normally distributed data and categorical data were analyzed with non-parametric test (Mann–Whitney).

*Results:* Foot gait analysis and pedobarograph showed differences in the morphology of the foot of the affected limbs, as compared to contralateral normal limbs and normal control, with (a) increased hindfoot pronation in the presence of forefoot supination; (b) decreased dorsiflexion in hallux motion; (c) increased internal rotation of the foot during terminal swing and initial stance phase as assessed by the foot progression angle; (d) decreased peak power of hindfoot plantar-flexion during push-off. However, there was no significant difference in the sagittal ROM of the forefoot and hindfoot between the affected and unaffected limbs, also between the affected limbs and normal control limbs. There were great variations in AOFAS score (mean 90.8, 78–100) and Oxford score (mean 71.9, 26.7–100) among patients.

*Conclusion:* This study demonstrates that, through its use of multiple dynamic assessments and outcome instruments, well-treated atrophic-type CPT children can have satisfactory biomechanical foot and ankle function and clinical outcomes at skeletal maturity. These findings indicate that authors' fibula status-based Ilizarov treatment for atrophic-type CPT is a viable option in light of its capability to preserve the foot and ankle function, allowing free ankle motion.

**April 16**
Scientific session: Foot and ankle + cerebral palsy
09:02–10:00
AUDITORIUM
**OP11**/09:34–09:40

## Rectus femoris transfer in cerebral palsy patients with stiff knee gait

Myung Ki Chung[1], Chin Youb Chung[1], Kyoung Min Lee[1], Sang Hyeong Lee[2], Dae Gyu Kwon[3], Ki Hyuk Sung[4], Seung Yeol Lee[4], In Ho Choi[5], Tae-Joon Cho[5], Won Joon Yoo[5], Moon Seok Park[6]

[1]SNU Bundang hospital, Seong Nam, Republic of Korea
[2]Dongguk University Hospital, Go Yang, Republic of Korea
[3]In-Ha University Hospital, In Cheon, Republic of Korea
[4]Myongji Hospital, Go Yang, Republic of Korea
[5]SNU Children's Hospital, Seoul, Republic of Korea
[6]SNU Bundang Hospital, Seong Nam, Republic of Korea

**LEVEL-IV**

*Purpose:* Although several studies have reported on the outcomes of rectus femoris transfer (RFT), few have investigated the multiple factors that could affect the results. Therefore, we evaluated the outcomes of RFT and analyzed factors that influence improvement and annual change in knee motion after surgery in patients with cerebral palsy (CP).

*Methods:* We reviewed ambulatory patients with CP who were followed up after they had undergone RFT as part of a single-event multilevel surgery (SEMLS) and who had undergone preoperative and postoperative three-dimensional (3D) gait analysis between January 1995 and December 2012. Relevant kinematic values, including peak knee flexion, knee range of motion, and timing of peak knee flexion in the swing phase and gait deviation index (GDI) score, were the outcome measures. Improvements in rate of angle and GDI score were adjusted by multiple factors such as sex, Gross Motor Function Classification System (GMFCS) level, anatomic type of CP, and concomitant surgeries as the fixed effects, and follow-up duration, laterality, and each subject as the random effects. All these were performed using a linear mixed model.

*Results:* A total of 290 patients (487 limbs) and 612 3D gait analysis results (2–4 per patient) were finally included in this study. At 2 years after RFT, estimated mean peak knee flexion (1.2°, $p = 0.005$), estimated mean knee range of motion (10.7°, $p < 0.001$), and estimated mean GDI score (7.3, $p < 0.001$) increased significantly. Peak knee flexion in the swing phase occurred 5.4 % earlier after surgery compared with that at the baseline ($p < 0.001$). In serial postoperative gait analyses, peak knee flexion in the swing phase occurred 0.8 % earlier per year in patients with GMFCS level I or II ($p = 0.021$).

*Conclusion:* RFT as part of a SEMLS was effective in treating stiff knee gait. In serial postoperative gait analyses, patients with GMFCS level I or II showed better prognosis than those with level III with regard to timing of peak knee flexion in the swing phase.

*Significance:* In the present study, peak knee flexion, knee range of motion, and timing of peak knee flexion in the swing phase and GDI score improved after RFT. During follow-up, patients with GMFCS level I or II showed better prognosis than those with GMFCS level III with regard to timing of peak knee flexion in the swing phase.

**April 16**

Scientific session: Foot and ankle + cerebral palsy

09:02–10:00

AUDITORIUM

**OP12**/09:40–09:46

## Recurrence of knee flexion deformity after hamstrings surgical lengthening: can semitendinosus transfer improve the results?

Mauro Cesar Morais Filho[1], Marcelo Fujino[1], Daniella Lins Neves[1], Francesco Blumetti[1], Catia Kawamura[1], José Augusto Lopes[1]

[1]AACD, SAO PAULO, Brazil

**LEVEL-III**

*Purpose:* The aim of this study was to evaluate if distal transfer of semitendinosus to the distal femur (TxST) is related to lower recurrence rate than simple medial hamstrings surgical lengthening.

*Methods:* Patients with diplegic spastic CP, GMFCS levels I to III, without previous surgical procedures at knee, who had undergone bilateral medial hamstrings lengthening or semitendinosus transfer to distal femur and who had complete documentation at the gait laboratory were included in this study. Thirty-nine patients matched the inclusion criteria and they were divided in 2 groups according surgical procedures at knees: Group A (22 patients), including patients who received medial hamstrings surgical lengthening as part of multilevel approach; Group B (17 patients), represented by patients who underwent orthopedic surgery including a TxST instead of semitendinosus surgical lengthening (STL). Clinical and kinematic parameters were evaluated pre-operatively and at follow-up for all groups. The primary outcome was to compare the number of patients with fixed knee flexion deformity (FKFD) in both groups before and after surgical intervention. Secondary outcomes included evaluation of mean knee flexion on physical examination and during gait.

*Results:* The two groups matched for gender (Group A—male 68.2 % and female 31.2 %/Group B—male 82.4 % and female 17.6 %), age at surgery (Group A—10.6 years and Group B—11.5 years) and follow-up time (Group A—5.9 years and Group B—7.2 years).GMFCS level III was more frequent in Group B (70.6 %) than Group A (31.8 %). FKFD before surgery was observed at 9.1 % of knees in Group A and at 50 % in Group B ($p < 0.001$). At final follow-up, 25 % of knees in Group A and 20.6 % in Group B had FKFD ($p = 0.647$). FKFD increased in Group A ($p = 0.047$) and decreased in Group B ($p = 0.011$) after treatment, and patients from Group A received more additional surgical procedures up to final follow-up than those of Group B ($p = 0.002$). Reduction of mean FKFD (from 7.3° to 4.4°, $p = 0.04$) and of knee flexion during gait stance phase (from 34.2° to 20.2°, $p < 0.001$) were observed only in Group B after surgical treatment.

*Conclusion:* In the present study, patients who received TxST show less recurrence of FKFD than those with STL.

*Significance:* The knee flexion deformity is frequent in cerebral palsy and it is related to crouch gait. Hamstrings surgical lengthening has been used frequently as an option to treat it. However, recurrence can be a problem during growth.

**April 16**

Scientific session: Foot and ankle + cerebral palsy

09:02–10:00

AUDITORIUM

**OP13**/09:46–09:52

## Recurrence after femoral derotation osteotomy in ambulatory youth with cerebral palsy

Chris Church[1], Nancy Lennon[1], Kevin Pineault[1], Tim Niiler[1], John Henley[1], Oussama Abousamra[1], Kirk Dabney[1], Freeman Miller[1]

[1]duPont Hospital for Children, Wilmington, United States

**LEVEL-III**

*Purpose:* Femoral derotation osteotomy (FDO) has been shown to be an effective short term treatment for in-toeing due to dynamic hip rotation in gait. The purpose of this study was to define persistence and recurrence of hip internal rotation (IR) following FDO in ambulatory children with CP and to evaluate factors that influence this outcome.

*Methods:* Following IRB approval, kinematic and passive range of motion (PROM) variables were retrospectively evaluated in ambulatory children with spastic CP who had a FDO to correct hip IR. Included cases had a pre-op evaluation (Vpre), a short term post-op evaluation (Vshort, 1–3 years post), and a long term post-op evaluation (Vlong, $\geq$5 years post). Persistence was defined at Vshort as hip IR > norm + 1SD and lack of improvement by 10°, recurrence was defined at Vlong as > norm +1 SD and >10° of recurrence compared to Vshort. Age at surgery, gait velocity, gross motor function, muscle spasticity, external tibial torsion (ETT), coronal plane pressure index, hip rotation in stance, and hip PROM midpoint were evaluated as predictors for dynamic and static recurrence using regression analysis.
*Results:* In 96 limbs (from 63 children) that underwent FDO, average stance hip rotation improved from 14° ± 12° (Vpre; age 9 ± 3 years) to 4° ± 12° (Vshort; age 11 ± 3 years) and relapsed to 9° ± 15° (Vlong; age 16 ± 3 years; $p < 0.05$ Vpre/Vshort/Vlong; Norm −5 ± 7°). Hip PROM midpoint improved from 23 ± 9° (Vpre) to 8 ± 11° (Vshort) and relapsed to 14° ± 13° ($p < 0.01$ Vpre/Vshort/Vlong; Norm 5° ± 6°). Tibial torsion became progressively external over time; −2° ± 17° at Vpre, −10° ± 16° at Vshort, and −15° ± 14° at Vlong ($p < 0.05$ Vpre/Vshort/Vlong; Norm −3° ± 9°). When considering individuals, internal hip rotation persisted in 41 % (kinematics) and 18 % (PROM) of limbs at Vshort. Recurrence was seen in 40 % (kinematics) and 39 % (PROM) of limbs at Vlong in children that demonstrated improvement at Vshort. Uncorrected ETT, and initial severity of hip midpoint PROM were significant factors associated with recurrence according to regression analysis.
*Conclusion/significance:* Although FDO is an accepted form of treatment, persistence and recurrence of internal hip rotation can occur in children with CP. Recurrence is predicted by ETT, and severe preoperative hip internal PROM. These predictors can be used to determine ideal children for surgery and surgical success.

**April 16**
Scientific session: Hip/ Lower extremity
10:30 - 11:30
AUDITORIUM
**OP14** / 10:30 - 10:36

## Low-dose biplanar radiography can assess femoral and tibial torsion accurately in children and adolescents

Franck Accadbled[1], Olivier Meyrignac[2], Julie Vial[3], Christiane Baunin[3], Jérôme Sales de Gauzy[1]

[1]Service d'Orthopédie, Toulouse, France [2]Service de radiologie, Toulouse, France [3]Service de Radiologie, Toulouse, France

**LEVEL-II**

**Purpose:** To evaluate in children the agreement between femoral and tibial torsion measurements obtained with low-dose biplanar radiography (LDBR) and CT scan. To measure dose reduction ratio between these two techniques both in vitro and in vivo.
**Material and Methods:** Thirty children with lower limb torsion abnormalities were included in a prospective, single center study. Biplanar radiographs and CT scans were performed for measurement of lower limb torsions. Values were compared using Bland-Altman plots. Inter and intraobserver agreements were evaluated by intraclass correlation coefficients. A comparative dosimetric study was performed using an ionization chamber in a tissue-equivalent dummy, and with thermoluminescent dosimeters in 5 patients.
**Results:** Average differences between CT scan and LDBR measurements were -0.1° ± 1.1 for femoral torsion and -0.7° ± 1.4 for tibial torsion. Interobserver agreement for LDBR measurements was very good for both femoral torsion (FT) (0.81) and tibial torsion (TT) (0.87). Intraobserver agreement was excellent for FT (0.97) and TT (0.89). Ratio between CT scan dose and LDBR dose was 22 in vitro (absorbed dose) and 32 in vivo (skin dose).
**Conclusion:** Lower limb torsion measurements obtained with LDBR are comparable to CT scan measurements in children and adolescents, with a considerably reduced radiation dose.

**April 16**
Scientific session: Hip/ Lower extremity
10:30 - 11:30
AUDITORIUM
**OP15** / 10:36 - 10:42

## Evaluation of Limb Lengths and Physeal Growth: The Use of Low Dose Biplanar Radiography (EOS) and Tantalum Bead Implantation

Matthew Garner[1], Matthew Dow[1], Elise Bixby[1], Douglas Mintz[1], Roger Widmann[1], Emily Dodwell[1]

[1]Hospital for Special Surgery, New York, United States

**LEVEL-II**

**Purpose**: CT scanogram is the gold standard for diagnosing limb length discrepancy and evaluating growth following epiphysiodesis. Assessing growth following epiphysiodesis is a challenge due to lack of naturally occurring landmarks to measure between. Tantalum beads have previously been used with traditional plain radiography and radiosterometric analysis to assess growth and alignment. Tantalum bead implantation in conjunction with CT scanogram or EOS imaging has not previously been described. The purpose of this study was 1) to assess the accuracy of EOS compared with CT scanogram for length measurement, and 2) to assess inter and intra rater reliability of length measurements on both EOS and CT scanogram.
**Methods**: Ten skeletally immature cadaveric lamb femurs were procured, and 0.8 mm tantalum beads were inserted into the cortex at various positions. CT scanogram and EOS imaging were obtained. Measurements of total bone length on the AP and lateral views, and measurements between each bead pair were recorded on AP and lateral views. Measurements were made by two orthopedic surgeons on two separate occasions. Repeat measures were made two weeks apart. EOS was compared to CT scanogram using pairwise Pearson correlations. Intra- and inter-rater reliability was assessed using pairwise Pearson correlations. All analyses were performed using STATA 12.0.
**Results**: EOS measurements showed near perfect correlation to those of CT scanogram ($r > 0.96$, $p < 0.001$). Intrarater reliability was excellent for all measurements with EOS ($r > 0.98$, $p < 0.001$) and CT scanogram ($r > 0.99$, $p < 0.001$) as was interrater reliability for EOS ($r > 0.98$, $p < 0.001$) and CT scanogram ($r > 0$ .99, $p < 0.001$).
**Conclusion**: EOS is comparable to CT scanogram in the assessment of total limb length and intra-bead measurements. Intra and inter rater reliability was excellent for all measurements. In many instances there was perfect correlation between observers for intra-bead measurements, and perfect correlation between repeat measures for intra-bead measurements. The benefits of EOS compared to CT scanogram may include the ability to assess both the AP and lateral planes, evaluation the legs in a weight-bearing position, and lower radiation exposure.
**Significance:** The combination of EOS imaging and tantalum bead implantation allows for accurate and reliable measurement of both overall limb growth and distances between surgeon implanted markers.

**April 16**
Scientific session: Hip/ Lower extremity
10:30 - 11:30
AUDITORIUM
**OP16** / 10:42 - 10:48

## Surgical Outcomes of Valgus Extension Derotation Osteotomy (VEDO) of the Hip in Children with Spondyloepiphyseal Dysplasia Congenita: Midterm Results

Ilhan A Bayhan[1], Tristan Nishnianidze[1], Kenneth J Rogers[1], Oussama Aboussamra[1], Micheal B Bober[1], Freeman Miller[1], William G Mackenzie[1]

[1]NAIDHC, Wilmington DE, United States

**LEVEL-III**

**Purpose:** The aim of this study is to evaluate the radiological and gait analysis outcomes of the surgical treatment of severe coxa vara in spondyloepiphyseal dysplasia congenita (SEDC).
**Methods:** Patients were identified who had SEDC, valgus extension derotation osteotomy (VEDO), and gait studies. Clinical data included the age at surgery, sex, weight, height, PODCI score, gait kinematics and hip range of motion. Neck-shaft angle (NSA) and Hilgenreiner-trochanteric (H-T) physis angle were measured pre-op, first post-op, and last visit. Concurrent surgeries, complications and secondary surgeries were noted.
**Results:** 84 children had realignment osteotomies but only, 19 hips of 10 patients had pre and postoperative gait analysis and radiograph evaluation . Average age at surgery was 9.3 ± 2 years, height was 88.5 ± 13 cm (z = -7.8) and weight was 17.7 ± 7.6 kg (z = -2.2). PODCI global functioning score for 4 patients stayed at an average score of 68 between pre-op and post-op time periods. Femoral head ossification before surgery was complete (2, 10.5%), partial (4, 21%) and none (13, 69%). Four children that had no ossification developed partial ossification after the surgery. Average time for radiological follow up was 4.5 ± 2 years and 2.6 ± 2 years for the gait analysis. Hip flexion contracture decreased from a median of $20^0$ to $0^0$ and maximum hip abduction improved from a median of $15^0$ to $30^0$ (p = 0.001). Preoperative median of $100^0$ NSA improved to median of $134^0$ postoperatively and at the final follow-up it was median of $126^0$. Preoperative NSA was significantly different than postoperative and final follow-up ($p < 0.001$) but there was no difference between postoperative and follow-up NSA. The preoperative median of $-6^0$ H-T angle improved to median of $18^0$ and was $6^0$ at the final follow-up with a significant difference across the groups ($p < 0.001$). Preoperative to postoperative change was significant ($p < 0.001$) but the difference between preoperative to follow-up was not significant. During the follow-up no statistical significant change was observed except the pelvic tilt increased. Two hips had revision surgery one for hip instability and one for unilateral recurrence. No complications occurred.
**Conclusion:** The outcome at 5 years demonstrated that in the severe SEDC group, VEDO surgery is an effective procedure to realign the proximal femur and preserve lower extremity function. Femoral head ossification showed no significant improvement after surgery.
**Significance:** Valgus osteotomy is effective in the SEDC population.

**April 16**
Scientific session: Hip/ Lower extremity
10:30 - 11:30
AUDITORIUM
**OP17** / 10:56 - 11:02

## Histopathology of human Slipped Capital Femoral Epiphysis physes*

Melinda Witbreuk[1]

[1]VUmc, Amsterdam, Netherlands

**LEVEL-II**

**Introduction:** SCFE has several known risk factors and diagnostic features but little is known about the histopathology of this entity. We conducted an observational, informed consent, study where we compared biopsies taken during the epiphysiodesis of the physis in SCFE with normal physes.
**Methods :** 20 patients with SCFE, of which 2 bilateral and 11 controls (two same patients, but different physis) were biopsied and were studied with histomorphology and immunohistochemistry. S100 expression was utilized for representativeness of the physis in the biopsies.
**Results :** The main histomorphological feature was architectural disorder of chondrocytes in physes of patients with SCFE. S100 expression of hypertrophic chondrocytes in slipped physes was maintained, with similar pattern as in control chondrocytes. Vascular proliferation was seen in only a small number of cases, suggesting that this clinical feature may sometimes be a late manifestation of slippage. We found no evidence of increased apoptosis, based on Caspase expression. Expression of eight hormonal receptors, important in puberty, were unaltered in SCFE chondrocytes compared to control chondrocytes.
**Conclusion:** SCFE generally leads to disturbed architecture of regular aligned chondrocyte in the physis, but phenotype of hypertrophic chondrocytes remains normal. This supports a biomechanical cause for slippage of the hypertrophic zone, although transient hormonal imbalance pre-slippage due to puberty without effects on expression of receptor could still play a role.
**Keywords :**SCFE, biopsy, histomorphology, immunohistochemistry

**April 16**
Scientific session: Hip/ Lower extremity
10:30 - 11:30
AUDITORIUM
**OP18** / 11:02 - 11:08

## Pelvic Incidence and Acetabular Version in Slipped Capital Femoral Epiphysis

Jeremy Gebhart[1], Michael Bohl[2], Douglas Weinberg[1], Daniel Cooperman[3], Raymond Liu[1]

[1]Rainbow Babies & Children's, Cleveland, United States [2]Brown University, Providence, United States [3]Yale University, New Haven, United States

**LEVEL-III**

**Introduction:** The aetiology of slipped capital femoral epiphysis (SCFE) is multifactorial, but the role of sagittal balance of the pelvis as a contributing factor to its development has not been well studied. Our primary purpose was to determine if a smaller pelvic incidence (PI), a position-independent anatomic parameter that regulates pelvic orientation and is defined as the angle between the line perpendicular to the sacral plate at its midpoint and the line connecting this point to the axis of the femoral heads, could be a factor that increases shear stress in the epiphyseal growth plate and potentially contributes to the development of SCFE. We also set out to determine if acetabular retroversion was associated with SCFE.

**Methods:** We obtained fourteen cadaveric pelvi from the Hamann-Todd Osteological Collection whose femurs showed evidence of post-SCFE deformity. Two-hundred age-, sex-, and race-matched pelvi in good condition and without evidence of anatomic abnormality were used as controls. Pelvic incidence and acetabular version were measured using standardized lateral photographs and a goniometer, respectively. T-tests were performed to evaluate for differences in measured parameters between groups.

**Results:** The mean PI was 40.6° ± 6.1° for SCFE specimens and 47.4° ± 9.9° for normal specimens ($p = 0.01$). The mean version of SCFE and normal acetabula was 15.2° ± 6.9° and 16.6° ± 5.9°, respectively ($p = 0.39$). There was also no significant difference in version between SCFE acetabula and the contralateral, uninvolved acetabular of the same specimen (15.2° ± 6.9° versus 16.8° ± 7.5°, $p = 0.33$). Interobserver and intraobserver ICC was 0.96 or greater for all measurements.

**Conclusion:** Specimens with SCFE deformity demonstrated a smaller PI than a large cohort of normal control specimens. We found no significant difference between acetabular version of specimens with and without SCFE deformity. Contralateral or unaffected acetabuli of SCFE specimens were not more retroverted than the affected side of the same specimen. Sagittal balance of the pelvis, and particularly decreased PI, may play an important role in the development of SCFE. The influence of mechanical factors beyond the hip joint in the development of SCFE should be considered by clinicians.

**April 16**
Scientific session: Hip/lower extremity
10:30–11:30
AUDITORIUM
**OP19**/11:08–11:14

## Continued delay in diagnosis of slipped capital femoral epiphysis

Alexander Broom[1], Lindsay Andras[1], Kody Barrett[1], Rachel Goldstein[1], Herman Luther[2], Nicholas Fletcher[3], Robert Runner[3], Christine Bowman[3], David Skaggs[1]

[1]Childrens Hospital Los Angeles, Los Angeles, United States
[2]Boston Children's Hospital, Boston, United States
[3]Childrens Healthcare Atlanta, Atlanta, United States

**LEVEL-IV**

*Purpose*: More than a decade ago, both Skaggs and Kocher et.al. reported significant delays in the diagnosis of slipped capital femoral epiphysis (SCFE). The purpose of this study was to identify if the time to diagnosis has improved.

*Methods*: A retrospective review was performed of patients admitted with a diagnosis of SCFE at three large pediatric hospitals between January 2003 and December 2012.

*Results*: 478 patients with an average age of 12 years (range 5–18 years) at presentation met the inclusion criteria. Over the entire study period, the average time from symptom onset to diagnosis was 16 weeks (range 0–169), and did not differ significantly between sites ($p = 0.3549$). Separated into 2 year intervals there were the following delays in diagnosis: 2003–2004 = 11.4 weeks; 2005–2006 = 16.4 weeks; 2007–2008 = 18.4 weeks; 2009–2010 = 13.9 weeks; 2011–2012 = 19.8 weeks ($p = 0.1312$). Average BMI was 28.7 (range 14.1–44.9). 357 patients (74 %) presented to a primary care clinic or the emergency room initially. Of those 357, 158 (44 %) had documentation of date of initial evaluation. The average delay from initial evaluation by primary care or the ER to diagnosis was 5.2 weeks (range 0–52 weeks). Severity of the Southwick angle ($p \leq 0.0001$) and grade of slip ($p = 0.0002$) correlated with time from symptom onset to diagnosis. At the time of presentation, 15 % (70/475) had bilateral slips. There was no significant correlation between insurance status and delay in diagnosis. 56 patients (11 %) developed a second SCFE after treatment of the first SCFE. There was an average of 10 weeks between onset of symptoms and diagnosis for the second SCFE, significantly less than the overall mean for the initial SCFE ($p < 0.0001$). 87 % presented for their second SCFE while they were still mild as defined by the Wilson classification.

*Conclusion:* Despite reports documenting a lag in the diagnosis of SCFE over a decade ago, there has not been any improvement in the delay in diagnosis of SCFE. In fact, delay in diagnosis trends toward worsening over the decade of this study and these delays were similar at all three geographically distinct locations across the US Slip severity and slip grade increased with time to diagnosis, so a delay in diagnosis may worsen outcomes.

*Significance:* Decreased time to diagnosis in patients with a second SCFE suggests that patient education of at risk children may be a more effective strategy to improving this delay.

**April 16**
Scientific session: Hip/lower extremity
10:30–11:30
AUDITORIUM
**OP20**/11:14–11:20

## The "skinny" SCFE

Rachel Goldstein[1], Erin Dawicki[2], Alexander Broom[1], Kody Barrett[1], Nicholas Fletcher[3], Robert Runner[3], Christine Bowman[3], Lindsay Andras[1], Michael Millis[2]

[1]Children's Hospital LA, Los Angeles, United States
[2]Boston Children's Hospital, Boston, United States
[3]Children's Hospital of Atlanta, Atlanta, United States

**LEVEL-III**

*Purpose*: Much has been made of the link between obesity and slipped capital femoral epiphysis (SCFE). However, little data exists in the literature on patients with SCFE who are underweight/normal weight. The purpose of this study was to determine if underweight/normal weight patients differ from overweight and obese patients with SCFE.

*Methods:* This was a multi-center, retrospective review of all patients presenting for treatment of a slipped capital femoral epiphysis from January 1, 2003 to December 31, 2012. Patients were excluded if they received previous surgical treatment at an outside institution or did not have a height and weight recorded in their charts. Body mass index (BMI) was calculated and categorized for each patient.
*Results:* A total of 202 patients met inclusion criteria. Average BMI was 27.9 (range 14.1–45.0). Sixteen patients (7.9 %) were underweight, 47 (23.3 %) were normal weight, 67 (33.2 %) were overweight, and 72 (35.6 %) were obese. Demographic characteristics were similar between those patients who were underweight/normal weight and those who were overweight/obese. Underweight/normal weight patients were no more likely to have pre-existing medical comorbidities known to be associated with SCFE ($p = 0.144$). They were also no more likely to present with bilateral involvement ($p = 0.374$). Underweight/normal weight patients were significantly more likely to present with an unstable slip compared to overweight/obese patients (26 versus 14 %, $p = 0.04$). In addition, underweight/normal weight patients presented with a much shorter duration of symptoms (8.5 versus 24.1 weeks, $p < 0.001$). There was no significant difference between the two groups with regards to Southwick angles and Wilson percent displacement. Underweight/normal weight were not more likely to undergo prophylactic pinning of the contralateral hip ($p = 0.983$).
*Conclusion:* Underweight/normal weight patients are more likely to present with an unstable SCFE and tended to have a shorter duration of a symptoms when compared to their overweight/obese peers.
*Significance:* SCFE does occur in underweight and normal weight patients, with similar demographic characteristics and radiographic findings. However, in underweight/normal weight patients, these slips are more commonly unstable.

**April 16**
Scientific session: Trauma/lower extremity
11:30–12:00
AUDITORIUM
**OP21**/11:28–11:34

## Fractures in children with spina bifida

Stanislav Ivanov[1], Alexei Baindurashvili[1], Vladimir Kenis[2]

[1]Turner Scient. and Res. inst., Saint–Petersburg, Russian Federation
[2]Turner Scient. and Res. inst, Saint–Petersburg, Russian Federation

**LEVEL-III**

*Overview:* It is 80–90 years since effective methods of hydrocephalus shunt and closure of neural tube defects have been developed. As a result orthopedic surgeons were faced with the problem of increasing management of children with spina bifida. According to many authors, in these patients fractures of the long bones are common. Some fractures are accompanied by excessive callus and the radiological picture can be easily confused with a tumour, osteomyelitis and other bone diseases.
*The aim of the study*: The identification of factors predisposing to fractures in children with spina bifida.
*Materials and methods*: The object of the study were 286 children who were examined and treated in the Turner Scientific and Research Institute for Children's Orthopedics (Saint-Petersburg, Russia) in the period from 2006 to 2014. The Neurosegmental level of spina bifida according to the Sharrard classification was made. Motor status was evaluated by Hoffer's criteria.
*Results:* In 286 patients 81 fractures were observed. The majority of patients with fractures had thoracic and L1–L2 level of neurosegmental lesion, 24 of 33 (72 %) and in 19 of 36 (53 %) children, respectively. 62 (76 %) patients had 3 or more fractures. In 65 from 81 (80 %) patients, motor capabilities matched Hoffer's level III and IV. 28 (36 %) patients had fractures after prolonged periods of cast immobilization after correction of contractures and deformatities of the lower extremities.
25 of 81 (31 %) patients had a second within the next 6 months after the initial fracture. Hypertrophic callus was found in 31 of 81 (38 %) patients. In a retrospective analysis of unusual radiographic features of fracture bone biopsy was performed to exclude osteosarcoma, and in 5 patients the excess callus was interpreted as a manifestation of osteomyelitis.
*Conclusions:* The most frequent fractures in children with spina bifida were observed in cases of thoracic, and L1–L2 level of neurosegmental lesion and Hoffer's III and IV levels. The X-ray pattern of fractures in a child with spina bifida must be considered in diagnosis and treatment.

**April 16**
Scientific session: Trauma/lower extremity
11:30–12:00
AUDITORIUM
**OP22**/11:34–11:40

## Redefining optimal medullary canal fill in flexible intramedullary nailing of pediatric femur fractures

Anthony Riccio[1], James Shaha[2], Matthew Cage[2], Sheena Black[3], Steven Shaha[4], Robert Wimberly[1]

[1]Texas Scottish Rite Hospital, Dallas, United States
[2]Tripler Army Medical Center, Honolulu, United States
[3]UT Southwestern Medical Center, Dallas, United States
[4]University of Utah, Draper, United States

**LEVEL-III**

*Purpose*: To assess the relationship between percent canal fill and alignment at radiographic union following flexible intrameduallary nailing (FIMN) of pediatric femoral shaft fractures.
*Methods*: An IRB approved, retrospective review of a consecutive series of patients who sustained a femoral shaft fracture treated by retrograde, stainless steel FIMN was performed at a paediatric trauma center from 2005 to 2012. Medical and surgical records were reviewed and preoperative radiographs were analyzed to determine fracture pattern, fracture location, and isthmic canal diameter. Percent canal fill was calculated using the known nail diameters and the measured isthmic diameter. Radiographs at the time of bony union were reviewed to measure shortening, coronal angulation and sagittal angulation. Canal fill was analyzed to determine correlative factors and radiographic outcome with significance defined as $p < 0.05$.
*Results*: 274 children underwent retrograde FIMN with an average age of 8.3 years (range 2.2–17.1 years). Canal fill of $\geq$80 % was seen in 108 (39.4 %) patients. When compared to those with <80 % canal fill, there were no significant differences in age (8.8 vs. 7.9 years), gender (76.8 vs. 71.1 % males), or BMI (18.5 vs. 17.2 kg/m$^2$). There were significantly more leg length differences in unstable fractures in the <80 % canal fill group (49.3 vs. 29.6 %, $p < 0.01$). Radiographic outcome was no different with respect to coronal angulation (2.6° vs. 3.0°), sagittal angulation (3.0° vs. 3.1°), or shortening (2.4 vs. 4.0 mm). Canal fill of $\geq$70 % was seen in 185 (67.5 %) patients and when compared to the <70 % canal fill group, there were no differences in shortening (3.2 vs. 3.8 mm), coronal angulation (2.7° vs. 3.0°) or sagittal angulation (2.9° vs. 3.3°). Finally, 7.5 % of the population (20 patients) had less than 60 % canal fill and did not demonstrate a significant increase in shortening, coronal or sagittal angulation compared to groups with higher percentages of canal fill. No group had an increased rate of infection, implant removal, nonunion or need for reoperation.
*Discussion:* In a large series of consecutive patients treated with retrograde stainless steel FIMN there does not appear to be any correlation between the percent of canal fill and radiographic outcome. This calls into question the generally agreed principle that optimal canal fill of >80 % is necessary for a successful result.
*Significance:* Stainless steel flexible IM nails are able to maintain fracture alignment without an increase in complications at lower percentages of canal fill than previously reported as "optimal".

**April 16**
Scientific session: Trauma/lower extremity
11:30–12:00
AUDITORIUM
**OP23**/11:40–11:48

## Assessment of side-step cutting in pedatric athletes with recent ACL reconstruction compared to those with no ACL surgical history

James Lee Pace[1], Nicole Mueske[1], Tracy Zaslow[1], Mia Katzel[1], Matthew Chua[1], Tishya Wren[1]

[1]Childrens Hospital Los Angeles, Los Angeles, United States

**LEVEL-III**

*Purpose*: This study used the side-step cut to evaluate differences between paediatric athletes' anterior cruciate ligament (ACL) reconstructed limb and non-reconstructed limb compared to limbs with no lower extremity surgical history.
*Methods:* The study included 28 limbs with an ACL reconstruction within the 12 months prior to testing (operative limbs), 28 contralateral limbs (non-operative limbs) and 56 limbs with no lower extremity surgical history (control limbs; 28 individuals). Lower extremity kinematic and kinetic 3-dimensional data was recorded during the deceleration phase of a 45° cut. Outcomes were evaluated at initial foot contact and between initial foot contact and maximum knee flexion of the cutting limb. Differences between operative and non-operative limbs and control limbs were assessed using analysis of variance with Bonferroni post hoc tests.
*Results:* Operative limbs had higher peak hip adduction ($p = 0.01$) and higher average external knee valgus moments ($p = 0.02$) compared to control limbs. In terms of shock absorption, operative limbs had lower peak ground reaction forces ($p < 0.0001$) and peak ankle dorsiflexion ($p = 0.002$) compared to control limbs with lower peak external knee flexor moments ($p < 0.0001$) and less power absorption at the knee ($p = 0.05$) and ankle ($p = 0.01$). Though non-operative limbs had lower knee valgus ($p = 0.04$) at initial contact, they had higher peak knee valgus ($p = 0.04$) and higher average external knee valgus moments compared to control limbs. Non-operative limbs also had less pelvic obliquity compared to control limbs ($p = 0.04$). Lower peak ground reaction forces ($p = 0.005$), increased power absorption at the hip ($p = 0.005$), and decreased power absorption at the ankle ($p = 0.09$) were seen in the non-operative limbs compared to controls.
*Conclusions:* Limbs with ACL reconstruction exhibited poorer hip stability compared to control limbs. ACL reconstructed limbs also had less energy absorption at the knee and lower peak vertical ground reaction forces, likely reflecting an avoidance strategy when performing a cut using an ACL reconstructed limb. Contralateral limbs of ACL reconstructed patients also demonstrated reduced ground reaction forces and altered neuromuscular control. These changes may reflect overall tentativeness in performing a cut. It is also possible that the biomechanical strategies present on the operative side could be putting the non-operative limb at risk for injury.
*Significance:* Deficits in coronal plane hip and knee control in ACL reconstructed limbs during side-step cutting may contribute to a risk of re-injury, while alterations in sagittal plane shock absorption strategies and coronal plan knee control may place the contralateral limb at risk for future injury.

**April 16**
Scientific session: Basic science
14:25–15:45
AUDITORIUM
**OP24**/14:25–14:31

## Autogenus cultured growth plate chondrocytes transplantation in treatment of physeal injury in rabbits*

Ryszard Tomaszewski[1]

[1]GCZD, Katowice, Poland

**LEVEL-I**

*Abstract:* Excision of a portion of the medial aspect of the proximal tibial physis led constantly to the formation of a bone bridge, growth arrest and angular deformity.
*Objectives*: The aim of this experimental study on New Zealand's white rabbits was to investigate the transplantation of autogenous growth plate cells to treat the injured growth plate. They were assessed in terms of measurements of radiographic tibial varus and histological characteristics.
*Methods*: The 14 New Zealand white rabbits at the age of 5 weeks were selected. An experimental model of growth plate medial partial resection of tibia in 14 New Zealand white rabbits was created. A standard defect was thus created by removing a quarter of the proximal tibial physis, and standard drainage tube was put inside. During this surgical procedure the plate growth cells were collected and then they were cultured. While the second surgery (3 weeks after the first) was being performed the autologous cultured growth plate cells were grafted into the right tibia. The left tibia was used as a control group. 60 days after the second surgery, the experimental animals underwent euthanasia. The tissues were taken for histological and radiographic examination.
*Results:* Histological examinations showed that grafted right tibia presented a regular shape of the plate growth with hypertrophic maturation, chondrocyte columniation and enchondral calcification. But only on the right tibia (sample No. 14) (after grafting of the autologous physeal cartilage) presented bone bridge formation. A-P and lateral view Xrays of the tibia presented regular visible growth plate at the site of grafting at right tibia. But the growth plate in 3 samples (samples No. 7, 8, 9,) was irregular with the folded shape.Radiographic study shows that the average tibial deformity at the left angle was 20.29° and in the right was only 7.21°.
*Conclusion:* This study has demonstrated that grafting of an autogenous cultured growth plate cells into a defect of the medial aspect of the proximal tibial physis can prevent bone bridge formation, growth arrest and the development of varus deformity.

**April 16**
Scientific session: Basic science
14:25–15:45
AUDITORIUM
**OP25**/14:31–14:37

## COMP-angiopoietin1 potentiates the effects of bone morphogenic protein-2 on ischemic necrosis of the femoral head in rats*

Jung Ryul Kim[1]

[1]Chonbuk National University, Joenju, Republic of Korea

**LEVEL-I**

*Introduction:* Angiogenesis is considered essential for proper bone regeneration. The purpose of this investigation was to determine if a combined therapy of bone morphogenetic protein-2 (BMP-2) and cartilage oligomeric matrix protein angiopoietin-1 (COMP-Ang1) can potentiate the therapeutic effect of BMP-2 in a rat model of ischemic necrosis of the femoral head (INFH).
*Methods:* INFH was surgically induced in the femoral head of rats, and the animals were divided into the following groups: (1) a sham-operated group (sham group), (2) a bovine serum albumin-injected group (BSA group), (3) a BMP-2-injected group (BMP-2 group), and (4) a COMP-Ang1 and BMP-2-injected group (COMP-Ang1 + BMP-2 group) (n = 20/group). Radiological, histological, and histomorphometric assessments were performed to assess femoral head morphology, vascular density, and bone resorption activity. Western blots and immunohistochemical staining were performed to evaluate production of BMP-related signaling proteins in C3H10T1/2 cells and tissues. Real-time RT-PCR was performed to investigate expression of the target integrin gene, and the effect of integrin on C3H10T1/2 cells was determined using a cell adhesion assay.
*Results:* Radiographs obtained 6 weeks after injection revealed better preservation of the architecture of the femoral head in the COMP-Ang1 + BMP-2 group compared with the BSA and BMP-2 groups. Histological findings indicated increased trabecular bone and vascularity and decreased osteoclast bone resorption activity in the COMP-Ang1 + BMP-2 group compared with those in the BSA and BMP-2 groups. The combination of COMP-Ang1 and BMP-2 increased phosphorylation of Smad1/3/5, p38, and Akt. Increased integrin a3 and b1 mRNA expression in the COMP-Ang1 + BMP-2 group promoted cell adhesion.
*Conclusion:* These results suggest that COMP-Ang1 preserved the necrotic femoral head through the potentiation of BMP-2 signaling pathways and angiogenesis. Combination treatment with COMP-Ang1 and BMP-2 may be a clinically useful therapeutic application in INFH.

**April 16**
Scientific session: Basic science
14:25–15:45
AUDITORIUM
**OP26**/14:37–14:43

## A combination of sclerostin antibody and zoledronic acid treatment outperforms either treatment alone in a mouse model of osteogenesis imperfecta*

David Little[1], Lauren Peacock[1], Kathy Mikulec[1], Michaela Kneissel[2], Ina Kramer[2], Tegan Cheng[1], Aaron Schindeler[1], Craig Munns[1]

[1]Children's Hospital Westmead, Westmead, Australia
[2]Novartis Pharma, Basel, Switzerland

**LEVEL-I**

*Introduction:* Osteogenesis Imperfecta (OI) is a genetic disorder featuring bone fragility and decreased bone mass. Bisphosphonates in children with OI reduce bone catabolism and rely on modelling to form new bone. An anabolic treatment, Anti-Sclerostin Antibody (Anti-SOST Ab), is being investigated in clinical trials. We hypothesized that combined treatment may produce superior outcomes.
*Methods:* Female Col1a2 G610C mice and their wild type littermates (WT) were treated from week 5 to 9 of life with either saline (control), zoledronic acid (ZA) 0.025 mg/kg sc weekly, Anti-SOST Ab given 50 mg/kg IV weekly (Anti-SOST), or a combination of both (ZA Anti-SOST). Outcomes included weekly DEXA for areal Bone Mineral Density (BMD) (GE Lunar PIXImus WI, USA), μCT (SkyScan 1174 Kontich, Belgium), mechanical testing of tibiae in 4 point bending (Instron 5944, Massachusetts, USA). Data were analysed with one-way ANOVA (SPSS v11).
*Results:* Increases in tibial BMD were seen over time in all groups. Anti-SOST treatment alone had no effect on tibial BMD, while ZA (16 %) and ZA Anti-SOST (27 %) treatments produced significant increases from weeks 1 to 4 ($P < 0.05$). μCT analysis showed increases in Tissue Mineral Density and Cortical Thickness for combined treatment over respective controls. Tibial 4-point bending showed only combined ZA Anti-SOST yielded a significant increase in strength and energy to failure in OI mice, restoring bone strength to the values of untreated WT mice. In the spine, all treatments increased compression strength over control, Anti-SOST 30 %, ZA 43 % and ZA Anti-SOST 91 % ($P < 0.05$).
*Discussion:* Anti-SOST Ab alone had effects on trabecular but not cortical sites in this study in Col1a2 G610C mice. Roschger et al. reported minimal effect in the Col1a1(Jrt)$^{/+}$ mouse model treated with Anti SOST Ab, whereas large effects were noted with just 2 weeks treatment in 8 week-old Brtl$^{/+}$ mice, leading to increase in bone size and strength.
*Conclusion*: A combination of Zoledronic Acid and Anti-Sclerostin antibody is superior over either treatment alone in the Col1a2 G610C model of OI. Further studies are required in alternate mouse models of OI to confirm efficacy across different models, and thus to predict possible efficacy across the heterogeneous population of OI patients.

**April 16**
Scientific session: Basic science
14:25–15:45
AUDITORIUM
**OP27**/14:51–14:57

## Treatment of large bone defects. An animal model to assess the role and mechanisms of action of mesenchymal stem cells in bone regeneration*

Ana-Belen Gonzalez-Gil[1], Julio Duart-Clemente[2], Gloria Abizanda[3], Juan-Ramon Valentí-Nin[3], Felipe Prosper[3], Froilan Granero-Molto[3]

[1]University Clinic of Navarra, Pamplona, Spain
[2]Navarra Hospital Complex, Pamplona, Spain
[3]University Clinic of Navarre, Pamplona, Spain

**LEVEL-I**

*Background:* In the treatment of large bone defects autograft supply is limited and alternatives as allografts only provide osteoconductive properties that are prone to graft failure and the appearance of non-union. Non-union is a challenging clinical problem and a major cause of chronic pain and disability An attractive alternative to bone autografts is the use of autologous mesenchymal progenitors cells (MSCs) in combination with biomaterials. Although there are several reports in the clinical use of MSCs their fate, contribution to repair tissue and mechanisms of action during the repair process are not yet determined.
*Objectives:* To develop an animal model of fracture non-union that will allow us to determine the therapeutic potential of mesenchymal stem cells (MSCs) and their clinical use.
*Materials and methods:* Ten to twelve weeks-old female Sprague–Dawley rats were anesthetized and intubated. Using a lateral approach the right femoral shaft was exposed and stabilized with an aluminum plate (20 mm long, 4 mm wide, 2 mm thick) and four screws (1.5 mm diameter, 8 mm long). A diaphyseal critical size defect (5 mm wide) was performed using a dental bur. Two groups were created, a nonunion group (Group 1, n = 5) where the defect was left empty and a positive control (Group 2, n = 6) where the defect was filled with a live allograft derived from GFP transgenic rats. The healing process was monitored by radiography (Faxitron) at weeks 1, 5, 7 and 10. After 10 weeks animals were sacrificed and the healing status was analyzed by micro computed tomography (mCT), histology and inmunohistochemistry (IHC).
*Results:* In the Group 1 four of five of the animals did not show signs of healing during the radiological follow-up. Experimental non-union was confirmed by mCT. Histological analysis showed that the defect was invaded by fibrous tissue that hampers bone union. In the Group 2 bone bridging was radiographically determined and confirmed by mCT in all the animals. Histological analysis showed abundant bone bridging between graft and defect edges. In addition, using IHC GFP + cells were detected in the defect area suggesting a substantial contribution of the graft to the repair tissue.
*Conclusions:* We have successfully created and validated a reproducible model of a fracture non-union that will allow us to evaluate the role of mesenchymal cells and other biological therapies in bone regeneration and in the clinical treatment of fracture non-union.

**April 16**
Scientific session: Basic science
14:25–15:45
AUDITORIUM
**OP28**/14:57–15:03

## Anti-SOST antibody augments the anabolic response to bone loading*

Alyson Morse[1], Michelle McDonald[2], Aaron Schindeler[1], Michaela Kneissel[3], Ina Kramer[3], David Little[1]

[1]Childrens Hospital at Westmead, Westmead, Australia
[2]The Garvan Institute, Sydney, Australia
[3]Novartis Pharma, Basel, Switzerland

**LEVEL-I**

*Introduction:* Osteoporosis affects many children, not only with primary bone disorders such as Osteogenesis Imperfecta, but also individuals with secondary and disuse osteoporosis such as in cerebral palsy and Duchenne muscular dystrophy. Anti-resorptives like bisphosphonates are only marginally effective in situations where bone anabolism is deficient. Novel approaches to increase new bone formation include mechanical stimulation (e.g. vibration platforms) and pharmacological (e.g. Anti-SOST, an antibody targeting Sclerostin protein). The effect of combined treatment of mechanical stimulation and Anti-SOST is not known. To address this, we carried out a two-week study of tibial cyclic compressive loading in C57Bl/6 mice treated with Anti-SOST.
*Methods:* 10 week old female C57Bl/6 mice were treated weekly with 100 mg/kg Anti-SOST (Novartis Pharma) or Vehicle (N = 8). Cyclic (1,200 cycles/day) compressive axial loading of 9 N (1,200 με on mid-shaft) was performed on the left tibiae of all mice 5 days/week at a rate of 4 Hz. Contralateral tibiae behaved as non-loaded controls. Following the end-point (day 15) tibiae were analyzed by microCT (Skyscan1174, Kontich, Belgium). Analysis was performed on the mid-diaphysis and proximal metaphysis.
*Results:* An anabolic response was seen following 2 weeks Anti-SOST treatment, with significantly increased bone volume (BV) in the mid-diaphysis of the non-loaded control tibiae (7 %, $p < 0.05$). Further, an anabolic response to the compressive loading was seen within the mid-diaphysis compared to non-loaded controls, following treatment with either vehicle or Anti-SOST (BV increased, 17 % vehicle, 22 % Anti-SOST, $p < 0.05$). Interestingly, Anti-SOST treatment in combination with compressive loading resulted in a significantly greater BV compared to loading with Vehicle treatment (12 %, $p < 0.01$), suggesting an additive response to loading due to the inhibition of Sclerostin.
The increased BV seen with combined Anti-SOST treatment and loading was likely driven by a periosteal response within the mid-diaphysis, with periosteal perimeter significantly increased above loaded Vehicle tibiae ($p < 0.05$). Polar moment of inertia, a predictor of strength, was also improved in the loaded Anti-SOST tibiae above that of loaded Vehicle (16 %, $p < 0.05$). A comparable improvement in bone anabolism was seen within the proximal metaphysis of the loaded Anti-SOST tibiae versus loaded Vehicle tibiae.
*Conclusion:* Short-term Anti-SOST treatment augmented the increased anabolic bone response induced by compressive cyclic loading. This data supports a benefit for co-treatment of Anti-SOST and mechanical stimulation to improve bone mass and strength, although clinical trials are required for validation. Future work is underway to assess mechanistically the bone response to combined mechanical stimulation and Anti-SOST treatment.

April 16
Scientific session: Basic science
14:25–15:45
AUDITORIUM
**OP29**/15:03–15:09

## A newly designed hinged plate for temporary hemiepiphysiodesis an experimental study for assessing the correction of limb angular deformity and implant stress distribution in a miniature pig model*

Zhao Li[1], Zhenkai Wu[1]

[1]Xin-Hua hospital, Shanghai, China

**LEVEL-I**

*Background:* Tension-band plating technique is an attractive operative procedure to correct angular deformity at the metaphyseal level in the skeletally immature patient. However, due to the disadvantages of conventional tension-band plating, our present study aimed to investigate the efficacy and mechanical characteristics of a newly designed hinged plate in a miniature pig model.
*Methods:* We performed medial hind leg proximal tibial hemiepiphysiodesis using a tension-band plating technique, in 9.3-month-old, male Bama miniature pigs. The conventional tension band plate (CP group) or newly designed hinged plate (HP group) was randomly allocated to the left tibia and vice versa to the right tibia. After 18 weeks, angular deformity and residual stress in the plate after implant removal were compared between groups.
*Results:* Eight miniature pigs completed this protocol. There was no significantly statistical difference in the value of medial slope angle (MSA), medial proximal tibial angle (MPTA) and distal and proximal angle (DPA) at beginning and final follow-up. The corrective rate of DAP, MPTA, MSA was 2.18°/week, 0.85°/week, 0.71°/week, respectively in HP group, and 2.13°/week, 0.89°/week, 0.87°/week, respectively in CP group. The residual stress in the plate of the HP group was significant smaller than that of CP group ($P = 0.001$).
*Conclusions:* The efficacy of hemiepiphysiodesis by means of a hinged plate is similar to that of conventional tension-band under the conditions of this study. A built-in and compress-free hinge design is proposed to (1) reduce the risk of implant breakage due to improved stress distribution in the plate-screw system and (2) reduce the risk of periosteal and perichondral compressive injury.
*Significance:* The newly designed hinged plate is an effective instrument for temporary hemiepiphysiodesis and has its own advantages. It will hopefully draw the highlights into our orthopaedic practice in the future.

April 16
Scientific session: Basic science
14:25–15:45
AUDITORIUM
**OP30**/15:17–15:23

## Cam deformity is not associated with conventionally-held risk factors for femoroacetabular impingement*

William Morris[1], Roger Yuh[1], Cody Fowers[1], Katherine Xie[1], Daniel Cooperman[2], Raymond Liu[1]

[1]Case Western Reserve Univ, Cleveland, United States
[2]Yale University, New Haven, United States

**LEVEL-III**

*Purpose*: Conventionally held risk factors for Femoro-acetabular Impingement (FAI) include femoral and acetabular retroversion with the theory that relative retroversion leads to impingement and secondary cam deformity. We hypothesized that cam deformity is mostly primary in etiology and not associated with these risk factors. The purpose of this study was to utilize multiple regression to assess the relationship between femoral and acetabular version with two markers of cam deformity, alpha angle and anterior offset.
*Methods:* We randomly selected 1013 cadaveric hips from a historical osteologic collection. Hemipelves were reassembled with sacra to reproduce the pelvic ring. Acetabular version was then directly measured from specimens in a standardized fashion. Digital images were obtained of each femur from an axial view perpendicular to the femoral neck in order to measure alpha angle and anterior offset. Cam deformity was defined as alpha angle greater than 60°. A direct axial view of the femur was also obtained in order to measure femoral version. Multiple regression analysis was performed to determine whether alpha angle or femoral offset are related to age, femoral version, acetabular version. Significance level was set at $p < 0.05$.
*Results:* The mean alpha angle and anterior offsets for the sample population were $48.1° \pm 10.4°$ and $0.76 \pm 0.18$ cm, respectively. 223/1,013 specimens emonstrated cam deformity (alpha angle >60°). Multiple regression analysis did not demonstrate any statistically significant association between femoral version, acetabular version, and alpha angle. However, multiple regression analysis demonstrated a small, but significant association between increasing femoral and acetabular version with decreased anterior offset (both $p < 0.01$). While this relationship was statistically significant, its clinical relevance was mild as all factors combined only explained 9 % of the variance in anterior offset.
*Conclusion:* Conventionally accepted risk factors for development of cam deformity include relative femoral and/or acetabular retroversion. Our study did not observe these associations in a large and random population. Our data suggests that Cam deformity is generally a primary lesion rather than a contre coup lesion secondary to impingement. It is important to note that our population presumably did not have any increased FAI symptomatology as compared to any random sampling. Given this, we propose that relative acetabular or femoral retroversion may instead increase the risk for a symptomatic hip by mechanically increasing the risk for impingement.
*Significance:* An evolving understanding of the aetiology of FAI may lead to changes in evaluation and management of adolescents and young adults with this pathology.

April 16
Scientific session: Basic science
14:25–15:45
AUDITORIUM
**OP31**/15:23–15:29

## The importance of lower extremity proportions during limb lengthening or reconstructive surgery*

Douglas Weinberg[1], Raymond Liu[1]

[1]Case Western Reserve Univ, Cleveland, United States

**LEVEL-II**

*Purpose*: When individuals with asymmetric lower extremities present for evaluation for limb lengthening, correction can occur at the femur, tibia, or in both bones simultaneously. The aim of this study is to examine the normal ratio of tibia length/femur length, and to explore the consequences of lengthening the tibia relative to the femur on adjacent joint arthritis.
*Methods:* Bone lengths of 1,192 cadaveric femora and tibiae from the Hamann-Todd osteologic collection were measured using a digital ruler. Each femur was measured from the superior aspect of the femoral head to the femoral condyles. Tibiae were measured from the lateral tibial plateau to the lateral tibial plafond. Degenerative disease of the hip, knee and spine were from 0 to 6 for the hip and knee, and from 0 to 4 for the spine. Specimens with obvious fracture, infection, or rheumatologic conditions were excluded from study. Correlations between the ratio of tibia/femur lengths and osteoarthritis in the ipsilateral hip, knee and spine were evaluated with multiple regression analysis.
*Results:* The average ratio of tibia to femur length (T/F), was $0.79 \pm 0.13$. The average patient age was $56 \pm 10$ years. There were 82 females and 514 males, 185 African-Americans, 409 Caucasians, and 2 other ethnicities. Average grades for hip, knee, and spine OA were $3.3 \pm 1.5$, $3.4 \pm 1.6$, and $2.5 \pm 1.2$, respectively. There was a strong correlation between age and arthritis at all sites, with standardized betas ranging from 0.45 to 0.54 (P
*Conclusion:* Increasing tibia length relative to femur length was found to be a significant predictor of ipsilateral hip arthritis, and approached significance for arthritis of the spine and ipsilateral knee. Therefore, we recommend that when performing limb lengthening, surgeons should plan to recreate the normal ratio of 0.79. In situations where one bone is to be over-lengthened relative to the other, some preference should go towards over-lengthening the femur.
*Significance:* This study demonstrates that over-lengthening the tibia may predispose individuals to hip, knee and spine arthritis.

## April 16

Scientific session: Basic science
14:25–15:45
AUDITORIUM
**OP32**/15:29–15:35

### BMP-2 and ZA combination treatment for congenital pseudarthrosis of the tibia in neurofibromatosis type 1*

Nikita Deo[1], Tegan Cheng[1], Aaron Schindeler[1], David Little[1]

[1]Westmead Children's Hospital, Sydney, Australia

**LEVEL-I**

*Introduction/aims:* Congenital pseudarthrosis of the tibia (CPT) is a severe orthopaedic complication related to Neurofibromatosis type 1 (NF1) where healing following fracture is recalcitrant. We have recently created a mouse model of CPT by knocking out the NF1 gene at the fracture site. The model bears all the hallmarks of CPT, including poor bone formation, excessive osteoclast-driven bone resorption and ingrowth of fibrovascular tissue. We hypothesised that recombinant human bone morphogenetic protein-2 (rhBMP-2) co-treated with the bisphosphonate zoledronic acid (ZA) would improve bone union and strength in the model.
*Methods:* To model the genetics and injury associated with an NF1 pseudarthrosis, tibial fractures made in *Nf1flox/flox* mice were treated with Cre-expressing adenovirus to induce local double inactivation of the *Nf1* locus at the site of a fracture stabilised by intramedullary fixation. Adjunctive interventions included 10 μg local rhBMP-2/ACS (Medtronic) with/without $5 \times 0.02$ mg/kg systemic ZA starting from day 3 post-op ($n = 15$/group). Key outcomes were radiographic (x-ray, micro-CT), and mechanical (Instron) at 3 weeks post-fracture.
*Results:* Animals treated with rhBMP-2/ZA in combination showed the highest rate of bone union (93 %) compared to vehicle (7 %*), ZA (0 %*), and rhBMP-2 alone (86 %) (*$p < 0.01$). Treatment with rhBMP-2 produced a 2-fold greater increase in bone volume compared to ZA** and a 3-fold increase compared to vehicle** (**$p < 0.01$). Co-treatment with ZA led to further significant increases in bone volume compared to vehicle**, ZA** and rhBMP-2** (**$p < 0.01$). The vehicle and ZA alone groups showed negligible union, but rhBMP-2 and rhBMP-2/ZA groups could be compared by four-point bending mechanical testing. The rhBMP-2/ZA treated fracture strength had returned to the same as the non-fractured side and were twice as strong as those treated with rhBMP-2 alone ($p < 0.01$). Significantly, the amount of fibrous tissue invasion was increased by rhBMP-2 treatment; this negative effect was abrogated by the addition of zoledronic acid.
*Conclusions:* Our results from our genetic preclinical model are consistent with a preliminary CPT case series receiving this combination treatment. By utilising BMP to increase bone anabolism and ZA to reduce catabolism, a more robust union can be achieved in the short term, with minimal fibrous tissue invasion. Longer term experiments are underway, the results of which could inform clinical trials with rhBMP-2/ZA co-treatment versus standard of care.

## April 16

Scientific session: Limb reconstructions
16:15–17:30
AUDITORIUM
**OP33**/16:15–16:21

### Limb lengthening after internal hemipelvectomy with the Fitbone®-System

Rainer Baumgart[1]

[1]ZEM-Germany, Munich, Germany

**LEVEL-IV**

Internal hemipelvectomy after resection of malignant bone tumors of the pelvis is one option accepting shortening of the leg which may be aggravated by the growth of the contralateral side in the case of children, so that at maturity a huge limb length discrepancy results. The method of callus distraction offers the potential of bone growth of high biological quality. Using fully implantable distraction nails (Fitbone), lengthening can be controlled by wireless using an external control unit, and the risk of infection is minimal. Exercises can be done without restrictions to prevent contractures, stiffness and subluxation of the adjacent joints which are set under high pressure. In cases of internal hemipelvectomy with or without arthroplasty, the neo-acetabulum is set under high load with an unknown risk of subluxation or even luxation if lengthening of the femur is performed. Would it be an option to use a fully implantable distraction nail also under these circumstances? What perspective can be expected?

In 5 patients (2 male, 3 female) mean age 16.8 years, limb lengthening was performed with a fully implantable system (Fitbone) after resection of a malignant bone tumor of the pelvis which was treated by an internal hemipelvectomy. In all cases the lengthening nail was implanted retrograde through the knee joint. The osteotomy was performed about 9 cm proximal to the knee joint level. In 2 cases another distraction nail was implanted in the tibia and lengthening was performed simultaneously.
The mean lengthening achieved was 10.5 cm (femur 8 cm, tibia 3.6 cm). The mean follow up was 28 months (12/42) after the completion of lengthening. No technical complication occurred. In all cases bone formation was circular and sufficient. The position of the hip joint/prosthesis and the functional use remained unchanged.
From our experiences limb lengthening of the femur and tibia with a fully implantable distraction nails (Fitbone) seems to be suitable and effective even after internal hemipelvectomy with or without arthroplasty. If the joint was able to carry full load before for at least 2 years, the lengthening process do not increase the tendency of luxation.

**April 16**
Scientific session: Limb reconstructions
16:15–17:30
AUDITORIUM
**OP34**/16:21–16:27

## Assessing effect of the level of osteotomy in femoral lengthening using monolateral fixator*

Kwang-Won Park[1], Ji-Ho Lee[1], Han-Ju Kim[1], Hae-Ryong Song[1]

[1]Korea University Guro Hospital, Seoul, Republic of Korea

**LEVEL-III**

*Introduction:* Femoral lengthening by distraction osteogenesis is an established procedure for correction of limb length discrepancy. However, very few studies have examined the effect of the level of osteotomy in femoral lengthening. We aimed to determine whether a different level of osteotomy in the femur affects the radiographic outcome, clinical outcome, and incidence of complications.
*Materials and methods:* Isolated femoral lengthening with a monolateral fixator was performed in 51 patients (mean age 13 years, range 5–22 years). Patients were categorized into the following 3 groups according to the level of osteotomy; group A (proximal, 11 patients), group B (middle, 25 patients), and group C (distal, 15 patients). Preoperative and post-operative radiographic parameters, clinical outcome, and surgery-related complications were analyzed.
*Results:* Radiographic outcomes are not significantly different for axial alignment indices, callus progression patterns, lengthening percentage, lengthening index, external fixation index, and maturation index between any of the groups ($p > 0.05$). 1 month after surgery, 43 % of patients in group A, 46 % in group B, and 80 % in group C demonstrated a limited range of knee of motion (ROM) less than 80°. At 1 year after surgery, all patients in groups A and C demonstrated full knee ROM whereas 4 patients (30 %) in group B demonstrated limited knee ROM up to 100°. Transient postoperative limited hip ROM during the first 3 months was observed only in group A. When analyzing postoperative complications, group C had the greatest complication rate of 9.3 per segment, most of which (53 %) required surgical intervention.
*Conclusion:* Different levels of osteotomy in femoral lengthening do not show any significant difference in radiographic parameters. Clinically, distal osteotomy was associated with greater restriction of knee ROM and demonstrated the greatest number of complications requiring surgical intervention. Our study suggests that proximal and middle osteotomies are preferable to distal osteotomy in femoral lengthening.

**April 16**
Scientific session: Limb reconstructions
16:15–17:30
AUDITORIUM
**OP35**/16:27–16:33

## Results of deformity correction of lower limbs in children with X-linked hereditary hypophosphstemic rickets

Dmitry Popkov[1], Arnold Popkov[1]

[1]Ilizarov Center, Kurgan, Russian Federation

**LEVEL-III**

*Introduction:* Relapses of deformities after reconstructive surgeries are observed in children with X-linked hereditary hypophosphstemic rickets. The retrospective study is aimed to compare the short and long term follow up results of the surgery performed with Ilizarov fixator or the combined technique (Ilizarov device + Flexible Intramedullary Nailing).
*Materials and methods:* The results of two retrospective series were compared in short (6 months) and long (over 5 years) term follow up. Corrections at the femoral and tibial levels performed in the single-event surgery were systematic. In series I (22 children, 10.7 years old, 44 limbs) osteosynthesis was performed with Ilizarov fixator. In series II (25 children, 8.9 years old, 50 limbs) Ilizarov device was combined with FIN. The intramedullary nails with chitosan/calcium phosphate covering were used. The results of series were compared by parameters of duration of ExFix, rate of complications, integral results (Lascombes, 2012) and relapses of deformity in long term follow up.
*Results:* In all the patient the planned correction was achieved. In series I the external osteosynthesis lasted 124.7 days on average. Combined Ilizarov + FIN reduced the duration of osteosynthesis to 87.4 days. Series I: local infection problems (24 cases), transient paralysis (6 cases), secondary displacement requiring modification of external osteosynthesis under GA (8 cases) were observed. Series II: local sepsis (16 cases), transient paralysis (7 cases), irritation of soft tissues by the FIN nails (2 cases). According to the Lascombes classification the ilizarov fixator alone provided 30 results of category I, IIa = 12 cases, IIb = 2 cases. The combined technique improved the results: I = 46, IIa = 4. In the long term there were no deformity in 6 limbs (13.6 %) in series I and 10 limbs (20 %) in series II. In other patients deformities reappeared. Their charecter was not identical. In series I the CORA levels were mi-diaphyseal and metadiaphyseal. In series II—diaphyses reinforced with nails remain straight, but deformities appeared at the metaphyseal level beyond the FIN zones.
*Conclusion:* The combined technique (ExFix + FIN) allows restoration of the mecanical axis, and reduces the duration of external fixation. To decrease the number of complications in children with

X-linked hypophosphstemic rickets, the application of FIN preventes bone deformities along the nails in long term follow up.

**April 16**
Scientific session: Limb reconstructions
16:15–17:30
AUDITORIUM
**OP36**/16:41–16:47

### Plate size and orientation influence rotational correction in femoral rotational guided growth: a biomechanical study*

Ryan Li[1], Raymond Liu[1]

[1]Case Western Reserve Univ, Cleveland, United States

**LEVEL-IV**

*Background:* While guided growth is widely used to correct coronal and sagittal plane deformities in the skeletally immature patient, the use of 8-plates to correct rotational deformities has been less studied. Arami et al. showed that placing 8-plates oblique to the growth plate could result in rotational deformity. However, there is no present study investigating timing of rotational guided growth and the relationship between plate characteristics and rotational correction.
*Methods:* A mathematical model for rotational guided growth in the femur was adapted from Arami et al. Plate length and orientation were found to influence rotational correction. Maximum rotational correction and progressive rotational correction over 1 mm increments of simulated growth were calculated for 12 and 24 mm plates at 30° and 60° offset angles. A biomechanical sawbones model was constructed to validate our mathematical model. Angular correction was assessed using guide pins placed on either side of a simulated growth plate. The femur was progressively lengthened at the growth plate to simulate growth. Progressive and maximal rotational correction were investigated for the conditions described. Intraclass correlation coefficient (ICC) was used to calculate agreement between mathematical and biomechanical models.
*Results:* Our mathematical model predicted that increased offset angle and plate length would increase rotational correction. Plates offset at 60° from vertical had 1.75 times the rotational correction to 30° offset plates. Increases from 12 to 24 mm plates increased rotational correction by a factor of 2. Increasing both variables resulted in a 3.5 times increase in maximum rotational correction. Using the biomechanical model, 2.2 and 1.7 times maximal rotational correction were achieved with increases in plate offset and plate length respectively. Simultaneous increase in both resulted in a 4.1 times increase in maximal rotational correction. Both mathematical and biomechanical models predicted non-linear increase in rotational correction with growth. In both models, 55–75 % of total rotational correction was achieved in the latter half of simulated growth. ICCs between mathematical and biomechanical models ranged from moderate (0.42, 0.44) to excellent agreement (0.93, 0.95).
*Discussion:* This is the first study to validate the relationship between plate length with rotational correction, as well as the first study to propose a non-linear relationship between growth and correction in rotational guided growth. Further investigation is required to clarify these relationships prior to clinical application of this technique.

1. Arami et al. "guiding femoral rotational growth in an animal model." *Journal of Bone and Joint Surgery* 2013 95:2022–7

**April 16**
Scientific session: Limb reconstructions
16:15–17:30
AUDITORIUM
**OP37**/16:47–16:53

### FlexTack™ for Temporary Hemiepiphysiodesis: simpler implantation, less fluoroscopy and faster correction compared to the eight-plate™*

Bjoern Vogt[1], Henning Tretow[1], Georg Gosheger[2], Melanie Horter[1], Frank Schiedel[1], Robert Roedl[1]

[1]Children's Orthopaedics, UKM, Muenster, Germany
[2]General Orthopaedics, UKM, Muenster, Germany

**LEVEL-II**

*Introduction:* Hemiepiphysiodesis is the first-line-treatment for coronal angular deformities of the knee in growing children. Modern implants like the eight-PlateTM solved many problems that were observed using staples for temporary hemiepiphysiodesis. Rather than creating rigid compression forces on the growth-plate like staples, the eight-Plate™ causes a shift of the fulcrum for correction outside the growth-plate creating a flexible tension-band-effect for guided growth. However, implant-design, surgical technique and biomechanical alignment of the plate remain suboptimal leading to implant-associated complications and biomechanical problems like delayed onset of correction.
*Aim of the study:* Therefore our group developed a new device for temporary hemiepiphysiodesis that consists of an anatomically preformed staple with cannulated legs ensuring the simple and precise implantation-technique of the eight-Plate™. The 13°-trapezoid-shaped design properly fits to the anatomic shape of the medial and lateral aspect of the distal femur and the proximal tibia. The staple provides a flexible bar (FlexTack™) that permits the extraphyseal tension-band-effect by analogy with the eight-Plate™.
*Patients and methods:* A prospective cohort of 62 patients (-male = 44, female = 18; age ≥12.2 years) with 153 consecutive FlexTack™-implantations for correction of valgus (n = 41, FlexTacks™ = 99), varus (n = 12, FlexTacks™ = 27), flexion (n = 2, FlexTacks™ = 6) and limb length discrepancy (n = 7, FlexTacks™ = 21) with a follow-up of >4.5 months (max. 1.0 year) was compared to a historical cohort of 93 patients (male = 53, female = 40; age ≥11.7 years) with 246 eight-Plate™-implantations with a follow-up of >1.0 year (max. 2.4 years). Intraoperative parameters like time for surgery (cut–suture) and for fluoroscopy were assessed. Standardized clinical and radiographic follow-up-examinations every 3–6 months were performed. Correction-speed (MAD-correction/month/hemiepiphysiodesis-location) was analysed in the valgus-group. Complication rates were evaluated focusing implant associated and biomechanical problems. Statistical analysis was done using Mann–Whitney-U-Test and Fisher's-Exact-Test.
*Results:* Compared to the eight-Plate™ time for implantation and for fluoroscopy were significantly shorter using the FlexTack™ [22.4 and 36.8 min., resp. ($P < 0.001$); 0.27 and 0.42 min., resp. ($P < 0.001$)]. Earlier onset and significantly faster speed of valgus-correction were measured using the FlexTack™ [1.59 mm/month and 3.30 mm/month, resp. ($P < 0.001$)]. Common complications like wound-infection required revision [1.6 and 1.1 %, resp. ($P = 1.000$)], hematoma/effusion [6.4 and 3.2 %, resp. ($P = 0.439$)] and neurovascular damages [0 and 0, resp. ($P = 1.000$)] were comparable. However, implant-associated problems like bending, breakage, loosening and soft-tissue-irritation were not observed using the

FlexTack$^{TM}$ during follow-up compared to a rate of 10.8 % in the eight-Plate$^{TM}$-group (P = 0.006).
*Conclusion:* The FlexTack$^{TM}$ is a reasonable synthesis of staples and canullated screw/plate-devices. The implantation is simpler which reduces time for surgery and radiation-exposure. Due to the anatomical shape and biomechanical improvements faster corrections and lower rates of implant-associated problems could be achieved. However, these are preliminary results with a short-term follow-up.

**April 16**
Scientific session: Limb reconstructions
16:15–17:30
AUDITORIUM
**OP38**/17:01–17:07

### The role of intramedullary alignment in correction of limb length discrepancies using an ilizarov method in children

Bojan Bukva[1], Radivoj Brdar[1], Sinisa Ducic[2], Dusan Abramovic[1], Vladimir Radlovic[1], Goran Djuricic[1]

[1]University Children‘s Hospital, Belgrade, Serbia; [2]University Children‘s Hospital, Beograde, Serbia

**LEVEL-III**

*Background:* The purpose of this study was to evaluate the influence of the intramedullary aligment with Kirschner (K) or Titanium Elastic (TE) wires combined with an Ilizarov external fixator (IEF) on the healing index, lengthening index, duration of hospital treatment and complications in congenital and acquired leg discrepancy.
*Methods:* This study included 73 paediatric patients, aged 4–18 years, over a 14 years period. We compare the healing index (HI), lengthening index (LI), duration of hospital treatment and complications between two groups of children. The first group of children undergoing limb lengthening by the IEF alone (group I). The other group (group II) was treated with the combination of the IEF and intramedullary stabilization using two K-wires or TE-wires. Also we compare all of following parameters depending of the type of intramedullary alignment (K or TE wires).
*Results:* We found significant differences between Group I and Group II in the duration of external fixator application ($p < 0.01$), HI ($p < 0.05$) and duration of hospitalization ($p < 0.05$). Concerning leg length inequality, we found no significant differences between the two groups of patients. Also, we found no significant differences in complication rate and etiology of LLI (congenital or acquired) between two groups of patients. Depending of the type of intramedullary alignment we found less complications and shorter period of intrahospital treatment in patients treated using in combination of IEF and TE wires.
In statistical analysis we used methods of descriptive and analytical statistical analysis (Pearson c2 test; Fisher exact test; Kruskal–Wallis test; Wilcoxon rank sum test with continuity correction). Statistical significance was set on $p < 0.05$.
*Conclusion:* The intramedullary alignment has multiple advantages as a method of treatment of the limb discrepancy. The major effect of the application of the combination of external circular fixation and intramedullary alignment is a significant decrease of external fixation duration and healing index. This method of the treatment decreases hospital costs.
*Keywords:* Leg length discrepancy, Intramedullary alignment, Ilizarov external fixator, children

**April 16**
Scientific session: Limb reconstructions
16:15–17:30
AUDITORIUM
**OP39**/17:07–17:13

### Results of bone lengthening in transverse deficiency and congenital amputation of the forearm in children

Dmitry Popkov[1], Eugeny Grebenyuk[1], Arnold Popkov[1]

[1]Ilizarov Center, Kurgan, Russian Federation

**LEVEL-IV**

*Introduction:* Transverse deficiency and congenital amputation of the forearm are observed more frequently at the level of proximal or medial third of the forearm. Bone lengthening of stump finds its indications in cases of a short segment to facilitate prosthetic procedures. The aim of this retrospective study is to evaluate results of bone lengthening in patients affected with forearm deficiency.
*Materials and methods:* The series contains 17 children between the age of 6–15 years old. The mean initial length of ulna was 6.2 cm, radius—3.8 cm. Lengthening was performed by means of the Ilizarov fixator. In three patients a limited resection of distal sharp ends of two bones and distal synostosis between radius and ulna was performed at the same surgical procedure with application of the Ilizarov fixator. This was necessary in order to prevent perforation of the soft tissues at the apex of the stump during lengthening. In one case a combined technique (external fixator + flexible intramedullary nailing) was performed. The patients were regularly observed within the mean follow up term of 25.5 months.
*Results:* The mean gain of lengthening was 4.3 cm with the average healing index of 34.2 day/cm (from 15.4 to 44.7 day/cm). the rate of lengthening was 0.8 ± 0.7 mm/day. The following complications were observed in the series: local infection problems (13 cases), incomplete osteotomy of the radius (1 case), loss of transosseous position of a wire in the distal ring of external fixator (3 cases), non-union resulting in pseudoarthroses of the radius (1 case). In the long term follow up recovery of range of motion in the elbow was noted in 16 patients. One child manifested extension deficiency of 25°. All the patients stopped the use devices at the level of elbow and started to use a prosthesis fixed at the level of lengthened forearm allowing natural articulation of the elbow.
*Conclusion*: Progressive lengthening of the forearm in children with transverse deficiency and congenital amputation of the forearm is justified in order to facilitate prosthetic procedures. The necessary amount of lengthening can be achieved with one surgery with a low rate of complications.

**April 16**
Scientific session: Limb reconstructions
16:15–17:30
AUDITORIUM
**OP40**/17:13–17:19

### Is growth trajectory as assessed by the multiplier method affected by physiological age?*

James J Aird MBBS, MD, FRCS (Orth)[1], Caroline Chessman BSc MBChB MRCS(Eng)[2], Alexander T Schade BSc[2], Fergal P Monsell BCh, MSc, PhD, FRCS, F[2]

[1]Sick Kids, Toronto, Canada
[2]Bristol Royal Hosp Children, Bristol, United Kingdom

**LEVEL-II**

*Introduction:* Paley et al. has developed a multiplier method for calculating both leg length and total height. In the development of this algorithm, they evaluated the effect of factors including bone age and sex. They established that sex had a significant impact, but adjusting for bone age did not improve accuracy. Bone age and menarche have been shown to improve other height prediction models.
*Purpose*: We used a large prospective cohort to evaluate if the multiplier is independent of physiological age using menarche as a proxy.
*Methods:* Using the ALSPAC dataset we determined the accuracy of the Paley multiplier for predicting total height and leg length, and assessed weather if the date of first menses increased the accuracy of the multiplier. Female patients over the age of 8, with documented final height and final sub-ishial leg length over the age of 15 and a date of first menses were evaluated. Predicted final height was compared with actual final height at all data points.
*Results:* There were 28,332 data points in 3,062 girls prior to skeletal maturity in the total height cohort and 8,395 data points in 2,300 girls in the leg length cohort. When age was corrected using the difference in age at onset of menarche from average, the accuracy of multiplier decreased for both measurements When a correction of 50 % was used, there was an improvement in the accuracy of multiplier predictions, reducing the average error by up to 24 %.
*Conclusions:* Previous studies have failed to demonstrate that the accuracy of the multiplier is improved when adjusted for bone age. We have used the date of first menses as a proxy for bone age and established that making a 50 % correction for physiological age improves the accuracy of this method.
*Significance:* This will potentially allow more accurate prediction of leg length discrepancy, and total height in girls with early menarche.

**April 17**
Scientific session: DDH
08:00–09:00
AUDITORIUM
**OP41**/08:00–08:06

## Efficacy of selective ultrasound hip screening in developmental dysplasia of the hip based on traditional risk factors*

Clare Taylor[1], Nadia Sciberras[2], James Metcalfe[2], Rima Vaikute[2], Benjamin Holroyd[2]

[1]Plymouth Hospitals NHS Trust, Plymouth, United Kingdom
[2]Plymouth Hospitals NHS Trust, Plymouth, United Kingdom

**LEVEL-IV**

*Introduction and background:* Universal hip screening for developmental dysplasia of the hip (DDH) using ultrasound despite its widely accepted clinical and economic benefits in the United States has yet to be implemented in most westernised countries.Screening policies in the UK vary but are usually directed towards children perceived to be at increased risk of DDH in an attempt to reduce the incidence of surgery. The ‘at risk’ factors in the aetiology of DDH have traditionally included female sex, first born, high birth weight, post term, breech presentation and family history. It is reported that up to 40 % of children diagnosed with DDH have one or more of these risk factors. We have undertaken a retrospective study to quantify the relationship between these ‘at risk’ factors and the presence of DDH in the population presenting to a tertiary referral centre.
*Materials and methods:* From 2008 to 2013 we examined all paediatric hip ultrasound scans (USS) performed at our centre. These included patients referred with recognized ‘at risk’ factors and those with clinical signs of instability when routine neonatal baby checks were performed. All USS were performed by one of the senior authors (RV).
*Results:* During the study period there were 28,365 live births in the catchment area. There were 2,526 (8.9 %) babies (1,391 females, 1,135 males) referred to the service with ‘at risk’ factors or clinical signs of instability. A diagnosis of DDH requiring treatment was made in 175 hips (6.2 per 1,000 live births); 86 % girls, 14 % boys.Statistical analysis was carried out to determine the odds ratios for each of the ‘at risk’ variables. In our population first born (OR 2.221, $p < 0.001$), sex (OR 0.188, $p < 0.001$), Birth weight > 4.5 kg (OR 0.096, $p = 0.001$) and twins (OR 0.096, $p < 0.001$) were significantly associated with the diagnosis of DDH. Of interest ‘at risk’ factors that are traditionally thought to be highly predictive of DDH, family history, breech presentation and diagnosis of talipes were not significantly associated with a diagnosis of DDH.
*Conclusion:* We believe that current targeted screening based on traditional risk factors correlates poorly with the diagnosis of DDH in our population. Our reported incidence of DDH (6.2 %) is higher than that reported by similar previous studies, principally due to a higher percentage screening rate (8.9 %).
*Significance:* This adds further support to the argument for adopting universal screening, or at the very least targeted screening based on contemporary evidence of risk factors.

**April 17**
Scientific session: DDH
08:00–09:00
AUDITORIUM
**OP42**/08:06–08:12

## A Combine technique of a multi images hip sonography: better understanding of hip physiology

Blazej Pruszczynski[1], Marek Synder[1]

[1]Medical University of Lodz, Lodz, Poland

**LEVEL-III**

*Background:* The DDH screening examination is performed by orthopaedic surgeons, radiologists, paediatricians and sometimes other doctors. Graf technique for hip sonography is widely used in our medical care system. In difficult or doubtful cases other sonogarphy technique may be helpful for decision making regarding treatment necessity. The dynamic, multi-images technique was already develop by Harcke. Many authors proposed their own techniques.
*Materials and methods:* The aim of the study was to analyze how well known multiple slices sonography techniques of the hip joint are helpful in the decision making for hip treatment. Standard Graf technique was used for sonography. However in doubtful cases other technique were performed: Harcke (multi-plane, static and dynamic), Bouquet (medial) Dalstrom (anterior).
*Results:* From January 2014 to October 2014 two thousand infants were seen for newborn DDH screening. In 8 infants at least one hip were classified as Type D or more severe according Graf and required treatment. Twenty five infants were diagnosed with at least one hip type IIa, increased internal rotation, or a subtle Barlow test. These children were re-examined with other sonography techniques. Excessive laxity was the most frequent diagnosis. 4 of 25 discreet decentralizations (according Harcke and Bouquet techniques) were noted. All but one resolved with an abduction exercises protocol. Another 15 infants were diagnosed with Type IIb and the alfa angle between 55 and 59 at 3 month or older, other sonography techniques did not reveal any abnormality. With the use of Harcke and Bouquet techniques the evaluation of infant hips in Pavlik harness become easier.
*Discussion:* This is a preliminary report of combined imaging possibilities using sonography of the infant hip. Based on 2D techniques we can build a 3D impression of the joint. Multiple slices allows the physician to have a better impression of the morphology and stability of the infant hip. This was already described by Harcke.
*Conclusion:* The decision for starting treatment should be taken after full visualization of the hip joint and should not depend on one static image. By using static and dynamic views in coronal, transvers, medial and anterior plane, the decision is more reliable. The repeated standard measurement in one view may lead to underdiagnosis or overtreatment in some cases. This is similar to only one plane hip radiography.

**April 17**
Scientific session: DDH
08:00–09:00
AUDITORIUM
**OP43**/08:12–08:18

## Pavlik harness treatment has tendency to fail considerably over 4 months of age and in dislocated hips in developmental dysplasia of the hip

Hakan Ömeroglu[1], Nusret Köse[1], Anil Akceylan[1]

[1]Eskisehir Osmangazi University, Eskisehir, Turkey

**LEVEL-IV**

*Objective:* The aim of this report was to assess the effect of age, gender, laterality, coexisting risk factors and ultrasonographic hip type on the success rate of Developmental Dysplasia of the Hip (DDH) treatment by the Pavlik harness within the first 6 months of life.
*Methods:* This study included 171 hips of 122 children (100 girls and 22 boys) with a mean age of 107 days (22–197) who were initially treated by the Pavlik harness within a 2.5 years period. The hips were classified according to the Graf's ultrasonographic hip classification system and the Pavlik harness was applied for Graf type IIa- and worse hips. The parents were clearly informed about the use of harness and were not allowed to remove it. The treatment was considered unsuccessful if an initially decentred hip (type D) or dislocated hip (types III and IV) did not progress into a better hip type within each 3–4 weeks period of follow-up when the type of treatment was changed. If an initially dysplastic hip (types IIa-, IIb and IIc) did not improve a period of 8 weeks,another treatment method was applied. Abandonment of the Pavlik harness treatment due to femoral nerve palsy was also considered as "failure of the treatment".
*Results:* There were 28 type IIa, 88 type IIb, 16 type IIc, 16 type D, 17 type III and 6 type IV hips. The treatment was successful in 86 of 122 children (71 %). There were two children with unilateral femoral nerve palsy. Age was considered the most significant patient factor for the treatment success (0–60 days; 82 %, 61–120 days; 83 %, >120 days; 48 %, P < 0,001). Gender, laterality and coexisting risk factors such as family history, breech presentation, first born girl, swaddling, intrauterine packing were not correlated with the success rate of Pavlik harness treatment (P > 0,05). The treatment success rate significantly diminished with the worsening of the ultrasonographic hip type (IIa; 96 %, IIb; 77 %, IIc; 68 %, D; 56 %, III; 29 %, IV; 33 %, P < 0.001).
*Conclusion:* Pavlik harness treatment seems to be more favourable for the patients younger than 4 months of age and in stable hips as, the treatment tends to fail in one half of the patients over 4 months of age and in about two thirds of the dislocated hips.
*Clinical relevance:* The use of Pavlik harness over 4 months of age and in unstable hips needs to be revisited.

**April 17**
Scientific session: DDH
08:00–09:00
AUDITORIUM
**OP44**/08:26–08:32

## Can anterior hip ultrasound reliably assess hip reduction in a spica cast?

Oliver Birke[1], Justine St George[1], Viktoria Leon[1], David Bradshaw[1], David Little[1]

[1]Westmead Children's Hospital, Sydney, Australia

**LEVEL-IV**

*Purpose*: Currently most centres utilise post-operative MRI or CT to confirm hip reduction following closed reduction and spica casting in developmental dysplasia of the hip (DDH). However MRI and CT are both costly and potentially result in a return to theatre. This study aims to validate the use of perioperative, clinician directed anterior hip ultrasound (AHUS), as a means of imaging the position of the femoral head immediately after spica application during the same anaesthesia.
*Method*: Operation records over a 5 year period (2009–2014) were reviewed and 49 cases were identified in which AHUS was used to confirm the position of femoral head following closed reduction and spica application in children with frank hip dislocations. AHUS, as described by Van Douveren et al. utilises a small curved ultrasound probe and produces an image comparable to an axial CT or MRI. The AHUS was performed directly after spica application during the same anaesthesia and the documented results were compared to the subsequent formal post-operative imaging which included magnetic resonance imaging (MRI), computed tomography (CT), formal lateral ultrasound (US) or X-ray.
*Results:* 46 patients (49 hips) were included [mean age 6.1 months (1.6–20.9)]. Subsequent imaging was performed post-operatively in 9 cases (18.5 %) or at time of first follow-up in the remainder of the cases (81.5 %). The findings of the perioperative AHUS positively correlated with subsequent imaging in 98 % (48/49) of cases. Only one hip was found to be dislocated 6 weeks postoperatively on subsequent theatre fluoroscopy during the change of spica. This patient was noted to have a very unstable hip with a "narrow safe zone" at reduction, and had previously failed a closed reduction attempt. In this case the AHUS was either inaccurate at the time of spica application or the very unstable hip subsequently re-dislocated during the 6 week period in spica. In the remaining 48 cases, the femoral head position was accurately assessed and confirmed on subsequent imaging (2/2 dislocated, 46/46 located).
*Conclusion:* AHUS is a reliable, safe and inexpensive technique to confirm femoral head reduction in a spica cast. This enables the surgeon if necessary to act immediately during the same anaesthesic, potentially saving a return to theatre. More expensive post-operative imaging such as MRI can be reserved for equivocal cases. For very unstable hips we suggest a repeat AHUS prior to discharge and early follow-up with AHUS performed in a clinic setting.

**April 17**
Scientific session: DDH
08:00–09:00
AUDITORIUM
**OP45**/08:32–08:38

## Is an AP Pelvis x-ray enough to judge adequacy of hip reduction in spica cast?

Cristina Alves[1], Federico Canavese[2], David Lebel[3], Walter Truong[4], Emily Dodwell[5]

[1]Hospital Pediátrico- CHUC, EPE, Coimbra, Portugal
[2]CHU Estaing, Clermont Ferrand, France
[3]Dana-Dwek Children's Hospital, Tele-Aviv, Israel
[4]Gillette Childrens Hospital, Minneapolis, United States
[5]Hospital for Special Surgery, New York, United States

**LEVEL-II**

*Purpose*: To determine the efficacy of using AP Pelvis X-Ray to detect the adequacy of hip reduction in spica cast, in children aged 4–10 months, diagnosed with developmental hip dysplasia (DDH), undergoing closed reduction of at least one dislocated hip.
*Methods*: Three fellowship-trained pediatric orthopaedic surgeons from three different institutions rated post-operative in-spica AP Pelvis XRays of 25 children with DDH, treated in two other institutions, by closed reduction of at least one hip. Raters were not involved in the care of these children and were blinded to all information regarding them. Each surgeon evaluated XRays at two different time points, with a minimum 2 weeks interval between evaluations. The XRays were re-ordered for second evaluation. Surgeons were asked to rate each hip as 'in' (reduced hip) or 'out' (dislocated hip). All children had a post-operative MRI (after the XRay), to confirm the adequacy of hip reduction. The ratings of each surgeon were compared to MRI results.
*Results*: Three surgeons reviewed in-spica AP Pelvis XRays of 25 children (50 hips). Of the hips evaluated, 19/50 were normal and 31/50 were dislocated hips that underwent closed reduction ± adductor tenotomy ± psoas tenotomy and spica cast. Post-operatively, 4 hips were dislocated in spica cast. The sensitivity of surgeons to determine correctly that a hip was dislocated in the spica cast was 100 %. All surgeons detected all the dislocated hips, but rated some well-reduced hips as 'out' (specificity ranged between 83 98 %). The intraobserver agreement was moderate (kappa 0.5205–0.6106). The agreement between each surgeon and MRI ranged from moderate (kappa = 0.4318) to excellent (kappa = 0.8780). The interobserver agreement was good in the second evaluation (kappa = 0.6384). When considering all surgeons' ratings and MRI, the agreement was good (kappa = 0.7496). Negative predictive value of in-spica Xrays was 100 %, positive predictive value 63.16 % and false positive rate 5.072 %.
*Conclusions*: This study shows that AP Pelvis XRays allow surgeons to detect 100 % of dislocated hips in spica cast. In more than 83 % of xrays, surgeons will correctly identify well-reduced hips in spica cast. However, it also shows that, based solely on XRays, surgeons will consider 5 % of adequate reductions as dislocated, which may prompt further unnecessary investigations or treatment.
*Significance*: X-ray is less commonly used to confirm adequacy of hip reduction after surgery. However, in some institutions, MRI and CT scans are not available and orthopaedic surgeons must make decisions based on clinical impressions and XRay findings.

**April 17**
Scientific session: DDH
08:00–09:00
AUDITORIUM
**OP46**/08:38–08:44

## Acetabular remodeling and role of osteotomy after closed reduction of developmental dysplasia of the hip*

Tae-Joon Cho[1], Chang Ho Shin[1], Joon Ho Kim[1], Moon Seok Park[2], Won Joon Yoo[1], Chin Youb Chung[3], In Ho Choi[1]

[1]Seoul Nat Univ Children's Hosp, Seoul, Republic of Korea
[2]Seoul Nat Univ Bundang Hosp, Seongnam, Republic of Korea
[3]Seoul Nat Univ Bundang Hosp, Seoul, Republic of Korea

**LEVEL-II**

*Introduction:* The purpose of this study was to evaluate acetabular remodeling after closed reduction (CR) of developmental dysplasia of the hip (DDH), and to delineate the role of osteotomy.
*Materials and methods:* 95 dislocated hips in 93 patients, which were treated by CR and were followed until 8 years old or older were subjects of this study. The mean age at CR was 14.1 months (3–30), and at the latest follow-up was 12.8 years (8.0–24.7). Osteotomy was performed for residual dysplasia in 34 hips (36 %) after CR at average 3.3 years of age (1.6–8.8). The non-osteotomy group included those for which osteotomy was recommended but refused by the parents. Acetabular index (AI) and center-edge angle (CEA) were measured, and presence of avascular necrosis (AVN) was evaluated using Bucholz-Ogden classification. Treatment outcome was classified as satisfactory for Severin group I and II, and as unsatisfactory for Severin group III and IV. We retrospectively analysed the correlation of the radiographic parameters, osteotomy, AVN, with the final outcome.
*Results:* Pre-reduction AI was 40.3° ± 4.4°. The Outcome at the latest follow-up was satisfactory in 76 hips (80 %) and unsatisfactory in 19 hips (20 %). Osteotomy group were significantly older age at CR ($p = 0.052$) and with an increased AI at age 2–4 years ($p = 0.002$), but showed significantly better outcome ($p = 0.042$) than non-osteotomy group. Of 35 cases which had AI ≥ 33° and CEA ≤ 15° at age 2–4 years, 22 cases underwent osteotomy and showed significantly better outcomes than 13 cases belonging to non-osteotomy group ($p = 0.02$). All the unsatisfactory cases of non-osteotomy group except one had AI ≥ 28° and CEA ≤ 15° as measured at age 2–4 years, which comprised 48.4 % of the non-osteotomy cases. 3 of 34 osteotomy cases showed unsatisfactory outcomes with

AI ≥ 34° at 1 year follow-up after osteotomy. AVN grade II to IV was observed in 11 of 96 hips (11.6 %), and showed more unsatisfactory outcomes than those without AVN (p = 0.04). Among the AVN cases, 5 of 6 osteotomy cases showed satisfactory outcome, compared with only 1 of 5 non-osteotomy cases.
*Conclusion:* From this retrospective study, the authors confirmed that osteotomy can benefit post-CR hips with poor remodeling. Osteotomy should be considered when AI ≥ 28° and CEA ≤ 15° at 2–4 years of age.
*Significance:* Continued surveillance for acetabular remodeling is required even after osteotomy. Osteotomy could also benefit the hip with AVN.

**April 17**
Scientific session: Spine Part I
08:52–09:45
AUDITORIUM
**OP47**/08:52–08:58

## Evolution of adolescent idiopathic scoliosis: results of a 20-year follow-up study*

Sébastien PESENTI[1], Benjamin Blondel[2], Emilie Peltier[1], Christian Morin[3], Jérôme Sales de Gauzy[4], Stéphane Wolff[5], Antoine Chalopin[6], Jean-Luc Jouve[1]

[1]Timone enfants, Marseille, France
[2]Timone, Marseille, France
[3]Institut Calot, Berck sur Mer, France
[4]Hôpital des Enfants, Toulouse, France
[5]Hôpital Saint Joseph, Paris, France
[6]Hôpital d'enfants, Nantes, France

**LEVEL-IV**

*Introduction:* To date, there is no consensus about the indications for treatment for patients with adolescent idiopathic scoliosis (AIS) with a curvature between 30° and 60°. Few data exist regarding the long-term outcome of patients suffering this type of deformity.
*Objective:* The objective of this study was to obtain a clinical and radiological assessment with a 20-year follow-up in patients with AIS of moderate amplitude.
*Materials and methods:* A retrospective multicentric study was lead to recruit patients who were treated for AIS, whether operated or not. Inclusion criteria were a Cobb angle during adolescence between 30° and 60°, a single main thoracic, lumbar or thoracolumbar curve and at least a 20-year follow-up. Among the data collected included the Cobb angle in adolescence and at last follow-up, the number of surgical procedures since adolescence and questionnaires of quality of life at final follow-up (SRS-22 and Oswestry Disability Index).
*Results:* A total of 258 patients were included. 100 patients who underwent surgery during adolescence, 116 who had never been operated and 42 operated in adulthood. The mean follow-up was 27.8 years. At the last follow-up, the Cobb angle progression was significantly different in the three groups (respectively 3.2° vs 8.8° vs 23.6°, $p < 0.001$). In patients who did not undergo surgery in adolescence, the Cobb angle progression at the last follow-up was significantly higher for the thoracolumbar or lumbar curves than for the thoracic curves (16.1° vs 9.7°, $p = 0.011$). In patients with lumbar scoliosis, the risk of Cobb angle progression greater than 20° was significantly higher when the initial Cobb angle was higher than 35° (OR 4.278, $p = 0.002$). The quality of life scores showed no significant difference.
*Discussion:* In this series, patients who underwent arthrodesis during adolescence had a low radiological progression with a quality of life similar to the general population, demonstrating the efficiency of surgical treatment for curvatures higher than 50°. The analysis of patients who did not undergo surgery in adolescence shows that the curves between 40° and 50° tend to aggravation with some need for surgery in adulthood. The lumbar scoliosis, which has a good reputation in childhood, showed a greater potential for worsening in the adult than thoracic scoliosis, from amplitude of 35°.

**April 17**
Scientific session: Spine Part I
08:52–09:45
AUDITORIUM
**OP48**/08:58–09:04

## Hydration properties of the lumbar intervertebral discs in ais after surgical correction: 5 years follow-up and comparison with an age-matched control group

Kariman Abelin-Genevois[1], Eva Politorszk[2], Erik Estivalezes[3], Jerome Briot[3], Pascal Swider[3], Jerome Sales de Gauzy[4]

[1]Hôpital Femme Mère Enfant, LYON, France
[2]Hôpital d'Enfants Brabois, Nancy, France
[3]Laboratory of Biomechanics, Toulouse, France
[4]Hopital des enfants Purpan, Toulouse, France

**LEVEL-II**

*Introduction:* Intervertebral discs provide the mechanical support and flexibility between vertebral segments. The supportive function is directly under the influence of the disc hydration status. With age, loss of glycosaminoglycans will induce disc dehydration which is the initial process leading to disc degeneration, compromising the hydraulic mechanism for load absorption. Scoliosis is a spinal deformity inducing a chronic asymmetric loading of the disc which may alter the disc nutrition. The aim of the present study was to quantify the disc hydration content in surgically treated patients with adolescent idiopathic scoliosis (AIS) and to compare the disc hydration properties with an age matched control group.
*Methods:* We prospectively conducted a 5-year MRI follow up of 23 AIS patients (mean age at surgery: 15, 1 years). Patients underwent pre and post operatively T2 weighted MRI turbo spin echo sequences with thin joined sagittal cuts. A specific software was developed with Mathlab® in order to reconstruct the disc and nucleus volumes (Vd, Vn). Hydration content was determined by the ratio Vn/Vd. A comparative control group of 20 patients matched by age was selected in the PACS database. Exclusion criteria were: age <10 and >16 years, spinal deformity and any disease affecting the spine. Disc volumetry was obtained using the same protocol.
*Results:* In the control group, volumetric parameters increased during the growth spurt and was correlated with age (Vd: $R^2 = 0.49$, $p < 0.001$; Vn: $R^2 = 0.33$, $p = 0.02$). However, disc hydration content remained constant at an average 28 %. In the scoliotic group, preoperative volumetric parameters were inferior to the normal values. Hydration content was on average 24 %. At latest follow up, Vd remained stable while Vn increased significantly. Disc hydration content was enhanced to a mean value of 32 %, exceeding the normal values.

*Conclusions:* Comparison between normal and scoliotic discs showed a significant reduction of hydration content in the lumbar spine. 5 year MRI data showed that surgical correction improved the disc hydration properties in the free motion segment. Our results suggest that scoliosis surgery performed at the end of growth can reverse the discs constraints related to spinal deformity. However AIS discs became "overhydrated" compared to controls. This may not be only a return to equilibrium but probably a consequence of excessive stress imposed by the spinal fusion on the unfused lumbar spine. Continuous follow up is required to determine whether surgery in AIS is protective against early degenerative changes.

**April 17**
Scientific session: Spine Part I
08:52–09:45
AUDITORIUM
**OP49**/09:04–09:10

## Static and dynamic evaluation of idiopathic scoliosis in the post-operative period

Eva Polirsztok[1], Vincent Pomero[2], Sebastien Pesenti[2], Guillaume Authier[2], Kim Bin[3], Elke Viehweger[2], Jean-Luc Jouve[2]

[1]Children's hospital, Nancy, France
[2]La Timone Enfants, Marseille, France
[3]La TImone Enfants, Marseille, France

**LEVEL-I**

*Introduction:* One of the goals of the idiopathic scoliosis surgery is to restore a satisfactory vertebral sagittal balance. The post-operative improvement of the dynamic balance is demonstrated by the standard X-Ray. However, the impact on the dynamic balance and the economic biomechanics result are not described in the literature. We propose a static and dynamic study of the spine in the pre and post-operative period. A study of the external efforts upon the lumbo-sacral junction is carried out.
*Material and methods*: This is a monocentric study. Our series includes 13 patients with idiopathic scoliosis. All of them were operated by the same surgeon. They were between 13 and 19 years old. Patients were recorded before and 6 months after the surgical procedure by a motion analysis (Vicon ®) equipped with force plates system. Our protocol calculates the mechanical external forces at the lumbo-sacral junction. Static analysis and the gait and trunk motion were analysed. The pre- and postoperative results were compared. Student T test or Wilcoxon statistical tests were used to compare the pre and post-operative analysis.
*Results:* According to the static analysis, there is a significative difference between both groups for the pelvic obliquity, the hip position, and the posture of the trunk on the pelvis. As for the kinematics analysis, there is a significative difference between both groups for the same parameters throughout the whole gait cycle. The kinetic analysis of external moments highlights a significant decrease of the constraints applied to the lumbo-sacral junction.
*Conclusion*: The movement analysis allows new reproducible parameters for the evaluation of the results in spine deviation surgery to be established.

**April 17**
Scientific session: Spine Part I
08:52–09:45
AUDITORIUM
**OP50**/09:18–09:24

## A comparison of cobalt chromium and titanium alloy rods in adolescent idiopathic scoliosis correction

Emmanuelle Ferrero[1], Brice Ilharreborde[1], David Sabbah[1], Keyvan Mazda[1]

[1]Robert Debre Hospital, Paris, France

**LEVEL-IV**

*Introduction:* Adolescent idiopathic scoliosis (AIS) is a tridimensional deformity (3D). The new generation of constructs and correction techniques such as posteromedial translation enables adequate 3D correction of the deformity. These methods traditionally utilized rods that consisted of stainless steel or titanium alloy (Ti). However, Cobalt chromium (CoCh) rods have been recently gaining in utilization and prior studies have compared artifact incidence, in vitro properties and correctional forces between Ti and CoCh rods. However, postoperative curve correction and potential loss of correction in AIS patients has not been investigated. The purpose of this study is to compare postoperative correction in AIS patients and the change in correction at follow-up between Ti and CoCh rods.
*Methods:* In this retrospective single center study, 60 AIS patients were included. Patients were divided in two groups according to the Ti or CoCh rods (30 patients in each group). All the patients were treated by a single posterior procedure using posteromedial translation with hybrid constructs. Demographic data were recorded. The main sagittal and coronal radiographic parameters were measured preoperatively, postoperatively, and at 1 year follow-up. Groups were compared in terms of demographic and radiographic data at different follow-up times. The change in radiographic parameters between postoperative and 1 year follow-up was measured.
*Results:* Groups were comparable in terms of mean age (17.3 years). Lenke 1 and Lenke 2 Curves were the most common types. Preoperatively, radiographic parameters were not significantly different (mean Cobb angle: $54.4° \pm 12.2$). Postoperative thoracic and lumbar Cobb angle were significantly smaller in the CoCh group than in the Ti group. The correction of main thoracic curve was greater in the Ti group ($-40.1° \pm 11.3$ vs. $-28.3° \pm 17.8$, $p = 0.004$). Similar results were observed at 1 year follow-up. Regarding the change in correction between postoperative and 1 year, there was no significant differences between the groups in terms of coronal or sagittal radiographic parameters.
*Conclusion:* This study compared Titanium and Cobalt Chromium rods used for the correction in AIS patients. Correction of the main thoracic Cobb angle is significantly better with the CoCh rods. These results are concordant with in vitro literature findings demonstrating better biomechiancal corrective forces with CoCh. However, these studies also observed greater plastic deformity for CoCh and whether this finding is seen clinically in vivo requires long term follow-up which can be the basis for a future inquiry.

**April 17**
Scientific session: Spine Part I
08:52–09:45
AUDITORIUM
**OP51**/09:24–09:30

## superior extension of upper instrumented level in distraction based surgery: a surrogate for clinically significant proximal junctional kyphosis

Ron El-Hawary[1], John Heflin[2], Nadim Joukhaider[3], Mohamad Yasin[1], Ozren Kubat[4], David Skaggs[5]

[1]IWK Health Centre, Halifax, Canada
[2]University Hospital Utah, Salt Lake City, United States
[3]Dalhousie Faculty of Medicine, Halifax, Canada
[4]Clinical HospitalCentre Zagreb, Zagreb, Croatia
[5]Children's Orthopedic Centre, Los Angeles, United States

**LEVEL-III**

*Aim:* Proximal Junctional Kyphosis (PJK) is a reported complication of distraction-based treatment for Early Onset Scoliosis (EOS). PJK can be detected radiographically; however, the clinical implications of PJK have not previously been studied for this population. A potential clinically significant consequence of PJK is revision surgery with superior extension of the upper instrument level (UIL). The purpose of this study was to determine the rate of clinically significant PJK during rib-based distraction surgery. A secondary goal was to define the proximal junctional angle (PJA) at the time of revision surgery, with the hypothesis that PJA will be increased in this group of patients.
*Materials and methods:* This is a retrospective review of a multicenter registry for children with EOS. All children treated with rib-based distraction surgery, with minimum 2 year follow up, were evaluated in order to identify the rate of clinically significant PJK (i.e. children who required a revision surgery that involved superior extension of the UIL). Two definitions of PJA were used (PJA-A: angle between the caudal endplate of the Upper Instrumented Vertebrae (UIV) to the cephalad endplate 2 vertebrae above UIV; PJA-B: 2 levels below UIV to 2 levels above UIV).
*Results:* 397 children were identified. At time of implantation, these children had a mean age of 5.5 years, mean scoliosis angle of 69.9°, and mean kyphosis of 49.8°. Forty of these children required revision surgery that involved superior extension of the UIL (10.1 % rate of clinically significant PJK). Despite being younger (4.9 vs 5.5 years, $p < 0.05$), the revision group had similar pre-implantation characteristics as the entire study population, with mean scoliosis of 70.0° and mean kyphosis of 50.0°. Average time to revision was 2.3 years with mean scoliosis of 66.7° and mean kyphosis of 54.7° at time of revision. PJA-A was 5.6° pre-op vs 11.8° at time of revision ($p < 0.05$). PJA-B was 13.1° pre-op vs 21.4° at time of revision ($p = 0.07$).
*Conclusions:* A 10 % rate of clinically significant PJK was found within this group of children who were treated with rib-based distraction surgery. At the time of revision surgery, PJA-A had increased significantly from pre-operative values.

**April 17**
Scientific session: Spine Part I
08:52–09:45
AUDITORIUM
**OP52**/09:30–09:36

## Comparison of all pedicle screw construct and hybrid construct in thoracic idiopathic scoliosis of the adolescent*

Kim Bin[1], Jean Luc Jouve[1], Sébastien Pesenti[1], Emilie Peltier[1], Jean Sébastien Steffen[2], Jean Marc Vital[2], Ibrahim Obeid[2]

[1]Hôpital Timone Enfant, Marseille, France
[2]Hôpital Pellegrin, Bordeaux, France

**LEVEL-IV**

*Introduction:* Many instrumentations have been developed during the last decade to obtain a better correction in the three plains frontal, sagittal and axial, and a better primary stability. Two types of instrumentation were retained in this study: an all pedicle screw construct and a hybrid construct with lumbar pedicle screws, proximal hooks and sublaminar bands. In this study, we focused on a 3D analysis of the spinal parameters and compare the results of the two techniques.
*Materials and methods:* 54 idiopathic thoracic scoliosis were included retrospectively. Inclusion criteria were adolescent idiopathic scoliosis Lenke type 1 or 3, operated by a single posterior approach, in two different centers. 29 patients were instrumented with all pedicle screw construct (PS group) and 25 patients with hybrid construct (H group). A radiographic evaluation was performed; preoperative and postoperative 3D reconstructions with the EOS system were made. The preoperative and postoperative parameters of the 2 groups were compared using a Student paired T-test ($\alpha = 0.05$).
*Results:* The mean age of the patients was 15.7 years old (13–21 years). The Cobb angle decreased from 60° ± 15.8° to 23.8° ± 9.2° in the PS group, and from 47.8° ± 12° to 21.4° ± 16.2° in the H group. Thoracic kyphosis increased significantly in the H group from 22.1° ± 15.9° to 32° ± 9.7°. A significant diminution of lumbar lordosis was observed in the PS group with 59.8° ± 11.6° preoperatively to 52.6° ± 9.2° in postoperatively. Apical vertebral rotation decreased significantly in the 2 groups: from 16.8° ± 6.7° to 7.8° ± 6.7° for the PS group, and from 19.3° ± 7.2° to 13.8° ± 7.1° in the H group. 3 patients had a complementary thoracoplasty in the H group, and no thoracoplasty was performed in the PS group. No neurological or vascular complication was observed in the two groups.
*Discussion:* EOS system is an important tool to analyze 3D spinal parameters. It allows vertebral rotation analysis, especially in the apex, with minimal irradiation. In case of hypokyphosis, hybrid construct seems to restore the sagittal balance significantly. The derotation effect is equivalent in the 2 techniques.
*Conclusion:* We studied the advantages and disadvantages of each instrumentation. Better correction in frontal and axial plane is observed with all pedicle screw construct, and better correction in sagittal plane with hybrid construct.

April 17
Scientific session: Spine Part II
10:15–11:10
AUDITORIUM
**OP53**/10:15–10:21

### Does the "Law of Diminishing Returns" apply to guided growth Shilla constructs?

Lindsay Andras[1], Haleh Badkoobehi[1], Alexander Broom[1], Lynn McCullough[2], Richard McCarthy[2], David Skaggs[1], Growing Spine Study Group[3]

[1]Childrens Hospital Los Angeles, Los Angeles, United States
[2]Arkansas Children's Hospital, Little Rock, United States
[3]Growing Spine Foundation, Milwaukee, United States

**LEVEL-IV**

*Purpose*: In early onset scoliosis (EOS) patients treated with growing rods, Sankar et al. demonstrated progressively less improvement of T1–S1 length over time. This is known as the "law of diminishing returns." Our objective was to determine whether the "law of diminishing returns" applies to EOS patients treated with guided growth Shilla constructs.
*Methods:* Patients with EOS treated with a Shilla construct at two centers were retrospectively reviewed. We included patients who were less than 10 years of age at the time of instrumentation and were followed for a minimum of 2 years. T1–S1 length was measured on upright radiographs preoperatively, postoperatively, and at annual intervals. Non-parametric analysis of variance and linear regression of the data was performed.
*Results:* 33 patients met the inclusion criteria. The mean age at index instrumentation was 6.0 years (range 2.0–9.9 years). The mean follow-up was 4.8 years (range 2–8 years). Postoperative radiographs demonstrated an average gain of 4.7 cm in T1–S1 length from preoperative values. At year 1, however, 73 % (24/33) showed a decrease in T1–S1 length when compared to postoperative T1–S1 lengths. On average the T1–S1 length at 1 year was 1.4 cm less than the postoperative values. There was an overall increase in T1–S1 length in years 2–8 (year 2 = 1.3 cm, 3 = 0.72 cm, 4 = 0.89 cm, 5 = 1.22 cm, 6 = 0.83 cm, 7 = 0.83 cm, 8 = 4.4 cm). The increase in T1–S1 was statistically significant for years 2–5 ($p = 0.003$, $p = 0.02$, $p = 0.006$, $p = 0.005$). There was a similar increase trend toward significance in years 6–8, but it was limited by the small number of patients with greater than 6 year follow-up. The change in T1–S1 between year 1 (a decrease) and 2 was significantly different ($p = 0.0003$). There was no significant difference in the change in T1–S1 over time for the subsequent years ($p = 0.21$, 0.79, 0.78, 0.31, 0.91, 0.27).
*Conclusion:* With a mean follow-up of 5 years, statistically significant annual increases in T1–S1 length were sustained in patients treated with Shilla constructs.
*Significance:* The law of diminishing returns observed in growing rods does not appear to affect Shilla constructs in the same manner.

April 17
Scientific session: Spine Part II
10:15–11:10
AUDITORIUM
**OP54**/10:21–10:27

### How does patient radiation exposure compare with low dose o-arm vs. fluoroscopy for pedicle screw placement in idiopathic scoliosis?

Anthony Stans[1], Alvin Su[1], Amy McIntosh[2], A Noelle Larson[1]

[1]Mayo Clinic, Rochester, United States
[2]Texas Scottis Rite, Dallas, United States

**LEVEL-III**

*Purpose*: CT-guided navigation and intraoperative CT have grown in popularity, but concerns remain regarding the radiation exposure and potential increased cancer risk for paediatric patients. We sought to compare the radiation dose for fluoroscopy vs. intraoperative CT for placement of pedicle screws at two high volume pediatric centers.
*Methods:* This is a retrospective matched cohort study. At one center, pedicle screws for idiopathic scoliosis are typically placed using intraoperative fluoroscopy. At the 2nd center, O-arm (Medtronic, Minneapolis, MN) is frequently used for intraoperative navigation using a low dose paediatric setting (80 kV, 20 mA, 80 mAs as by described by Abul Kasim). These are much lower than the manufacturer-recommended settings. Patients undergoing spinal fusion with either O-arm or fluoroscopy were matched for diagnosis, weight, and length of fusion. Fourteen matched pairs of patients were compared, and the estimated effective dose was computed for each surgery. For reference, annual background radiation is 3 mSv, and the effective dose of a two-view intraoperative scoliosis radiograph is approximately 0.8 mSv.
*Results:* Mean number of levels fused in the O-arm group was 12 vs. 10 levels in the fluoroscopy group ($p = 0.13$). In the O-arm group, there was a mean of 2 scans (range 1–3). Mean seconds of fluoroscopy was 35 (range 8–75), with mean estimated effective dose for the fluoroscopy of 0.27 mSv (range 0.06–0.58). Mean estimated effective dose for the O-arm group was 1.3 mSv (range 0.65–1.95 mSv, $p < 0.001$). Considering the cases with more aggressive fluoroscopy use (>30 s), the estimated effective dose for the patient was still 1/3 of that of O-arm cases (0.43 vs. 1.2 mSv) and comparable to a single low-dose O-arm scan (0.65 mSv).
*Conclusions:* Radiation dosing is higher with O-arm than with fluoroscopy, although prolonged fluoroscopy use (greater than 1 min) can reach levels of exposure equivalent to a single low dose O-arm spin. Radiation imparted for both was similar to that of a standard PA and lateral full-length scoliosis films.
*Significance:* Fluoroscopy and low-dose O-arm are both reasonable means to assist in screw placement for AIS surgery. Avoid using the manufacturer O-arm settings and minimize the number of full-length radiographs for the greatest reduction in patient radiation dose over the entire course of treatment.

April 17
Scientific session: Spine Part II
10:15–11:10
AUDITORIUM
**OP55**/10:27–10:33

### Rib hump pathoanatomy and rib symmetry in adolescent idiopathic scoliosis

Robert M. Campbell[1], Sriram Balasubramanian[2], James Peters[2]

[1]Children's Hospital of Phila., Philadelphia, United States
[2]Drexel University, Philadelphia, United States

**LEVEL-III**

*Purpose*: The rib hump in idiopathic adolescent scoliosis (AIS) is assumed to be an acute posterior angulation rib deformity on the convex side of the curve due to apical vertebral rotation. Asymmetric rib growth has also been proposed as potential cause of scoliosis. Neither assumption has been subjected to rigorous study. Our hypothesis is that the ribs in AIS are normal, symmetrical in shape and length. The objective of this retrospective study is to characterize the rib cage anatomy in AIS subjects and compare with age/gender-matched normative subjects, using CT scans, to assess bilateral rib symmetry.
*Methods:* CT scans: 13 AIS subjects (10 female/3 male, mean age 14.15 ± 1.41 years, mean Cobb angle: 54.38 ± 16.16°) and 13 age/gender-matched normal subjects (10 female/3 male, mean age 14.21 ± 1.53 years). Geometric and positional characteristics of the ribs were determined by 3D reconstructions with Mimics image analysis software (Materialise Inc., Belgium), and rib length, rib apparent curvature along each 10 % increment of rib length, rib enclosed area, lateral angle and frontal angle were computed by a custom MATLAB (The MathWorks Inc., Natick, MA) code. SPSS software (IBM, Armonk, NY) was used to perform non-parametric matched pair t-tests, comparing left and right ribs across appropriate groups for interpretation of bilateral rib symmetry trends.
*Results:* Within the AIS and normative groups, no significant bilateral differences were observed in rib length, rib apparent curvature and rib enclosed area. For only the AIS group, positional differences between convex and concave ribs were found in lateral and frontal angles at T8-T11, roughly at the apex of the lateral curve ($p < 0.05$). For all thoracic levels, no significant differences in rib length, rib apparent curvature and rib enclosed area were observed between the AIS and normative groups.
*Conclusion:* No bilateral rib pair differences were observed in rib length, enclosed area and normalized length within- and between AIS and normative subjects.
*Significance:* Our results support the hypothesis that the rib hump deformity observed in AIS is not an acute posterior rib angulation, but more likely a costo-vertebral downward subluxation of the ribs, and differing rib lengths do not contribute to AIS. Since classic thoracoplasties do not address the true pathoanatomy of the rib hump in AIS, new procedures should be developed to correct the downward costovertebral subluxation that is the anatomic basis of the rib hump.

**April 17**
Scientific session: Spine Part II
10:15–11:10
AUDITORIUM
**OP56**/10:41–10:47

## The lifetime risk of pneumonia in patients with neuromuscular scoliosis at a mean of 21 years age: the role of spinal deformity surgery

Heli Keskinen[1], Heikki Lukkarinen[1], Katariina Korhonen[2], Tuomas Jalanko[2], Ilkka Helenius[1]

[1]Turku University Hospital, Turku, Finland
[2]Helsinki University Hospital, Helsinki, Finland

**LEVEL-III**

*Background/purpose*: Patients with neuromuscular disorders often have increased risk of pneumonia and decreased lung function, which may further be compromised by scoliosis. Scoliosis surgery may improve pulmonary function in otherwise healthy patients, but no study has evaluated its effect on the risk of pneumonia in patients with neuromuscular scoliosis (NMS).
*Methods:* The patient charts of forty-two patients (mean age 14.6 years) operated for severe NMS (mean scoliosis 86° before surgery and mean 32° at the final follow-up.) were retrospectively reviewed from birth to mean 6.1 years (range 2.8–9.5 years) after scoliosis surgery. The main outcome was a radiographically looked for was pneumonia as a primary cause for hospitalization.
*Results:* The lifetime annual incidence of pneumonia was 8.0/100 before and 13.4/100 after scoliosis surgery ($p > 0.10$). Mean hospitalization days per year due to pneumonia were 0.59 (SD 2.3) before scoliosis surgery and 2.24 (SD 6.9) after surgery ($p > 0.10$). Multivariate analysis demonstrated that the lifetime risk factors for pneumonia at mean 21 years of age were epilepsy (RR 15.2, 95 % CI 1.3–176.8, $p = 0.027$), non-CP aetiology (RR = 10.2, 95 % CI 3.2–32.7, $p < 0.001$) and a major scoliosis (main curve >70°; RR = 11.3, 95 % CI 1.8–70.7, $p = 0.01$). Analysis of the risk factors for pneumonia before and after the scoliosis surgery resulted in similar risk factor characteristics as for whole follow-up time. To evaluate further the effect of spinal deformity surgery the risk factors for pneumonia 3 years before and after surgery were examined. During the three-year period before and after surgery the annual incidences of pneumonia were 4.0/100 before and 14.3/100 after surgery ($p > 0.10$). Similarly, before and after operation the hospitalization days in a year because of pneumonia were 0.44 (SD 2.0) and 2.56 (SD 10.7), respectively ($p > 0.10$). According to univariable analysis, the significant risk factors for pneumonia during the 3 years follow-up prior to the scoliosis operation were epilepsy and preoperative main curve >70°. 3 years after the scoliosis operation the risk factors were epilepsy (RR 21.8, 95 % CI 2.9–163, $p = 0.003$), non-CP aetiology (RR 12.2, 95 % CI 1.6–91.7, $p = 0.015$), and preoperative main curve >70° (RR > 100, $p < 0.001$).
*Conclusions:* Epilepsy, non-CP aetiology and a major scoliosis are significant risk factors for pneumonia in patients with NMS. Scoliosis surgery does not decrease the incidence of pneumonia in patients with NMS.

**April 17**
Scientific session: Spine Part II
10:15–11:10
AUDITORIUM
**OP57**/10:47–10:53

## An updated algorithm for radiographic screening of upper cervical instability in patients with down syndrome

Maryse Bouchard[1], Walter Krengel[2]

[1]Seattle Children's Hospital, Seattle, Washington, United States
[2]Seattle Children's Hospital, Seattle, WA, United States

**LEVEL-IV**

*Background:* There is scant evidence in the literature regarding the best approach for radiographic screening of upper cervical instability in patients with Down syndrome. There is also controversy as to what a "normal" radiographic measurement should be recognised in this population, as many have slightly "abnormal" findings without evidence of myelopathy or instability. The purposes of this paper are to determine the best radiographic studies for screening of upper cervical instability in patients with Down syndrome; and to establish normal radiographic measurements in this population.
*Methods:* This study is a retrospective evaluation of 187 lateral cervical spine radiographs in children with Down syndrome that compares the effectiveness of a neutral lateral upright x-ray (NUL) to a full series of cervical spine views in identifying patients at risk of upper cervical instability. On neutral upright lateral x-rays, we measured the atlanto-dental interval (ADI), space available for cord at C1 (SAC), and basion axial interval (BAI). On flexion/extension lateral x-rays, the same measurements were performed on all images, as well as the Wiesel-Rothman (WR) measure.
*Results:* SAC measurements obtained in NUL, flexion and extension films were not significantly different (NUL vs flexion, $p = 0.900$; NUL vs extension $p = 1.000$). ADI measurements were not significantly different between NUL and flexion films ($p = 0.501$). ADI was significantly different between NUL and extension films ($p = 0.000$), however the difference mean ADIs between the two views is not clinically significant (3.13 ± 3.03 mm in neutral and 2.33 ± 2.45 mm in extension). Normal ranges for measurements (mean ± 2 SD) were 0–6 mm for ADI, 14–24 mm for SAC, −12 to 5 mm for BAI, and <7 mm for WR.
*Conclusion:* Our data suggest that obtaining a neutral upright lateral cervical spine x-ray is the most effective method of performing radiographic screening, and we provide "normal" values for the common radiographic measurements used in assessing risk of atlanto-axial and atlanto-occipital instability in patients with Down syndrome.

**April 17**
Scientific session: Spine Part II
10:15–11:10
AUDITORIUM
**OP58**/10:53–10:59

### Chance fractures in the pediatric population are often misdiagnosed*

Lindsay Andras[1], Kira Skaggs[1], Haleh Badkoobehi[1], Paul Choi[1], David Skaggs[1]

[1]Childrens Hospital Los Angeles, Los Angeles, United States

**LEVEL-IV**

*Purpose*: We have encountered several pediatric patients for whom the diagnosis of a Chance fracture was delayed or missed. Our objective was to determine the frequency with which these injuries are misdiagnosed and what characteristics could aid in differentiating Chance fractures from the more benign compression fractures.
*Methods:* A retrospective review was conducted of all patients treated over a 5 year period (2008–2013) for a Chance or compression fracture of the thoracolumbar spine at our pediatric hospital. Patients were excluded if they had prior spine instrumentation or had a pathologic fracture.
*Results:* 88 patients were treated at our institution for spine fractures during the study period. Of the patients who met the inclusion criteria, 26 % (7/27 patients) had a Chance fracture and 74 % (20/27) had compression fracture(s). The mean age of the 7 patients with Chance fractures was 13 years. 71 %(5/7) of the patients with Chance fractures were initially misdiagnosed: (3 as compression fractures, 1 as a burst fracture, 1 as muscular pain) and 80 % (4/5) of these misdiagnoses were made by a neurosurgeon or orthopedic surgeon. Average delay in diagnosis was 95 days (range 2–311) with 57 % (4/7) of the patients having ≥1 month delay. Of the 7 patients, 1 was treated initially at our institution, 2 were transferred from outside hospitals and 4/7 (57 %) were seen in our clinic for chronic back pain. One patient was correctly diagnosed with a ligamentous Chance fracture but initially mistreated in a brace prior to presentation. None of the patients with Chance fractures had a neurologic injury. Six patients with posterior ligamentous disruption were treated with surgical instrumentation and fusion. All injuries occurred between the levels of T12 and L3.
*Conclusion:* The majority of pediatric Chance fractures in this series were initially misdiagnosed (71 %; 5/7) or mistreated (14 %;1/7) by neurosurgeons or orthopaedic surgeons. Average time to the correct diagnosis was 3 months for the Chance fractures in this series.
*Significance:* Paediatric Chance fractures generally require surgery, and pediatric compression fractures are generally treated non-operatively, so distinguishing between these two is critical. While compression fractures are 3 times more likely than Chance fractures, attention to the posterior third of the spine on physical exam and imaging is warranted to avoid missing a Chance fracture.

**April 17**
Scientific session: Other/varia
11:10–12:10
AUDITORIUM
**OP59**/11:10–11:16

### Femoral and tibial torsion measurements in children and adolescents: comparison of MRI and 3D models based on low-dose biplanar radiographs

Leonhard Ramseier[1], Andrea Rosskopf[2], Florian Buck[2]

[1]Orthopedic Department, Zurich, Switzerland
[2]Radiologic Department Balgrist, Zurich, Switzerland

**LEVEL-III**

*Purpose*: Femoral and tibial torsion measurements have been shown to be comparable to state of the art CT measurements. However, CT measurements as well as measurements on 3D models based on low-dose biplanar radiographs are associated with radiation exposure of the child. The aim of our study was to evaluate reliability and interchangeability of femoral (FT) and tibial torsion (TT) measurements in children using magnetic resonance (MR) imaging compared to measurements on 3D models based on biplanar radiographs (BPR).
*Materials and methods*: Institutional review board waiver was obtained. FT and TT were measured in 30 children (mean age 10.1 years, range 6.2–15.6 years; 14 female) using axial MR imaging scouts by two independent readers. Measurements based on BPR 3D models by two separate independent readers served as reference standard. Inter-reader and inter-method agreement were calculated using descriptive statistics, Intraclass correlation coefficient (ICC) and Bland–Altman analysis.
*Results:* FT/TT was −6° to 47°/+1° to 44° on MR images and −13° to 46°/9°–49° for measurements on BPR 3D models. The average difference between the two methods was 4.6° ± 4.1/6.0° ± 3.8, respectively. Inter-reader agreement (ICC) of FT/TT measurements was 0.97/0.96 on MR images and 0.99/0.94 on BPR 3D models. Inter-method agreement (ICC) for MR measurements was 0.93 [95 % confidence interval (CI) 0.88–0.96] for FT and of 0.87 (CI 0.39–0.95) for TT. Mean measurement differences between the two BPR readers were 2.1° (0.0°–7.0°) for FT and 3.4° (0.0°–12.0°) for TT. Mean inter-reader differences at MR were 3.2° (0.1°–8.0°) for FT and 3.5° (0.1°–9.5°) for TT. Bland–Altman plots showed no relevant differences between the two modalities. Except for 3/4 of FT/TT measurements, all measurements on MR images were within the 95 % limit of agreement.
*Conclusion:* FT and TT measurements in children using MR scout imagesare are comparable to measurements on BPR 3D models.

**April 17**
Scientific session: Other/varia
11:10–12:10
AUDITORIUM
**OP60**/11:16–11:22

## Comparison of the simplified olecranon and digital methods of assessment of skeletal maturity during the pubertal growth spurt

Federico CANAVESE[1], Philippe CHARLES[2], Alain DIMEGLIO[3], Marie ROUSSET[1], Sebastien SCHULLER[2], Antoine SAMBA[1], Bruno PEREIRA[4], Jean-Paul STEIB[2]

[1]University Hospital Estaing, Clermont Ferrand, France
[2]CHU Strasbourg, Strasbourg, France
[3]University of Montpellier, Montpellier, France
[4]CHU Clermont-Ferrand, DRCI, Clermont Ferrand, France

**LEVEL-III**

*Purpose*: Both Dimeglio et al's and Sanders et al's simplified methods of assessing skeletal age have been reported to have a modest learning curve and ease of clinical application. This study compares the accuracy of both methods during the pubertal growth spurt and investigates the accuracy in relation to the experience of the assessor.
*Methods:* Our investigation was based on the antero-posterior left hand and lateral elbow radiographs of 44 boys (mean 14.4, range 12.4–16.1) and 78 girls (mean 13.0, range 11.1–14.9) obtained during the pubertal growth spurt. A total of nine observers examined the radiographs with the observers assigned to three groups based on their experience (experienced, intermediate and novice). These raters were required to determined skeletal ages twice at 6 weeks intervals. The correlation between the two methods was determined per assessment and per observer groups. Interclass correlation coefficients (ICC) evaluated the reproducibility of the two methods.
*Results:* A total of 244 radiographs (122 hands and 122 elbows) were reviewed twice by each of the nine observers, for a total of 4392 ratings (2196 hands and 2196 elbows).The overall correlation between the two methods was $r = 0.83$ for boys and $r = 0.84$ for girls. The correlation was equal between first and second assessment, and between the observer groups ($r = 0.82$). There was an equally strong ICC for the assessment effect (ICC = 0.4 %) and observer effect (ICC = 3 %) for each method. There was no significant ($p < 0.05$) difference between the levels of experience.
*Conclusion:* The two methods are equally reliable in assessing skeletal maturity. The olecranon method offers detailed information during the pubertal growth spurt while the digital method is as accurate but less detailed, making it more useful after the pubertal growth spurt once the olecranon has ossified.
*Significance:* A combination of both methods adequately covers the gap between elbow fusion and Risser grade 1, as one complements the other.

**April 17**
Scientific session: Other/varia
11:10–12:10
AUDITORIUM
**OP61**/11:22–11:28

## Minimally invasive repair of pectus excavatum using the Nuss technique in children and adolescents: indications, outcomes, and limitations

Naïma LOUCHEUR[1], Reda KADDAJ[2], Marion Brunier[3], Rémi Kohler[3], Rémi Dubois[4], Jean Luc Jouve[5]

[1]Hôpital Timone Enfants, marseille, France
[2]hôpital de Lyon, Lyon, France
[3]Hôpital de lyon, Lyon, France
[4]Hôpital de Lyon, Lyon, France
[5]Hôpital Timone enfants, Marseille, France

**LEVEL-IV**

*Background:* Pectus excavatum (PE) is a common congenital deformity. The Nuss technique for minimally invasive repair of PE involves thoracoscopy-assisted insertion of a bar or plate behind the deformity todisplace the sternum anteriorly. Our objective here was to clarify the indications and limitations of the Nuss technique based on a review of 70 patients.
*Materials and methods:* A retrospective review of children managed at two centres identified 70 patients who had completed their growth and had their plate removed. Mean age was 13.8 years (range 6–19 years). The reason for surgery was cosmetic disfigurement in 66 (95 %) patients. The original Nuss technique was used in 63 patients, whereas 7 patients required an additional sub-xiphoid approach. Time to implant removal ranged from 8 months to 3 years.
*Results:* The cosmetic outcome was considered satisfactory by the patients in 64 (91 %) cases and by the surgeon in 60 (85.7 %) cases. Major complications requiring further surgery occurred in 6 (8.5 %) patients and consisted of haemothorax ($n = 2$), chest wall sepsis ($n = 2$, including 1 after implant removal), allergy ($n = 1$), and implant displacement ($n = 1$). Early or delayed minor complications occurred in 46 (65 %) patients and resolved either spontaneously or after non-surgical therapy.
*Discussion:* The minimal scarring and reliably good outcomes support the widespread use of the Nuss technique in children and adolescents. Our complication rates (minor, 65 %; and major, 8.5 %) are consistent with previous publications. In our opinion, contra-indications to thoracoscopic PE correction consist of a history of cardio-thoracic surgery and the finding by computed tomography of a sternum-to-spine distance of less than 5 cm or of sternum rotation greater than 350. In these situations, we recommend a sub- and retro-xiphoid approach to guide implant insertion or a classic sterno-chondroplasty procedure.

**April 17**
Scientific session: Other/varia
11:10–12:10
AUDITORIUM
**OP62**/11:36–11:42

## Gait pattern and lower extremity alignment in children with diastrophic dysplasia

Ilhan A Bayhan[1], Mehmet S Er[1], Tristan Nishnianidze[1], Colleen Ditro[1], Kenneth J Rogers[1], Freeman Miller[1], William G Mackenzie[1]

[1]NAIDHC, Wilmington DE, United States

**LEVEL-III**

*Purpose*: The aim of this study was to describe the dynamic lower extremity alignment in children with Diastrophic Dysplasia (DD) by three dimensional gait analyses. Our main hypothesis was the gait kinematics and kinetics are different than the age normalized population and patellar dislocation can alter the gait in DD patients.
*Methods:* A retrospective review of clinical data and x-rays was conducted for DD patients who had gait analysis prior to lower extremity skeletal surgery excluding foot procedures. Lower extremity range of motion was measured. The Pediatric Outcomes Data Collection Instrument (PODCI) was administered to parents of patients to evaluate their functional status. Gait laboratory data were collected to compare the hip and knee kinematics in cases with and without patellar dislocation. Antero-posterior standing x-rays were taken for all patients to assess the correlation between measurements (clinical, radiological and gait) of coronal knee alignment.
*Results:* 30 lower extremities of 15 children (7 female and 8 male) were evaluated. The mean age was $7.4 \pm 3$, mean height was $97.7 \pm 15$ cm ($z = -5.1$) and mean weight was $20.6 \pm 6.2$ kg ($z = -0.8$). DD PODCI sub-scores were statistically significantly lower ($p < 0.05$) than the average stature developing children except for the happiness score. Gait Analysis, compared between all DD and an age normalized average stature group showed decreased forward velocity, step length and stride length with increased average forward tilt of the trunk and pelvis, hip flexion, hip adduction and internal rotation ($p < 0.001$). Delta hip motion and delta knee motion were also decreased ($p < 0.001$). The patella was dislocated in 19 (63.3 %) knees and central in 11 knees (36.6 %). Comparison of the minimum knee and hip flexion in the stance phase demonstrated increased crouch gait in the patellar dislocation group ($p < 0.001$). Knee alignment measurements between clinical examination and gait analysis showed moderate correlation (r: 0.476, $p = 0.008$).
*Conclusion:* Children with DD demonstrated lower PODCI sub-scores except for happiness. Gait analysis showed limited lower extremity function of the children with DD. Patella dislocation group had increased crouch gait.
*Significance:* Patella dislocation in the DD population can have a negative impact on gait and function.

**April 17**
Scientific session: Other/varia
11:10–12:10
AUDITORIUM
**OP63**/11:42–11:48

## Traumatic epiphyseal separation in children: statistical validation of the prognostic value of salter-harris classification*

Cosimo Gigante[1], Cristina Formentin[1], Claudio Iacobellis[2]

[1]Pediatric Orthopaedic Unit, Padua, Italy
[2]Orthopaedic Clinic Padua, padua, Italy

**LEVEL-III**

*Introduction:* Epiphyseal traumatic separations may be complicated in children by growth disturbances. The rate of these complications and the prognostic value of the classic Salter-Harris (S–H) classification remain controversial in literature.
*Materials and methods*: We reviewed 172 patients treated for epiphyseal separation fractures in the decade 2003–2013. The lesions were classified according to S–H and the complication rate was established. The statistical correlation between growth disturbances and S–H classification was researched. To improve the power of the statistical analysis this correlation was enlarged to the 469 patients reported by Mizuta (1987) and Kawamoto (2006), who performed their studies according to our criteria except for statistical analysis. Collecting all the patients there were 822 epiphyseal fractures: 172 type I S–H fractures (34,9 %), 535 type II (65 %), 58 Type III (7 %), 55 type IV (6,6 %), 2 type V (2,4 %). The interaction between complications and S–H type was analyzed using the Cochran-Armitage test as multicategorial variables. The evaluation of the prognostic value of the Salter-Harris classification was performed using a univariate linear logistic regression.
*Results:* The complication rate was 1,82 % (15 patients) with the corresponding distribution : 2 growth disturbances in S–H I, 7 in S–H II, 2 in S–H III, 2 in S–H IV, 1 in S–H V. The estimation results to assess whether the Odd Ratio occurrence of complications depended on the classification of Salter-Harris included the database of Mizuta, Kawamoto and the present study. This showed that the effect of the type of epiphyseal fractures on the onset of complications is not statistically significant with regard to the Types I, II, III and IV (using SH type I fracture as reference: type II p value $= 1.0000$, type II p value $= 0.4396$; type III p value $= 0.3539$). The only statistical significance was found for type V lesions (p value $= 0.0005$) but this data hasn't any practical value as the radiographic diagnosis of this lesion may only be done at the onset of the late post traumatic deformity.
*Conclusions:* Growth disturbances following epiphyseal separation in children are quite rare (less than 2 %). The prognostic value, generally attributed to the classic S–H classification, wasn't confirmed on a large series of growing patients as the clinical results, statistically processed at the follow-up, failed to show any significant correlation with the Salter Harris morphological design. On the basis of this study the SH classification has only of descriptive value.

**April 17**
Scientific session: Other/varia
11:10–12:10
AUDITORIUM
**OP64**/11:48–11:54

## Dual-port endoscopic surgery for intraosseous lesions in children

Takashi Saisu[1], Jun Kakizaki[1], Yasuhiro Oikawa[1], Reiko Tanaka[1], Yuko Segawa[2], Mitsuaki Morita[3], Yuta Tsukagoshi[3], Makoto Kamegaya[3]

[1]Chiba Children's Hospital, Chiba, Japan
[2]Tokyo Medical and Dental Univ., Tokyo, Japan
[3]Chiba Child and Adult Orthop., Chiba, Japan

**LEVEL-IV**

*Purpose*: Since 2002, we have treated intraosseous lesions in children using an endoscopic technique with drilled dual opposing insertion ports to manipulate instruments much as we do during arthroscopy and to reduce encroachment into the growth plates during resection of the lesion. The purpose of this study is to verify the efficacy and safety of this procedure.
*Subjects and methods:* From 2002, 24 intraosseous lesions, such as osteomyelitis, physeal bar and tumor in 20 patients were treated with this procedure. Operative site, age at operation, complications in perioperative period, duration of follow-up, recurrence of osteomyelitis and growth disturbance were reviewed retrospectively.
*Results:* The mean age at operations was 8.6 years old (range 1.8–17.3) and mean follow-up was 2.2 years. There were 12 patients with osteomyelitis involving the physis of the distal femur in 2, the proximal tibia in 6, the distal tibia in 1, the distal fibula in 1, the distal radius in 1, and the 1st metatarsal bone in 1. In 10 of the12 cases, osteomyelitis was clinically resolved in a short period after operation and neither growth disturbance nor angular deformity of affected bone was identified in follow up, although additional operations were required due to inappropriate choice of antibiotics or insufficient resection of the sequestrum in the remaining 2 cases. There were 6 patients with a physeal bar of the distal femur in 4, the proximal tibia in 2, and the distal tibia in 1. One patient had endoscopic physeal bar resection for two different lesions, and another patient had endoscopic physeal bar resection after endoscopic surgery for osteomyelitis. Apparent growth recovery was observed in 4 lesions in 3 cases under the age of 7, while growth recovery was insufficient in 3 cases over the age of 9. Two patients with chondroblastoma in the femoral heads and one patients with enchondroma in the 4th middle phalanx, the 4th proximal phalanx, and the 4th and 5th metacarpal bones were successfully treated with this technique. No surgical complication was identified in any cases.
*Conclusion:* Dual-port endoscopic surgery appears to be safe and useful for the treatment of osteomyelitis, physeal bar and intraosseous tumour extending across the physis. There may be a potential advantage to the use of endoscopic techniques in patients with intraosseous lesion in children.

**April 17**
Scientific session: Other/varia
11:10–12:10
AUDITORIUM
**OP65**/11:54–12:00

### Identification of gait pathologies in children with spina bifida and the role of gait analysis

Deirdre Ryan[1], Bitte Healy[1], Sylvia Ounpuu[2], Jeffrey Thomson[2], Nicole Mueske[1], Pauline Yasmeh[1], Tishya Wren[1], Rachel Y. Goldstein[3]

[1]Childrens Hospital Los Angeles, Los Angeles, United States
[2]Connecticut Children's, Hartford, United States
[3]Children's Hospital Los Angeles, Los Angeles, United States

**LEVEL-III**

*Purpose*: The purpose of this study was to characterize gait deviations in children with spina bifida and evaluate the effects of gait analysis on pathology identification.
*Methods:* This study included 44 children with spina bifida (27 males; 11.0 ± 3.7 years) who were previously seen for clinical gait analysis at two paediatric hospitals. Two paediatric orthopaedic surgeons and two therapists (physical therapist or kinesiologist) from the gait labs at different institutions, and who were experienced in the use of gait analysis in this population identified primary gait pathologies based on a retrospective review of videos and physical therapy evaluations alone, and again with the addition of gait analysis data including kinematics and kinetics. Each side was assessed separately for each subject (88 sides). The frequency of pathology identification was compared between video/physical therapy evaluation only versus video/physical therapy evaluation plus gait analysis data using the 2-sided Fisher's exact test.
*Results:* Crouch (41 % for surgeons, 63 % for therapists), tibial rotation (40, 45 %), and pes valgus (24, 40 %) were the pathologies most commonly identified by both surgeons and therapists before gait analysis. Gait analysis increased the identification of crouch among surgeons (41–53 %, $p = 0.03$) and decreased the identification of genu valgum among therapists (36–20 %, $p = 0.001$). Gait analysis also increased the identification of excessive hip flexion (10–44 %, 13–23 %) and abnormal femoral rotation (7–29 %, 16–28 %) for both surgeons and therapists ($p \leq 0.02$).
*Conclusion:* Excessive hip flexion and abnormal hip rotation were often identified only after gait analysis. Hip rotation is difficult to assess due to the complex rotational profiles that may include large transverse plane rotations of the pelvis during a single gait cycle. Moreover, visual assessment of hip flexion is challenging because of the difficulty in appreciating pelvic sagittal plane motion. This may be significant and changing throughout a single gait cycle. Crouch, tibial rotation, and pes valgus could often be recognized based on physical exam and visual assessment, although the assessment of crouch may benefit from gait analysis data.
*Significance*: Gait abnormalities in spina bifida are complex and often interrelated, making it difficult to determine the primary pathologies using only visual assessment. Gait analysis can assist clinicians in accurately and objectively determining primary pathologies which will guide therapeutic and surgical decision-making.

**April 17**
Scientific session: Multicentre + ostegenesis imperfecta + innovative
14:25–15:45
AUDITORIUM
**OP66**/14:25–14:31

### The unsolved problem of relapse after clubfoot treatment by the ponseti method: putting efforts together to find risk factors*

Cristina Alves[1], Christof Radler[2], Anna Ey[3], Dan Cosma[4], Yael Gelfer[5], Deborah Eastwood[5], Gaetano Pagnotta[6], Yoram Hemo[7], Shlomo Wientroub[8], Andreas Roposch[9]

[1]Hospital Pediátrico-CHUC, EPE, Coimbra, Portugal
[2]Speising Orthopedic Hospital, Vienna, Austria
[3]Hospital Sant Joan de Déu, Barcelona, Spain
[4]Cluj-Napoca Hospital, Cluj, Romania
[5]Great Ormond Street Hospital, London, United Kingdom

[6]IGPPPM- Italy, Rome, Italy
[7]Dana Children's Hospital, Tel-Aviv, Israel
[8]Dana Childrens Hospital, Tel-Aviv, Israel
[9]Great Ormond Street Hospital, London, Portugal

**LEVEL-III**

*Purpose*: To acquire epidemiological data regarding the occurrence of relapses and determine risk factors for relapse in children with clubfoot treated in their first year of life by the Ponseti Method.
*Methods*: A case–control study was done to assess the relationship between possible risk factors (number of casts needed to treat, age when treatment started, tenotomy, existence of previous non-Ponseti treatments, noticed difficulties during bracing) and the occurrence of a relapse. Attending physicians from different institutions identified eligible patients and collected demographic and clinical data. 'Cases' were identified as patients with age >5 years old who had a clubfoot initially corrected by Ponseti Method and sustained at least one recurrence, which demanded casting, physiotherapy or surgery. 'Controls' were patients with age >5 years old, who have been treated for clubfoot and had no signs of relapse at the time of the study. Patients with other lower limb abnormality or neuromuscular disease were excluded from this study.
*Results*: 7 institutions identified 359 patients older than 5 years of age who had Ponseti treatment for clubfoot. 159 patients had a relapse and 200 patients had no history of relapse and constituted the 'control-group'. In the 'relapsed-group', 69 % of patients were males and 48.8 % had a bilateral clubfoot; treatment was started at 29 ± 47 days of age; 95.6 % patients had a tenotomy and 30.1 % of patients needed more than 6 casts to achieve correction. In 41 % of cases, difficulties with bracing were reported; relapse was diagnosed at 50 ± 23 months of age; 69.8 % of patients had a tibialis anterior transfer at 67.4 ± 2.9 months of age and 3.1 % had a postero-medial release. In the 'control-group' 71 % of patients were males and 56.4 % had a bilateral clubfoot; treatment was started at 27 ± 45 days of age; 92.5 % patients had a tenotomy and 26.6 % of patients needed more than 6 casts to achieve correction; in 41.8 % of cases, difficulties with bracing were reported.
*Conclusions*: Patients who sustained a relapse were not significantly different from patients who remained with their feet corrected after age 5. The age at which clubfoot relapse was diagnosed has increased when compared to previous studies on this problem.
*Significance*: Recurrence of the deformity after clubfoot treatment by the Ponseti Method remains an unsolved problem and prospective multicenter studies are needed to identify risk factors.

**April 17**
Scientific session: Multicentre + ostegenesis imperfecta + innovative
14:25–15:45
AUDITORIUM
**OP67**/14:31–14:37

## Hemiepiphysiodesis: preliminary results of a multicenter study*

Barry Danino[1], Robert Rödl[2], John E. Herzenberg[3], Lior Shabtai[3], Franz Grill[4], Unni Narayanan[5], Eitan Segev[1], Shlomo Wientroub[1]

[1]Ped. Ortho. Dep. Dana Hospital, Tel Aviv, Israel
[2]Universitätsklinikum Münster, Münster, Germany
[3]Rubin Institute Sinai Hospital, Baltimore, United States
[4]Orthopedic Hospital Speising, Vienna, Austria
[5]Sickkids Hospital, Toronto, Canada

**LEVEL-III**

*Background:* Guided growth by tension band plating is commonly used to correct coronal plane deformity around the knee. We present preliminary results of a multicenter study including more than 500 patients treated with this method. The purpose of this study was to measure the effect of temporary hemiepiphysiodesis and to define further parameters that influence success.
*Method:* This was a retrospective multicenter study conducted at 5 centers including data on 967 physes in 537 patients, with an average follow up of 14–16 months after plate insertion. Alignment analysis was compared preoperatively with at least 2 measurements postoperatively; rate of correction was calculated, univariate and multivariate analysis was performed to determine parameters that influence the rate and amount of correction.
*Results:* The average age at plate implantation was 11.35 years (range 1.33–19.33). 60 % of femoral physes were corrected to standard alignment (mechanical lateral distal femoral angle between 85° and 89°); 14 % have not yet achieved correction, and are still growing, 3 % were not fully corrected despite achieving skeletal maturity, while 22 % have been overcorrected. 60 % of tibia physes were corrected to standard alignment (medial proximal tibial angle between 85° and 89°), 25 % have not yet achieved correction and are still growing, 7 % were not fully corrected despite achieving skeletal maturity, while 8 % have been overcorrected. Rate of correction was 0.76°/month for the right femur, 0.77°/month for the left femur, 0.8°/month for the right tibia, and 0.79°/month for the left tibia. Factors that were found significantly to influence both rate and amount of correction were: etiology, age at plate implantation in the femurs, and the amount of deformity in the tibias.
*Conclusion:* Temporary hemiepiphysiodesis can effectively treat angular deformities. Factors that influence the success of treatment and the rate of correction include the age of the patient at plate implantation, aetiology and amount of deformity.

**April 17**
Scientific session: Multicentre + ostegenesis imperfecta + innovative
14:25–15:45
AUDITORIUM
**OP68**/14:37–14:43

## Re-operation after magnetically controlled growing rod implantation: a review of 26 patients with minimum 2-year follow-up*

Kenneth Cheung[1], Kenny Kwan[1], John Ferguson[2], Colin Nnadi[3], Ahmet Alanay[4], Muharrem Yazici[5], Gokhan Demirkiran[5], Ilkka Helenius[6], Behrooz Akbarnia[7]

[1]Queen Mary Hospital, Hong Kong, China
[2]Starship Children's Hospital, Auckland, New Zealand
[3]Oxford University Hospitals, Oxford, United Kingdom
[4]Acibadem University, Istanbul, Turkey
[5]Hacettepe University, Ankara, Turkey
[6]Turku Children's Hospital, Turku, Finland
[7]SanDiegoCtrForSpinalDisorders, San Diego, United States

**LEVEL-IV**

*Purpose*: Traditional growing rods (TGR) have been widely used for the treatment of early onset scoliosis (EOS). A high complication rate is attributed to frequent surgical lengthening. Magnetically controlled growing rod (MCGR) allows non-invasive distractions in awake patients and is believed to reduce the number of additional surgeries after rod implantation. This study aims to report on the rate and reason for reoperation after MCGR surgery.
*Methods:* Consecutive patients undergoing MCGR treatment with a minimum of 2-year follow-up from 6 centres were included. All clinical and radiographic data were collected prospectively.
*Results:* 26 patients were included in this study. The mean age at the time of surgery was 7.3 years (range 4–14 years) and the mean follow-up period was 35 months (range 24–50). 4 had single-rod and 22 had dual-rods implanted. 11 patients (42.3 %) required reoperation within the follow-up period, with a mean time to reoperation of 17 months after initial surgery (range 5–29 months). Five were due to failure of rod distractions and the rods were all changed except in one case, when 1 of the 2 rods was removed; 3 rods were due to failure of proximal fixation of the implant; 2 were due to rod breakage; and there was one case of wound infection with failure of proximal fixation. Comparing the group that needed reoperation to those that did not, there was no relationship between reoperation and preoperative Cobb angle, age at surgery, level of instrumentation, number of distraction episodes or cases that were converted from TGR.
*Conclusion*: This is the largest series with the longest follow-up to date that examines the need for additional surgery after the initial procedure. MCGR surgery is associated with a high frequency of reoperations for a variety of reasons. However, it compares well with TGR patients, as all will require reoperation within the first year for lengthening. Longer-term studies and comparisons with TGR patients are required to evaluate the efficacy and cost-effectiveness of this new implant for the management of patients with Early Onset Scoliosis.
*Significance*: MCGR surgery for the treatment of early onset scoliosis is associated with a high frequency of reoperations for a variety of reasons. However, it compares well with traditional growing rods, as all will require reoperation within the first year for lengthening.

**April 17**
Scientific session: Multicentre + ostegenesis imperfecta + innovative
14:25–15:45
AUDITORIUM
**OP69**/14:43–14:51

### Rigid fixation improves outcomes of spinal fusion for C1–C2 instability in children with skeletal dysplasias

Ilkka Helenius[1], Haemish Crawford[2], Paul Sponseller[3], Thierry Odent[4], Robert Bernstein[5], Anthony Stans[6], Daniel Hedequist[7], Jonathan Phillips[8]

[1]Turku University Hospital, Turku, Finland
[2]Starship Children's Hospital, Auckland, New Zealand[3]Johns Hopkins University, Baltimore, United States
[4]Hôpital Necker-Enfants-Malades, Paris, France
[5]Cedars-Sinai Medical Center, Los Angeles, United States
[6]Mayo Clinic, Rochester, United States; [7]Children's Hospital of Boston, Boston, United States
[8]Arnold Palmer Hospital, Orlando, United States

**LEVEL-III**

*Aim:* To compare the outcomes of upper cervical spinal fusion for C1–C2 instability in children with skeletal dysplasias using rigid and non-rigid fixation.
*Materials and methods:* A retrospective, multicenter study identified 28 patients (mean age at surgery 6.7 years; mean FU 5.6 years) operated for C1/C2 instability (mean BL 130 mL; OR time 3.4 h) with skeletal dysplasias. between 2000 and 2011 with minimum 2-year FU. The diagnosis was Morquio 9, SED 9, Kniest 2, SEMD 2, metatropic dysplasia 2, metaepiphyseal chondrodysplasia 1, Hunter disease 1, Maroteau-Lamy 1, chondrodysplasia punctata 1. 14 patients underwent either non-instrumented or instrumentation with wires/cables (group 1) and 14 patients with screws/hooks and rods (group 2). All patients received autograft, 22 were immobilized with halo-body jacket (mean 3.0 mths; 12/14 in group 1 and 10/14 in group 2).
*Results:* The leading reasons for C1/C2 instability diagnosis was screening (12 pts), neurologic deficit (7 pts), and neck pain (8 pts). Mean AAD in extension and flexion was 7.8 mm, SAC at C1/C2 8.8 mm, and 10pts showed a high signal area of SC in preop MR. 7 patients(29 %) showed a neurological deficit preop (mean preop JOA UE score 2.3; LE score 2.1). 26 (93 %) patients had a dysmorphic dens. 14 pts underwent C1/C2 fusion and 14 pts occipito-cervical fusion. 12 patients (43 %) had spinal cord decompression. 6 patients (21 %) needed revision surgery for pseudoarthrosis. The risk of pseudoarthrosis was significantly higher in group 1 (6/14) than in group 2 (0/14; $p = 0.0057$). No new neurological deficits were observed and 5/7 patients with neurological deficit preop showed partial recovery (mean JOA UE score 3.0; LE score 2.6 at FFU, $p = 0.047$). CSF leaks and vertebral artery tears during C2 pedicle screw insertion each occurred in two patients.
*Conclusion:* The risk of pseudoarthrosis is relatively high in patients undergoing C1/C2 spinal fusion with non-instrumented or wire/cable instrumentation, even with halo-vest. Rigid fixation with screws and rods seems to be beneficial although on occasions technically demanding. A dysmorphic dens is a typical finding in these patients needing C1/C2 instrumented spinal fusion.
*Significance:* Rigid fixation improves outcomes of instrumented fusion for C1/C2 instability in children with skeletal dysplasias.

**April 17**
Scientific session: Multicentre + ostegenesis imperfecta + innovative
14:25–15:45
AUDITORIUM
**OP70**/14:57–15:03

### Long-term functional outcome of forearm rodding in children with osteogenesis imperfecta

Elizabeth Ashby[1], Kathleen Montpetit[1], Reggie C. Hamdy[1], Francois Fassier[1]

[1]Shriners Hospital, Montreal, Canada

**LEVEL-IV**

*Purpose*: To assess the long-term impact of corrective forearm surgery on functional ability, ambulation, grip strength, joint range of motion and cosmetic appearance in children with Osteogenesis Imperfecta.
*Materials and methodology*: A retrospective chart review was conducted on 22 children with osteogenesis imperfecta who underwent corrective forearm surgery stabilized with intramedullary rodding at our institution between 1996 and 2013. Functional ability was assessed using the self-care and mobility domains of the Pediatric Evaluation and Disability Inventory (PEDI). Ambulation was assessed using the modified Bleck score. Grip strength was measured using a dynamometer and joint range of motion was measured with a goniometer. Deformity was measured on antero-posterior (AP) and lateral radiographs of the forearm. Outcome measures were assessed pre-operatively and every year post-operatively. Differences between pre-operative and 1-year post-operative outcomes were compared using paired T-tests. All tests were 2-tailed and a p-value of 0.05 was considered significant. In 18 patients with a minimum of 2 years follow-up, outcome measures at 1-year post-surgery were compared to those at the latest clinic visit (mean follow-up time is 8.9 years, range 2.0–16.4 years).
*Results:* Corrective forearm surgery resulted in a significant improvement in PEDI self-care score (mean change = +6.77, p = 0.0017) and mobility score (mean change = +7.21, p = 0.020) at 1 year post-surgery. There was a significant improvement in the Bleck ambulation score (mean change = +0.57, p = 0.007) and grip strength (mean change = +6.13 N, p = 0.015) at 1-year after surgery. Over 80 % of improvements were maintained at a mean of 8.2 years follow-up. There was a significant improvement in the radiographic angular deformity of the radius (mean change on AP view = 39.7° p = <0.0001, mean change on lateral view = 38.2° p = <0.0001) and the ulna (Mean change on AP view = 24.7° p = 0.0016, mean change on lateral view = 27.2° p = <0.0001) following surgery. Elbow and wrist range of motion did not change following surgery. There were post-operative complications: 8 cases with prominent K-wires (all of which required further surgery) and 2 cases of non-union. There was no deep infection, compartment syndrome or physeal growth arrest.
*Conclusions:* Corrective forearm surgery in children with osteogenesis imperfecta leads to long-term improvement in functional ability, mobility, grip strength and cosmetic appearance. Elbow and wrist range of motion do not change.
*Significance:* This is the first case series reporting long-term improvement in function following corrective forearm surgery in children with osteogenesis imperfecta.

**April 17**
Scientific session: Multicentre + ostegenesis imperfecta + innovative
14:25–15:45
AUDITORIUM
**OP71**/15:03–14:43

## Operative treatment of osteogenesis imperfecta: rotationally stable vs unstable telescopic nails

Sergey Khmyzov[1], Konstantin Romanenko[1], Andrey Pashenko[1], Dmytro Iershov[1]

[1]Sytenko Institute, Kharkiv, Ukraine

**LEVEL-IV**

*Introduction:* Initially we designed an intramedullary telescopic nail (ITN) for lower limb deformity correction in patients with osteogenesis imperfecta (OI). It consists of cylindrical rode and tube. ITN provides a splinting effect during longitudinal bone growth but it doesn't provide rotational stability. We developed a rotationally stable intramedullary telescopic nail (RSITN) in 2011.
*Aim:* to compare the preliminary results of ITN vs RSITN implantation for lower limb deformity correction in patients with OI.
*Patients and methods*: A retrospective clinical case study was conducted for patients with OI. Inclusion criteria: patients with multiple fractures and lower limb deformities with open physis who underwent nailing surgery. Twenty five OI patients were treated from 2008 to 2014. These patients were divided in two groups according to the type of implant used in surgery. Group 1–9 patients: 5 males; 4 female. Average age—6.5 year. Surgery: corrective osteotomies and osteosynthesis with ITN. A total of 11 ITNs were implanted (femur–6; tibia—5). Group 2—8 patients: 6 males; 2 females. Average age—4.7 year. Surgery: corrective osteotomies and osteosynthesis with RSITN. 12 RSITNs were implanted (femur—7; tibia—5). In Group 1 the average follow up period was 56 months; in Group 2—22 months. Clinical results, complication rate, preoperative and follow-up radiographs (AP and Lateral) to assess bone alignment and nail elongation were evaluated in both groups. Normal alignment in the lower extremities was observed in all patients from the both of groups. Group I had an overall 36.4 % complication rate: nail migration—1 case; nail elongation failure—1 case; deformity recurrence—1 case; oblique femoral shaft fracture– 1 case. Group II—16.7 % complication rate: soft tissue infection—1 case; ankle joint infusion due to soft tissue irritation by fixation pin—1 case.
*Conclusion*: Patients from Group 2 showed lower rate of complications compared with the control group. Early results of RSTINs implantation for the patients with OI are promising though further observation is required.

**April 17**
Scientific session: Multicentre + ostegenesis imperfecta + innovative
14:25–15:45
AUDITORIUM
**OP72**/15:11–15:17

## Long-term functional outcome of humeral rodding in children with osteogenesis imperfecta*

Elizabeth Ashby[1], Kathleen Montpetit[1], Reggie C. Hamdy[1],
Francois Fassier[1]

[1]Shriners Hospital, Montreal, Canada

**LEVEL-IV**

*Purpose*: To assess the long-term impact of humeral rodding on functional ability, grip strength, joint range of motion and cosmetic appearance in children with Osteogenesis Imperfecta.
*Materials and methodology*: A retrospective chart review was conducted on 35 children with osteogenesis imperfecta who underwent humeral rodding at our institution between 1995 and 2013. Fassier-Duval rods were inserted in 19 cases, K-wires in 13 cases and rush rods in 3 cases. Functional ability was assessed using the self-care and mobility domains of the Pediatric Evaluation and Disability Inventory (PEDI). Grip strength was measured using a dynamometer and joint range of motion was measured with a goniometer. Deformity was measured on antero-posterior (AP) and lateral radiographs. Outcomes were assessed pre-operatively and every year post-operatively. Differences between pre-operative and 1-year post-operative outcomes were compared using paired T-tests. In 26 patients with a minimum 2-year follow-up, outcome measures at 1-year post-surgery were compared to those at the latest clinic visit (mean follow-up time = 7.0 years, range 2.0–18.5 years).
*Results:* Humeral rodding resulted in a significant improvement in PEDI self-care score (mean change = +5.75, p = 0.028) and PEDI mobility score (mean change =+3.59, p = 0.008) at 1 year post-surgery. The PEDI self-care score was maintained or improved further in 92 % of children at the latest clinic visit. The PEDI mobility score was maintained or improved further in 73 % of children at the latest clinic visit. Shoulder forward flexion and elbow flexion significantly improved following surgery (p = 0.039 and p = 0.024 respectively). This movement was maintained or improved further in 85 % of children at the latest clinic visit. There was no significant change in shoulder abduction or elbow extension following surgery. Surgery resulted in a significant improvement in radiographic angular deformity of the humerus (mean change on AP view = 43.7° p = <0.0001, mean change on lateral view = 45.0° p = <0.0001). There was no significant improvement in grip strength at 1-year after surgery. There were post-operative complications: 7 cases with prominent metalwork, 3 cases of non-union, 1 case of recurrent deformity and 1 case of superficial infection. There was no deep infection, compartment syndrome or physeal growth arrest.
*Conclusions:* Humeral rodding in children with osteogenesis imperfecta leads to long-term improvement in functional ability, range of joint motion and cosmetic appearance. Grip strength does not change.
*Significance:* This is the first case series reporting long-term improvement in function following humeral rodding in children with osteogenesis imperfecta.

**April 17**
Scientific session: Multicentre + ostegenesis imperfecta + innovative
14:25–15:45
AUDITORIUM
**OP73**/15:23–15:29

### New EOS imaging protocol allows a 50-fold reduction in radiation exposure for scoliosis patients

Peter Newton[1], Fredrick Reighard[1], Carrie Bartley[1], Yash Khandwala[2], Tracey Bastrom[1], Burt Yaszay[1]

[1]Rady Children's Hospital, San Diego, United States
[2]University of California, San Diego, United States

**LEVEL-II**

*Aim:* To evaluate the reliability of 3D spinal reconstructions from biplanar x-rays utilizing 5 to 8-fold reduction in radiation dosage compared to the current EOS low dose images (8-fold less than traditional XR) utilized for evaluating patients with adolescent idiopathic scoliosis (AIS).
*Methods:* After IRB approval, 60 AIS patients (30 non-op, 30 post-op) who received "standard", biplanar, antero-posterior and lateral low dose spine x-rays in our EOS imaging unit ($\sim$0.31 mGy) as part of routine care, also underwent an additional set of "microdose" EOS x-rays ($\sim$0.06 mGy) using a new protocol. Two reviewers created 3D reconstructions, twice, of each set of images using sterEOS software (Standard low dose × 2, Microdose × 2). Typical measurements of scoliosis were made: coronal Cobb angles, sagittal (T1–T12, T4–T12, L1–L5, L1–S1), and apical axial rotation. Interrater reliability was also assessed on standard 2D measurements done on the low dose and microdose images. Intraclass correlations (ICC) and standard error of measurement (upper bound of 95 % confidence interval) for the differences between low dose and microdose image measurements were compared.
*Results:* The correlations (ICC's) were rated as 'substantial' to 'almost perfect' for low dose 3D, microdose 3D, and 2D measures (range 0.78–0.99). The calculated error in measurement was not significantly greater in the microdose images for all variables except for intrarater 3D error on 3D L1–L5 lordosis (2.9° microdose vs. 2.2°, p = 0.04), interrater 3D rotation of the apex of the lumbar curve (2.6° microdose vs. 1.7°, p = 0.03), and 2D T12-sacrum lordosis (4.6° microdose vs. 3.4°, p = 0.04). Although the images have slightly less clarity and detail, the critical measures of the curvature were reliably measured.
*Conclusion:* Good reliability was found between 3D and 2D measurements of the low dose EOS images and the microdose images in patients with idiopathic scoliosis. A statistically significant difference in mean measurement error was observed in both 3D and 2D measures of lordosis and 3D lumbar apex rotation. However, the magnitude of difference in error is not clinically important ($\sim$ 1° avg difference).
*Significance:* There is a strong suggestion that radiation exposure can be further reduced with EOS imaging in scoliosis patients, achieving an overall reduction in radiation of approximately 50 times less than standard xrays. This represents a major reduction in this group of patients who often require serial exams during their growing years.

**April 17**
Scientific session: Multicentre + ostegenesis imperfecta + innovative
14:25–15:45
AUDITORIUM
**OP74**/15:29–15:37

### Ultra low dose imaging for the follow-up of idiopathic scoliosis

Emmanuelle Ferrero[1], Marianne Alison[1], Guy Sebag[1], Keyvan Mazda[1], Brice Ilharreborde[1]

[1]Robert Debre Hospital, Paris, France

**LEVEL-III**

*Introduction:* In adolescent idiopathic scoliosis (AIS), repeated radiological monitoring is required to determine curve prognosis and the best therapeutic strategy. Radiosensitivity of the organs in paediatric patients is higher, and a greater risk of breast cancer has been demonstrated in this population. Therefore, minimizing radiation exposure according to the ALARA concept (as low as reasonably achievable) is essential. A biplanar slot scanning system, previously validated in the literature, allows significant dose reduction with three dimensional (3D) reconstructions. With recent technical advances, further dose reduction can be obtained with this system, at a cost of a slight reduction in image quality. The aim of our study was to analyze the reproducibility of this new protocol and evaluate its use in routine clinical practice.

*Methods:* In this single center prospective study, paediatric patients with idiopathic scoliosis were included. All the patients underwent full spine EOS imaging (EOS imaging, Paris, France) using a micro dose protocol. Three observers performed each on two occasions 3D reconstructions of the spine with "fast spine" SterEOS software (EOS imaging, Paris, France). Usual coronal and sagittal spino-pelvic parameters were measured. Reproducibility and accuracy of the measurement was assessed with the ISO norm 5725–2.
*Results:* Thirty six patients were included, with a mean age of 12.8 years, and a mean Cobb angle of 25° (10°–68°). Radiation dose with EOS microdose was 6 times less than with standard EOS (63 µGy). The mean time needed for 3D "fast spine" reconstruction was 5 min. Interobserver reproducibility was 2°–5° for spinal parameters, and 1°–5° for pelvic parameters. Intraclass coefficients of correlation were between 0.86 and 0.98.
*Conclusions:* Measurement reproducibility of EOS "fast spine" microdose is similar to standard EOS reproducibility reported in the literature. 3D reconstructions of the main spinal parameters are faster. While the quality of images is slightly inferior, the radiation dose is very low: 40 times less than a standard x-ray. Therefore, EOS "fast spine" microdose is as reliable as standard EOS and can be considered for routine radiographic follow-up of AIS patients.

**April 17**
Paediatric Elbow Trauma (supracondylar fractures)
16:15 - 17:30
AUDITORIUM
**OP99**/16:15 - 16:21

## Fracture Classification and Nerve Injury Predict Functional Outcomes in Supracondylar Humerus Fractures: A Prospective Study*

Justin Ernat[1], Anthony Riccio[2], Robert Wimberly[2], David Podeszwa[2], William Gheen[3], Christine Ho[2]

[1]Tripler Army Medical Center, Honolulu, United States
[2]Texas Scottish Rite Hospital, Dallas, United States
[3]Childrens Medical Cener, Dallas, United States

**LEVEL-II**

*Purpose:* To evaluate prospectively the relationship between fracture classification, neurological deficit at presentation and functional outcome in children with supracondylar humerus fractures (SCHFX) using validated outcome measures.
*Methods:* An IRB approved prospective enrollment of consecutive patients with operative SCHFX was performed over a 3-year period. Fracture pattern and Gartland classification were recorded. The presence and type of any neurological deficit was documented by the treating surgeon at presentation and throughout the follow-up period. Functional outcome was assessed at final follow-up using the Pediatric Outcomes Data Collection Instruments (PODCI) and the quick Disabilities of the Arm, Shoulder, and Hand (QuickDASH) Outcome Measure. Multiple regression analysis was used to determine the relationship between fracture classification/pattern, presence/type of nerve injury and functional outcome while controlling for other injury parameters including patient age, vascular abnormality and the presence of an open fracture.
*Results:* 752 patients were enrolled during the study period of which 199 (average age 6.7 years) completed functional outcome measures at final follow-up. The average PODCI global functioning scale score and QuickDASH scores for the entire cohort were 93.5 and 10.5 respectively indicating excellent function. 10 patients (5%) sustained flexion injures and 189 (95%) sustained extension injuries of which 62 (33%) were Type II fractures and 127 (67%) were Type III fractures. 12 patients (6%) had multidirectional and instablity. No differences in outcome scores were noted between patients with Type II and Type III fractures, or those with multidirectional instability. For extension injuries, no difference in outcome was identified based upon fracture pattern. Flexion injuries demonstrated significantly lower PODCI transfer and basic mobility (93.9 vs. 98.7) ($p<0.02$) than flexion injuries.

**April 17**
Paediatric Elbow Trauma (supracondylar fractures)
16:15 - 17:30
AUDITORIUM
**OP76**/16:21 - 16:27

## The necessity of fixation in supracondylar fractures of the distal humerus Gartland type 2 (Modified Gartland type 2A and 2B)

Perajit Eamsobhana[1]

[1]Siriraj Hospital, Bangkok, Thailand

**LEVEL-II**

*Purpose:* In Gartland type 2 supracondylar fracture, closed pinning has been recommended in most patients. However a stable, mildly displaced fracture can be treated by nonsurgical means. This study evaluates retrospectively the results of closed pinning versus conservative treatment in this group of patients.
*Method:* Gartland type 2 supracondylar fracture patients were categorized into two groups. First group (type 2A) was defined as a Baumann angle (BA) which differed from the uninjured side by less than 5°. The patient with BA difference > 5 ° was determined as type 2B. Statistical analysis was performed between the two groups. In type 2A, we also analyzed loss of alignment in patients who underwent closed reduction without fixation to clarify the necessity of fixation.
*Results:* Type 2A had a median 3° BA difference from uninjured side, a median 19.5° lateral capitellohumeral angle (LCHA) difference from uninjured side and shaft condylar angle (SCA) difference of 18° from uninjured side. Type 2B had median 8° BA difference, 27° LCHA difference and 28° SCA difference. BA, LCHA and SCA difference was statistically significant (p value = < 0.001, 0.019 and 0.002 between two types, respectively). In type 2A, patients who were treated without fixation, had a 6° improvement in SCA and 11° improvement in LHCA from post-reduction radiographs, with statistical significance (p value = 0.018 and < 0.001 respectively). ROC curve in Type 2A, show that if LCHA difference or SCA difference from uninjured side < 18 °, it is stable enough to maintain reduction without fixation.
*Conclusion:* A modification of the Gartland Type 2 fractures into Type 2A and 2B allows orthopedic surgeons to select the appropriate treatment for the patient. In Type 2A, if patients have LCHA difference or SCA difference from uninjured side < 18 °, K wire fixation seems unnecessary. In type 2B, fixation was recommended in all patients due to the displacement with loss of Baumann angle, LCHA and SCA.

**April 17**
Paediatric Elbow Trauma (supracondylar fractures)
16:15 - 17:30
AUDITORIUM
**OP77** / 16:27 - 16:33

## Is Timing Of Surgery Of Paediatric Supracondylar Fractures Related To Pinning Errors Or Complications?*

Luis Moraleda[1], Maria Valencia[2]

[1]Hospital Universitario La Paz, Madrid, Spain
[2]Hospital Ramon y Cajal, Madrid, Spain

**LEVEL-III**

*Purpose:* The aim of this study was to determine if there is a relation between timing of surgery for paediatric supracondylar fractures and the incidence of pinning errors or complications.
*Materials and Methods:* 454 children with a supracondylar humeral fracture were reviewed. Those that were treated surgically with closed reduction and percutaneous pinning were included, leaving a group of 320 fractures (70%). Data regarding age at the time of fracture, sex, side involved, time of admission, time of surgery, duration of surgery and complications were recorded. Radiographs were reviewed to assess type of fracture, direction of displacement, number of pins, pinning site and pinning errors. Pinning errors were classified according to Sankar et al.
*Results:* Mean age at the time of fracture was 6.5 years (SD 4.7). There were 78% Gartland III, 19% Gartland II and 3% flexion fractures. Patients presented at the ER department mainly during the afternoon and evening. Surgery was performed mainly during evening and and at night. Among patients who were admitted during the morning (8.5 hours), the mean duration of surgery was 35 minutes (median 30). A medial pin was used in 14% of the patients. Pinning configuration was as follows: divergent in 25%, parallel in 25% convergent in 36% and crossed 14% of the cases. Pinning errors occurred in 45% of the patients being type A 3%, type B 14% and type C 28%. Complications occurred in 33% of the patients: neurological injury (6%), vascular injury (2%), loss of reduction (3%), infection (1.5%), and cubitus varus (5%). Pinning errors were detected more frequently following surgeries performed at night ($p = 0.069$). Time delay to surgery was not related to pinning errors ($p = 0.933$) or complications ($p = 0.257$).
*Conclusion:* Time delay to surgery was not related to pinning error or complications. However, pinning errors were more frequent in surgeries performed during the night

**April 17**
Paediatric Elbow Trauma (supracondylar fractures)
16:15–17:30
AUDITORIUM
**OP78**/16:41–16:47

## Delay reduction in supracondylar fractures*

Ignacio Sanpera[1], Enrique Rodriguez de la Rubia[2]

[1]Hospital Son Espases, Palma de Mallorca, Spain
[2]Hospital son Espases, Palma de Mallorca, Spain

**LEVEL-III**

*Purpose*: The safety of delayed reduction of uncomplicated supracondylar fractures has been widely accepted. The objective of this study was to evaluate if the time from fracture to reduction had some relationship to the ease and quality of reduction.
*Methods:* A retrospective review of 169 Type 2 and 3 supracondylar fractures was undertaken (42 Type2 + 127 Type3). The elapsed time from the fracture until surgery was recorded and the duration of the surgery, and quality of reduction assessed. Patients were divided in 2 groups those operated: <6 h since the fracture (early group), and those >6 h (late group). The surgical time was recorded, and the quality of reduction. Type 3 supracondylar fractures were studied to see if an anatomical reduction was achieved.
*Results:* The average duration of surgery in Type 2 supracondylar was 30 min for both groups (early and late); the average duration for Type 3 supracondylar fractures was of 40 min for the early group, and 50 min for the late group. However, this difference was not statistically significant ($p = 0.08$). In Type 3 supracondylar no differences were found in the quality of reduction between the early and late groups, an anatomical reduction was achieved in 53.7 % of the early group compared with 56 % in the late group. Even when the cases with associated injuries were removed, no differences in the surgical time or quality of the reduction was found.
*Conclusion:* This study shows that delayed reduction of supracondylar fractures is not only safe but also that is not associated with an increase in the number of surgical complications or the difficulty with the procedure
*Significance:* This study confirms that surgery for supracondylar fractures Type 2 and 3 can safely be delayed with no risk for the patients.

**April 17**
Paediatric Elbow Trauma (supracondylar fractures)
16:15–17:30
AUDITORIUM
**OP79**/16:47–16:53

## Acute vascular injuries in 404 gartland Type III supracondylar fractures of the humerus in children: urgency management and therapeutic consensus*

Louahem Msabah Djamel[1], Mazeau Philippe[2], Cottalorda Jerome[2]

[1]CHU LAPEYRONIE, MONTPELLIER cedex 5, France
[2]CHU LAPEYRONIE, MONTPELLIER, France

**LEVEL-III**

*Purpose of the study*: The treatment of the acute vascular injuries in Gartland Type III supracondylar humeral fractures in children is still debated. Our experience of 20 years based on the urgent management of 404 cases and their vascular complications allowed us to determine a therapeutic consensus in France.
*Materials and methods:* 404 children (average age 7.2 years) were treated for GartlandType III acute supracondylar fracture of the humerus. Extension-fractures occurred in 383 (95 %) and acute vascular complications in 68 cases (17 %). 33 (8 %) had associated nerve injuries. Median nerve impairment was the most common, and occurred in 87 % of cases. The radial pulse was absent in 68 patients with two clinical situations: a well perfused hand (WPH) in 63 (16 %) cases and an ischaemic Hand (IH) in 5 (1 %). Emergency management included repeated assessment of the vascular and neuroloical status using a departmental protocol involving Doppler control together with oxygen saturation. Emergency anatomical reduction and stable percutaneous fixation, with lateral and medial wires via a minimal incision to control the ulnar nerve, was performed in all cases. Postoperatively the patientwas closely observed. Angiography and MRI post-operatively were required to identify late-developing vascular compromise when the distal perfusion of the limb deteriorates.
*ResultsL:* 63 (93 %) of the cases with vascular compromise had postero-lateral displacement. After closed reduction and percutaneous pinning performed in 63 cases of WPH, the radial pulse was restored immediately in 42 (67 %) cases and secondarily in 18 (28 %). The 3 (5 %) remaining cases with an absent radial pulse with WPH developed ischemia necessitating surgical exploration revealing incarceration of the brachial artery and medial nerve within the fracture site. Release of the brachial artery restored a well perfused hand. The pulse radial return was postoperatively observed between few hours to 11 days. The 5 cases of primary ischemia underwent open exploration and vascular repair which restored blood flow.
*Conclusion*: This study identified the following points and determine our defensive therapeutic strategy: priority is required for closed reduction of these fractures and emergency percutaneous stabilization. Postero-lateral displacement is associated with a higher risk of vascular complications. The absence of a radial pulse with a pink hand warrants repeated observation during the postoperative period; it is not an absolute indication for immediate invasive investigation and surgical exploration. The absence of a pulse with a white hand or secondary changes of the distal perfusion requires surgical vascular exploration.

**April 17**
Paediatric Elbow Trauma (supracondylar fractures)
16:15–17:30
AUDITORIUM
**OP80**/16:53–16:59

## Long-term outcome of vascular and nerve complications in supracondylar fractures of the humerus during childhood

Dirk Sommerfeldt[1]

[1]Altonaer Kinderkrankenhaus, Frankfurt, Germany

**LEVEL-I**

*Introduction/background:* Supracondylar fractures of the humerus are common injuries during childhood. Vascular or nerve injuries occur in approximately 10 % of these fractures. A case series of 345 patients of which 50 patientes had a concomitant nerve or vascular injury were analyzied to review our treatment regimen for this trauma entity.
*Materials and methods:* 345 patients over 6 years were treated. 21 of these patients (6 %) had a concomitant vascular compromise and 29 patients (8.4 %) were diagnosed with a nerve damage. These subgroups were stratified for age, sex, type of trauma, localization of nerve or vascular damage, and short and longterm outcome. 45 patients were seen for long-term follow-up in a time frame between 10 months and 6 years 7 months (0.9–6.6 years) post injury.
*Results:* Time to surgery post trauma was 4.25 h on average for this selected group of patients. All patients were treated with crossed K-wire osteosynthesis. In 10 patients open reduction was necessary. Average hospitalization time was 5.7 days on average (range 2–17 days). In six of 29 patients (20 %) with nerve complications symptoms occurred postoperatively, and were most likely iatrogenic. In 11of 21 patients (52 %) with vascular compromise pulse returned after reduction of the fracture, but in 10 patients (47 %) the vessel had to be visualized, and in 3 cases (14 %) venous grafting was necessary. 44 of 45 patients (98 %) had good to excellent results at long-term follow-up. There were no cases of compartment syndrome or Volkmann's contractures. All three reconstructed brachial arteries remained open without any steal phenomenon during exercise.
*Conclusion:* The prognosis for concomitant nerve injuries during supracondylar fractures is good. Surgical intervention is rarely necessary. Vascular injuries should be attended to immediately. Wait-and-see strategies in cases with absent radial or brachial pulses during intraoperative Doppler sonography post fracture reduction cannot be recommended.
*Significance:* These results can be interpreted as further evidence for a straightforward approach when treating supracondylar fractures in children with nerve and/or vascular problems: Nerve injuries are almost always class I (neurapraxia) or class II (axonotmesis) and generally heal without surgical intervention. Vascular injuries however should not be left unattended and the vessel should be visualized and decompressed, liberated, or reconstructed, if pulse does not return after fracture reduction, even when collateral cubital vessels may be able to sustain blood flow to the "pink and pulseless" hand. Patency rates following vascular reconstruction are high.

**April 17**
Paediatric Elbow Trauma (supracondylar fractures)
16:15–17:30
AUDITORIUM
**OP81**/16:59–17:05

## The ao classification of supracondylar fracture and its influence on decision making

Nicolas Lutz[1]

[1]University of Lausanne Hospital, Lausanne, Switzerland

**LEVEL-I**

*Introduction:* Several classifications have been used to describe and categorize supracondylar fractures. Most treatment modalities are based on classification and influenced by other factors such as the expertise of the surgeon, the quality of the radiographs, the mechanism of injury, the age of the child and the associated neuro-vascular injuries. The AO classification was developed to be user friendly in assessing the degree of displacement and prospectively validated as being reliable. Its influence on decision making was never evaluated.
*Method:* This is a Critical review of the literature assessing the various classifications and treatment modalities. It is a local retrospective review of supracondylar fractures classification and treatment modalities.
*Results:* The Gartland, Lagrange-Rigault and AO classifications were subject to reliability studies with contrasting results. They were widely used in recent descriptive and comparative studies with multiple treatment modalities. They were never compared. A trend for conservative treatment restricted to non-displaced fractures (Grade I) was noted. 414 supracondylar fractures managed in our institution since 2009 were classified using the AO algorithm, including 192 Grade I, 71 Grade II, 62 Grade III and 84 Grade IV. Treatment was conservative in Grade I and surgical in Grade IV. More detailed evaluation of the treatment modalities in Grade II and III will be presented.

*Conclusion:* The influence of a validated classification on treatment modalities varied and was influenced by non-classification related factors such as the mechanism of injury (flexion v extension). The AO classification was reliable in identifying stable fractures.
*Significance:* Incorporation of factors such as mechanism of injury in the algorithm of the AO classification may improve decision making and outcome.

**April 18**
Scientific session: upper extremity + musculoskeletal infections
08:00–09:20
AUDITORIUM
**OP82**/08:00–08:06

## Prophylactic simple resection of ulnar tumor for deformities of the forearm in multiple osteochondromas

Mitsuaki Morita[1], Makoto Kamegaya[1], Takashi Saisu[1], Yuko Segawa[1], Jun Kakizaki[1], Reiko Tanaka[1]

[1]Chiba Pediatric Orthop. Group, Chiba, Japan

**LEVEL-IV**

*Background:* Osteochondromas often results in deformities of the forearm. The characteristic deformities are ulnar shortening, bowing of the forearms, and dislocation of the radial head. The treatment for dislocation of the radial head is often difficult and may need several operations to correct it. These include lengthening of the ulnar and open reduction. We investigate the forearms in the patient of multiple osteochondroma with or without involvement of the forearms. If we find osteochondroma in the ulnar, we try to resect them early regardless of tumor size and deformity of the bone, and we had good outcomes in some cases. The purpose of this study was to investigate the effects prophylactic simple resection of the ulnar osteochondromas.
*Methods:* We reviewed five forearms in five patients who underwent simple excision of osteochondroma in the ulnar. All tumors were located in the distal part of the ulnar. The average age at the time of surgery was 5.4 years (range 3.2–7.8), and the average follow-up period was 38 months (range 10–117). The percentage of ulnar shortening compared to the radial length, the percentage of radial bowing (the ratio of radial bowing to radial length) and radial articular angle were measured as index of the forearm deformity.
*Results:* The average percentage of ulnar shortening improved from 10.9 % before surgery to 3.2 % at final follow-up and ulnar shortening were resolved in two cases. The percentage of radial bowing was 9.0 % before surgery and 8.7 % at final follow-up, radial articular angle was 36.0 before surgery and 34.8 at final follow-up.
*Conclusions:* Prophylactic simple resection of osteochondroma in the distal ulnar can stop progression, resolve ulnar shortening and prevent severe forearm deformity.

**April 18**
Scientific session: Upper extremity + musculoskeletal infections
08:00–09:20
AUDITORIUM
**OP83**/08:06–08:12

## Evaluation of the results ulnar lengthening for correction of forearm deformity in multiple hereditary exostosis, preliminary results

Amin Abdelrazek Ahmed[1]

[1]Alexandria University, Alexandria, Egypt

**LEVEL-IV**

*Introduction:* Bony exostoses are benign osteocartilaginous growths that start close to growth plates. Thirty to 60 % of patients have forearm deformities. Commonly seen deformities in this condition include bowing of the radius, shortening of the ulna, ulna drift of the carpus, and occasionally dislocation of the radial head.The current study reported on the results of management for type IIb Masada and Ono classification of forearm deformities by Ilizarov ulnar lengthening
*Patients and methods:* A series eight patients with type IIb Masada deformity were treated by Ilizarov lengthening at AlHadra University Hospital, Alexandria, Egypt during the period of January 2008–June 2011. There were 6 males and 2 females; right forearm was affected in 5 patients. The mean age is 8.6 years. All cases showed ulnar shortening with dital ulnar exostosis and radial head dislocation type IIb. A detailed functional assessment was conducted before and after correction.All patients were operated under general anaesthesia, with application of Ilizarov frame to the forearm. The frame used was assembled of 2 complete rings, the proximal one was fixed to the proximal ulna using a wire and two 4 mm Shanz screws. The distal ring was fixed to both radius and ulna using a transverse wire, and the stability was reinforced by two 4 mm Shanz screws fixed to the ulna. Ulnar osteotomy was done between the rings. Lengthening was started after 10 days to lengthen the ulna and pull the radius down. All cases showed spontaneous reduction of the head radius and correction of the forearm deformity.
*Results:* At a period of follow up of 21 months (12–36 months) all patients showed spontaneous reduction of the radial head and correction of the forearm deformity. The range of motion improved: flexion increased from 105° to 130°, extension reached to 10° while it was 25° preoperatively and the supination increased from 30° preoperatively to 55° postoperative. All these were statistically significant. All patients and their parents were fully satisfied at the end of follow up. No recurrence of the deformity was noted during the latest follow-up. All the gains in the motion of the forearm were maintained at the last follow-up. The mean duration of the period of external fixation in this group of patient was 8.8 weeks.

*Conclusions:* Ilizarov lengthening of the ulna is an excellent method for correction of forearm deformity and to improve elbow range of motion in patients with hereditary multiple exostosis

**April 18**
Scientific session: Upper extremity + musculoskeletal infections
08:00–09:20
AUDITORIUM
**OP84**/08:12–08:18

## Radiographic criteria for undergoing an ulnar shortening osteotomy in madelung deformity: a long-term experience from a single institution

Sebastian Farr[1], Leslie A. Kalish[2], Donald S. Bae[2], Peter M. Waters[2]

[1]Orthopedic Hospital Speising, Vienna, Austria
[2]Childreńs Hospital Boston, Boston, United States

**LEVEL-IV**

*Background:* There are no established guidelines regarding the age or severity of deformity for which an ulna shortening osteotomy or ulna epiphysiodesis should be performed in children and adolescents with Madelung deformity. The purpose of this study was to identify radiographic criteria associated with the eventual need for an ulna shortening procedure in this patient population.
*Methods:* We retrospectively identified 41 wrists in 31 Madelung patients (mean ± SD age 13.8 ± 3.2 years), which underwent surgical correction of their deformity between 1999 and 2013. We assessed established radiographic criteria (ulnar tilt, lunate subsidence, palmar carpal displacement, ulnar variance) at pre- and postoperative visits. Univariate and multivariate analyses were utilized to determine which radiographic criteria were associated with the need for "ulna shortening" at the first (index) surgical procedure.
*Results:* 11 wrists underwent an ulna shortening osteotomy at the primary, and 5 at subsequent procedures; 10 cases received an ulnar epiphysiodesis (mean age 13.4 ± 1.5 years). Ulna shortening at the primary procedure was associated with significantly higher lunate subsidence, ulnar variance and palmar carpal displacement. Ulnar variance of greater than 5 mm and lunate subsidence greater than 4 mm resulted in a respective 67 %, and a 53 % likelihood of undergoing ulnar shortening osteotomy. While palmar carpal displacement over 22 mm resulted in a 50 % likelihood for ulnar shortening. Patients who required a subsequent procedure (n = 8) showed a significant increase in palmar displacement between both surgeries. None of the ten cases with a primary ulna epiphysiodesis needed a subsequent ulna shortening; none of those undergoing late ulna shortening had an ulna epiphysiodesis as their index procedure (at 10.3 ± 4.3 years).
*Conclusions:* Lunate subsidence, ulnar variance and palmar carpal displacement were significant radiographic criteria for undergoing an ulnar shortening osteotomy at our institution. A shortening osteotomy may be prevented by early ulna epiphysiodesis in skeletally immature children over 10 years of age.

**April 18**
Scientific session: Upper extremity + musculoskeletal infections
08:00–09:20
AUDITORIUM
**OP85**/08:28–08:34

## Vertical scapular osteotomy in congenital high scapula

Tarek Abdelaziz[1]

[1]Ain Shams University, Cairo, Egypt

**LEVEL-IV**

*Background:* Children with congenital high scapula (CHS) have a cosmetic and functional problem due to limited shoulder abduction. Treatment options include excision of prominent superior angle, scapular relocation procedures and subtotal scapulectomy. Excision of superomedial angle results only in cosmetic improvement. Subtotal scapulectomy and relocation procedures are associated with ugly scars, extensive bleeding and high incidence of brachial plexus injuries. Vertical scapular osteotomy (VSO) is another surgical option that provides cosmetic and functional improvement. The aim of this study is to assess medium to long term results of VSO in treatment of CHS.
*Methods:* This is a prospective case series study. Seven children with CHS were treated at our unit. Age ranged from 5 to 13 years, average 8.4 years. All children were females with unilateral affection. All children underwent a VSO as described by Campbell. We used the Cavendish grading system together with combined shoulder abduction for assessment. Follow up averaged 4.6 years.
*Results:* All children and parents were extremely satisfied with the result of surgery. All patients experienced an improvement in global shoulder abduction with an average gain in abduction of 52.9°. All patients experienced an improvement in cosmetic appearance with near levelling of the shoulders, The Cavendish grade improved in all patients.
*Conclusion:* This study emphasizes the results of previous authors that CHS can be treated successfully with a VSO. The procedure is simple and its results are reproducible.

**April 18**
Scientific session: Upper extremity + musculoskeletal infections
08:00–09:20
AUDITORIUM
**OP86**/08:34–08:40

## The choice of donor site for reconstruction of elbow flexion in patients with amyoplasia

Olga Agranovich[1], Alexey Baindurashvili[2]

[1]The Turner Institute, St.-Petersburg, Pushkin, Russian Federation

[2]The Turner Scientific and Research Institute for Children's, St. -Petersburg, Russian Federation

**LEVEL-II**

*Introduction:* The choice of donor site for children with arthrogryposis who require reconstruction of elbow active flexion is extremely limited. Latissimus dorsi, pectoralis major and triceps brachii are the most frequently used muscles for this purpose. The goal of this report is to determine the optimal donor site for reconstruction of elbow flexion in patients with amyoplasia.
*Materials:* From 2008 to 2014 we performed reconstruction of elbow flexion in 103 children with amyoplasia by transposition of 142 muscles from different sites (53—pectoralis major (PM) 33- latissimus dorsii (LD) 35—long head of triceps brachii (LHTB) 12—triceps brachii (TB) 9—pectoralis major et minor). The age of patients was from 10 months to 14 years.
*Results:* The outcome results were assessed in 115 cases from 6 months to 5 years after operation. We compared the results of transposition of different muscles to biceps brachii and found out that the best results were in patients with latissimus dorsii muscle transposition, the good results with pectoralis major muscle transfer, good and satisfactory results after long head of triceps brachii, poor results after triceps to biceps brachii transfer. According our classification (Agranovich O., Lahina O., 2013) all patients with amyoplasia were divided into 3 groups which differ from each other by the level of damage of the spinal cord (C6–C7, C5–C7, C5–Th1). We performed correlation between level of damage of the spinal cord and structure of donor muscles. The patients with C6–C7 spinal cord disorders had the best structure of muscles (latissimuss dorsii, pectoralis major, long head of triceps brachii, triceps brachii). The worst structure of muscles was in children with the level of damage of the spinal cord C5–T1.
*Conclusion:* Preoperative examination of patients with amyoplasia with identification of the level of the spinal cord disorders helps to choose the optimal form of treatment and restore daily activity of children with this pathology. In cases of level C6–C7 for reconstruction of elbow flexion we recommended latissimuss dorsii muscle transfer. In patients with level C5–C7 we prefer latissimuss dorsii muscle or pectoralis major transfer. In cases with total disruption (level C5–Th1) we recommended to use pectoralis major muscle or the long head of triceps brachii.

**April 18**
Scientific session: Upper extremity + musculoskeletal infections
08:00–09:20
AUDITORIUM
**OP87**/08:40–08:46

## Is there a role for conservative treatment in pediatric trigger thumb? A systematic review

Sebastian Farr[1], Rudolf Ganger[1], Werner Girsch[1]

[1]Orthopedic Hospital Speising, Vienna, Austria

**LEVEL-IV**

*Background:* Despite its frequent occurrence, there still remains controversy over the optimal method of treatment for paediatric trigger thumb. Most authors recommend open or percutaneous surgery to release the A1 pulley. However, recent reports have suggested that nonoperative care (e.g. splinting, passive exercising) may be a reasonable treatment option. The purpose of this systematic review was to determine the outcome of interphalangeal (IP) joint motion in children undergoing open surgical release, splinting, and passive exercising therapy for the treatment of paediatric trigger thumb.
*Methods:* We conducted an online literature search of seven major databases. Only studies with a mean follow-up of at least 12 months were considered for inclusion. Studies of percutaneous release techniques were excluded because technique differs widely between available reports. Methodology studies was assessed according to Zaza et al. (2000). Seventeen retrospective studies and one prospective study met all the inclusion criteria. They reported on the results of surgery (634 children, 759 thumbs), splinting (115 children, 138 thumbs), and passive exercising (89 children, 108 thumbs). The mean follow-up periods were 59 months (surgery), 23 months (splinting), and 76 months (exercising), respectively. The mean age of all patients included in this review was 32 months (range 15–90).
*Results:* Children treated with surgery had a higher mean age (35 months; range 15–90) than those at the time of first splint application (26 months; range 19–36) or at the start of exercising (27 months; range 26–28; $p = 0.549$). Overall, full active and passive IP joint motion without residual triggering was achieved in 95 % (range 82–100) of all children undergoing surgery. Compared with surgery, children treated by consecutive splinting therapy showed a lower rate of full IP motion (67 %; range 39–92; $p = 0.007$). Passive exercising, however, showed the worst outcome rates compared with surgery (55 %; range 40–80; $p = 0.002$). When comparing splinting to passive exercising, no significant difference was observed ($p = 0.660$).
*Conclusion:* Based on the low level of evidence available, it seems that open surgery resulted in more reliable and rapid outcomes compared with nonoperative treatment. In further studies, however, the success rate of splinting should be assessed for both flexible and fixed thumb contractures independently, as well as for younger children (e.g., <1 year) to determine if it might be an option at least for these cases.

**April 18**
Scientific session: Upper extremity + musculoskeletal infections
08:00–09:20
AUDITORIUM
**OP88**/08:54–09:00

## Osteomyelitis is commonly associated with septic arthritis of the shoulder in children

Robert Wimberly[1], Justin Ernat[2], Anthony Riccio[1], Kelly Fitzpatrick[2]

[1]Texas Scottish Rite Hospital, Dallas, United States
[2]Tripler Army Medical Center, Honolulu, United States

**LEVEL-IV**

*Purpose*: To describe the clinical presentation, management, and outcomes of surgically treated septic arthritis of the shoulder in a paediatric population.
*Methods:* A retrospective chart review over 5 years of children with operatively managed septic arthritis of the shoulder was completed. Demographics, clinical presentation, symptoms duration, antibiotic regimen and duration, number of surgical procedures, and evaluation of laboratory value improvements were collected. Pre-treatment and final radiographs were assessed. Causative organisms were reviewed.

Patients were stratified in age groups to determine clinical variability based upon patient age.
*Results:* 22 children, ages 15 days to 14 years (average 37.3 months), were treated for septic arthritis of the shoulder from 2006 to 2010 at a single paediatric institution. All patients were managed with open anterior arthrotomy at an average of 1.95 days after initial orthopaedic consultation (range 0–15 days). Multiple presenting signs were noted; the most common was decreased use (54 %). Average admission laboratory values include CRP 10.6 (0.3–41.6), ESR 62.8 (11–107), and WBC 14.9 (5.9–31.7). Initial radiographs were read as normal in 12 patients, concern for osteomyelitis in 5, cortical irregularity in 1, fracture in 3, and neoplasm in a single child. 20 patients had a pre-operative MRI and 15 demonstrated an effusion, 15 had evidence of humeral osteomyelitis, 5 had a subperisoteal abscess and 4 had soft tissue abscesses. 8 patients remained culture negative. The most commonly identified organism was methcillin resistant staphylococcus aureus (MRSA) (22.7 %). The patients under 12 months of age revealed more diverse organisms at culture and were less likely to have MRSA. All patients averaged 1.55 (1–5) surgical procedures and had an average hospital stay of 13.5 days. Intravenous (IV) antibiotics averaged 16.3 days followed by an average of 34 days of oral treatment. MRSA patients were significantly more likely to require multiple operations to eradicate the infection ($p < 0.02$) and had a longer duration of IV antibiotic use ($p < 0.003$). MRSA patients were more likely to have abnormal radiographs at final follow-up ($p < 0.03$).
*Conclusion:* Septic arthritis of the shoulder in children is commonly associated with adjacent osteomyelitis. Paediatric septic arthritis of the shoulder due to MRSA bacteria can have a more virulent course than other bacterial causes, but is a less commonly identified organism in the youngest patients.
*Significance:* To our knowledge, this is one of the largest series published concerning the treatment, course, and outcomes of pediatric septic arthritis of the shoulder.

**April 18**
Scientific session: Upper extremity + musculoskeletal infections
08:00–09:20
AUDITORIUM
**OP89**/09:00–09:06

### Septic arthritis of the ankle in children

Marcin Zgoda[1], Mohamed Osman[2], Neil Wilson[3]

[1]University Hospital Crosshouse, Glasgow, United Kingdom
[2]Minia University Hospital, Minia, Egypt
[3]Yorkhill Hospital, Glasgow, United Kingdom

**LEVEL-IV**

*Purpose:* The aim of the study was to evaluate the incidence, aetiology and outcome of septic arthritis of the ankle in children.
*Methods:* We included children treated at our institution between January 1996 and December 2008 for haematogenous septic arthritis of the ankle. Among 47 children with diagnosed acute septic arthritis, 14 had septic arthritis of the ankle. The mean incidence of acute haematogenous ankle septic arthritis in children was 0.9/year/100,000 children below 13 years.
*Results:* The mean age of the patients was 5.7 years (range from 8 months to 12 years). The mean duration of symptoms prior to starting proper treatment was 3 days (range from 2 to 12 days). 12 children were treated by joint aspiration as a single procedure. In one case aspiration was followed by immediate arthrotomy. One patient was treated primarily by arthrotomy. All patients were treated with intravenous antibiotics after specimens for microbiology culture were obtained. The most popular choice of antibiotics was: Benzylpenicillin and Flucloxacillin, usually in combination. The mean duration of intravenous antibiotics treatment was 3.5 days (from 2 to 7 days). Total duration of antibiotic treatment was 23 days (from 10 to 42 days). The mean follow up was 16 months (6 to 36 months). In 13 children we obtained good clinical results defined as a full range of motion of the ankle joint without pain and no radiological changes in surrounding bones. All except one patient returned to the pre-infection mobility. One patient developed secondary osteoarthritis.
*Conclusion:* Early joint aspiration followed by short course of intravenous and oral antibiotics proved to be effective in the management of septic arthritis of the ankle in children.

**April 18**
Scientific session: Upper extremity + Musculoskeletal infections
08:00–09:20
AUDITORIUM
**OP100** / 09:06 - 09:12

### Primary Subacute Hematogenous Osteomyelitis In Children: Towards A New Bacteriological Reality

Dimitri Ceroni[1], Raimonda Valaikaite[1], Amira Dhouib[1], Georgios Kampouroglou[1], Davide Salvo[1]

[1]Hôpital des Enfants, Genève, Genève, Switzerland

**LEVEL-II**

*Background:* This study attempted to describe the spectrum of pediatric primary subacute hematogenous osteomyelitis (PSAHO), to investigate its bacterial aetiology, and to modify the previous classification system based on the radiographic appearance of the lesion.
*Methods:* 55 consecutive cases of PSAHO admitted to our institution over a 14-year period were retrospectively reviewed to assess their clinical, radiographic, and magnetic resonance imaging features, as well as their bacteriological aetiology.
*Results:* White blood cell count was normal in 45 cases (81.2%), C-reactive protein in 30 cases (54.5%), whereas the erythrocyte sedimentation rate was greater than 20 mm/hr in 40 cases (75.5%). Blood cultures failed to identify the pathogen in all patients but one, and classic bone sample cultures only managed to isolate it in 4 cases (11.4%). Use of the polymerase chain reaction (PCR) assays on bone aspirates or blood cultures allowed the causative microorganism to be isolated in a further 18 cases. Using classic cultures and PCR assays together resulted in pathogen detection in 22 cases (61.1% of the children bacteriologically investigated), with *K. kingae* being the most frequently reported microorganism. Intraobserver agreement for PSAHO classification based on plain radiographs or MRI was found to be excellent for both observers (kappa = 1 & 0.952 and 0.973 & 0.889, respectively). Interobserver agreement for PSAHO classification was excellent when plain radiographs were studied (kappa = 0.864) and more accurate still when lesions were investigated using MRI or computed tomography scanning (kappa = 0.920).
*Conclusions:* Two distinct forms of PSAHO should be distinguished on the basis of age of patients and bacteriological aetiology. The infantile form affects children aged between 6 months and 4 years and is predominantly due to *K. kingae*. The juvenile form involves children older than 4 years and *S. aureus* appears to be the main bacteriological etiology. Appropriate nucleic amplification assays drastically improve the detection rate of the microorganisms responsible for PSAHO. Finally, using MRI may significantly improve the recognition of PSAHO's lesions before lytic bone lesions occur.

**April 18**
Scientific session: LCP; tumours + innovative
10:40–12:20
AUDITORIUM
**OP90**/10:40–10:46

### Assessment of femoral head revascularization in legg-calvé-perthes disease (LCPD) using serial perfusion MRI

Harry Kim[1], Jamie Burgess[1], Paul Gudmundsson[1], Alec Thoveson[1], Molly Dempsey[1], ChanHee Jo[1]

[1]Texas Scottish Rite Hospital, Dallas, United States

**LEVEL-II**

*Purpose*: Perfusion MRI (contrast-enhanced MRI) can assess the amount and pattern of femoral head vascularity. To our knowledge, no study has quantitatively investigated the revascularization characteristics of the necrotic femoral head in LCPD. The purpose of this study was to determine the pattern and rate of revascularization of the femoral epiphysis and to evaluate if treatment affected this rate.
*Methods:* This is an IRB approved prospective study of 33 patients (33 hips; age 5–16 with mean 9.2 ± 2.3) diagnosed with LCPD during the active stage of disease (Waldenström Stage I or II). All patients had at least 2 perfusion MRIs (pMRI) and 25 patients had 3 or more pMRIs. The average duration between 1st and 2nd MRI, 1st and 3rd MRI, and 1st and final MRI was 5.7, 10.4, and 15.1 months, respectively. Perfusion percentages of the femoral epiphyses were measured by 2 independent observers using a MR image analysis software to determine the rate of revascularization over time. Statistical analyses included ICC and Mann–Whitney U test.
*Results:* Initial pMRIs showed various levels of % perfusion in the affected femoral heads ranging from 5 to 70 %. The average % perfusion was 35 ± 16 % at the 1st MRI which increased to 78 ± 13 % at the follow-up MRI obtained an average of 10.1 ± 2.4 months later ($p < 0.01$). In most patients, the rate of revascularization was faster in the early stage. Serial assessment showed a general pattern of revascularization starting from the periphery of the posterior, lateral, and medial aspects of the epiphysis and converging towards the antero-central region of the epiphysis. The average rate of revascularization was 4.9 ± 2.4 % per month with a wide range between patients (0.5–10.4 % per month). For the 16 patients treated operatively with either epiphyseal/core drilling or a femoral varus osteotomy, the average rate of revascularization was 5.9 ± 2.4 % per month vs. 4.5 ± 2.5 % per month for the 17 patients treated nonoperatively ($p = 0.07$).
*Conclusion:* Revascularization of the affected femoral head increased over time but the rate of revascularization was highly variable between patients. Operatively treated patients appear to have a higher rate of revascularization with statistical analysis showing a trend.
*Significance:* This study provides for the first time information about the pattern and rate of revascularization of the femoral head in LCPD. Further research would help determine which clinical factors affect the rate of revascularization.

**April 18**
Scientific session: LCP; tumours + Innovative
10:40–12:20
AUDITORIUM
**OP91**/10:46–10:52

### Outcome after combined pelvic and femoral osteotomy in patients with legg-calve-perthes disease

Martin Rupprecht, MD[1], Sandra Breyer, MD[1], Nadine Mosow[1], Karsten Ridderbusch, MD[1], Ralf Stücker, Prof.[1]

[1]Altonaer Children's Hospital, Hamburg, Germany

**LEVEL-IV**

*Background:* The purpose of the study was to evaluate the outcome at skeletal maturity after combined pelvic and femoral varus osteotomy in a large single center cohort of children affected by Legg Calve Perthes disease (LCPD).
*Methods*: From January 1998 to December 2009, 69 patients with LCPD underwent the combined osteotomy at our institution. 55 children (20 girls) fulfilled the inclusion criteria and were enrolled in the study. The LCPD was classified and all patients were clinically and radiologically reviewed at skeletal maturity. The final follow-up radiographs were assessed by the Stulberg classification and the Sphericity Deviation Score (SDS).
*Results:* The mean age at diagnosis was 6.9 ± 2.4 years (range 2–13), and 7.9 ± 2.3 years (range 4–15) at operation. The mean follow up was 10.8 ± 3.5 years (range 4.6–17.6). The mean Harris Hip Score (HHS) at FU was 90 ± 13.2 (range 50–100). According to the HHS grading system, 39 patients (67 %) had an excellent outcome, 9 patients (16 %) had a good result, 4 patients (7 %) a fair and 5 patients (9 %) a poor result. There were 7 Stulberg class 1 (13 %), 18 Stulberg class II (33 %), 16 Stulberg class III (29 %), 9 Stulberg class IV (16 %), and 5 Stulberg class V (9 %). The mean SDS was 24.5 ± 14.4 (range 2.3–68.7) at skeletal maturity. According to the age at diagnosis, children <6 years of age had significant better scores (11.9 ± 7.0) than children between 6 and 8 years of age (23.0 ± 8.0, $p = 0.001$) and >8 years of age (37.2 ± 17.3; $p < 0.001$).
*Conclusions*: The combined pelvic and proximal femoral varus osteotomy did not lead to better clinical and radiological results than when each osteotomy is used alone. Therefore, this treatment should be not routinely used for children with LCPD. It should be reserved for patients with severe disease in whom containment of the femoral head is difficult to obtain by more conventional methods.

**April 18**
Scientific session: LCP; tumours + innovative
10:40–12:20
AUDITORIUM
**OP92**/10:52–10:58

### Is quality of life altered in LCPD-patients after intense surgery?

Bettina Prof. Dr. Westhoff[1], Nina-Kristin Palmen[1], Dietmar Rosenthal[2], Christoph PD Dr. Zilkens[1], Ruediger Prof. Dr. Krauspe[1]

[1]Dept. of Orthopaedics, Duesseldorf, Germany
[2]Dept. of Neurology, Duesseldorf, Germany

**LEVEL-II**

*Aim:* The diagnosis Legg-Calvé-Perthes disease (LCPD) has a considerable influence on the daily life of children. During the active period of the disease restriction in their leisure time activities is common, time has to spent with physiotherapists and some even need surgical treatment. All these factors may influence their mood. Until now this aspect of the disease has been almost completely neglected. Therefore we compared the health related quality of life (HRQoL) of children with severe LCPD who had extensive surgery with pelvic and femoral osteotomy with an age and gender matched normal control group.
*Methods:* 17 LCPD-children (16 boys and 1 girl) aged 5–11 years at the time of surgery could be recruited. As a measurement instrument for the HRQoL the KIDSCREEN-10-questionnaire was administered, for the functional status the modified Harris-hip-score (mHHS). Analyses were made for the preoperative period and at least 2 years postoperatively. The follow-up results were compared to an age and gender matched normal control group. Correlations were computed between KIDSCREEN-10 and mHHS pre- and postoperatively.
*Results:* The KIDSCREEN-10-T-value increased from 45.3 (SD 4.6) preoperatively to 70.2 (SD 12.7) postoperatively and was higher than the mean T-value of the control group (57.2 (SD 11.1)). As well the mHHS improved from 54.4 (SD 19.9) to a score of 99.5 (SD 1.5) postoperatively. A strong correlation was found between the preoperative mHHS and the postoperative KIDSCREEN-10-T-value (Spearman's-rho 0.67, $p = 0.003$).
*Conclusions:* Patients with severe LCPD feel a marked reduction in their quality of life during the active stage of the disease. This is independent of the clinical status of their hip joint. After containment improving surgery and a mean follow-up period of 4.2 years. HRQoL is even better when compared with a healthy age and sex matched control group. As well an excellent clinical function could be achieved.

**April 18**
Scientific session: LCP; tumours + innovative
10:40–12:20
AUDITORIUM
**OP93**/10:58–11:04

## Legg-calvé-perthes disease (LCPD) produces significant elevation of pro-inflammatory cytokine interleukin-6 (IL-6) in the synovial fluid and chronic hip synovitis

Nobuhiro Kamiya[1], Ryosuke Yamaguchi[1], Naga Suresh Adapala[1], Elena Chen[1], David Neal[2], Jack O'Brien[2], Alec Thoveson[1], Paul Gudmundsson[1], Case Brabham[1], Hicham Drissi[3], Harry Kim[1]

[1]Texas Scottish Rite Hospital, Dallas, United States
[2]UT Southwestern Medical Center, Dallas, United States
[3]UCONN health center, Farmington, United States

**LEVEL-I**

*Purpose*: Hip joint synovitis is a common feature of LCPD which produces joint irritability, stiffness, and poor outcome. Current management of hip synovitis in LCPD is non-specific as the pathophysiology and mechanism(s) causing the synovitis are unknown. The purposes of this study were to determine the inflammatory cytokines present in the synovial fluid of patients with LCPD and to assess the hip synovitis using serial MRI.
*Methods:* Synovial fluid was obtained at the time of hip arthrography from 13 patientswithLCPD in Waldenström stage 1 or 2. Synovial fluids obtained from 5 patients with DDH were used as controls. A multi-cytokine assay was used to determine the levels of 27 inflammatory cytokines and other factors in the synovial fluid. Serial MRIs were performed on 28 patients with LCPD to assess the chronicity and nature of hip synovitis. T2-weighted and gadolinium-enhanced MR sequences were used to quantify synovial fluid volume and synovial membrane enhancement, respectively. Mechanisms causing hip synovitis and increased interleukin-6 (IL-6) release following ischemic osteonecrosis were investigated using a piglet model of ischemic osteonecrosis and in vitro cell culture experiments. Statistical analyses included Student's t-test for comparison of 2 groups and ANOVA for $\geq$3 groups.
*Results:* Of the major inflammatory cytokines analyzed, only the inflammatory cytokine IL-6 was significantly elevated in the synovial fluid of patients with LCPD (mean $509 \pm 519$ vs $19 \pm 22$ pg/ml in controls, $p = 0.002$). The other major cytokines, IL-1$\beta$ and TNF-$\alpha$, were not elevated. Serial MRIs showed significantly increased synovial effusion ($p < 0.001$) and synovial membrane enhancement ($p < 0.001$) in the affected hip at the follow-up MRI obtained $17.7 \pm 8.3$ months after the initial MRI, indicating the chronic nature of the synovitis. In the piglet model of ischemic osteonecrosis, hip synovitis, synovial effusion, and elevated IL-6 levels were also observed, indicating that hip synovitis and IL-6 elevation may be inherent to ischemic osteonecrosis. In-vitro experiments showed that hypoxic stress stimulates IL-6 release from articular chondrocytes which in turn produces inflammatory cytokine response from synovial cells.
*Conclusion:* LCPD produces significantly elevated IL-6 in the synovial fluid and chronic hip synovitis. Hypoxic stress on articular chondrocytes produced IL-6 elevation which in turn induced inflammatory cytokine response from synovial cells.
*Significance:* This is the first study to identify IL-6 as a specific inflammatory cytokine responsible for hip synovitis in LCPD. This line of research will lead to more effective treatment for synovitis in LCPD.

**April 18**
Scientific session: LCP; tumours + innovative
10:40–12:20
AUDITORIUM
**OP94**/11:12–11:18

## Long-term outcome following lower limb salvage with massive bone allograft and intramedullary free fibula (Capanna Technique) In paediatric and adolescent patients

Anthony Stans[1], Matthew Houdek[1], Eric Wagner[1], Franklin Sim[1], Steven Moran[1]

[1]Mayo Clinic, Rochester, United States

**LEVEL-IV**

*Purpose*: Following tumor resection, reconstruction of large segmental bone defects remains a major challenge in limb salvage surgery. Traditionally, structural allografts have been used to fill these voids. However, allograft reconstruction is associated with a high complication rate. The Capanna technique was developed to reduce complications and improve outcomes. It involves supplementing the massive allograft with an intramedullary vascularized free fibula. The purpose of this study is to examine intermediate term complications and outcomes using the Capanna technique for lower limb salvage in paediatric and adolescent patients.
*Methods*: Over a 15-year period 17 pediatric patients underwent lower extremity limb salvage using massive cadaveric allograft and intramedullary free fibular transfer (Capanna technique). Patients were followed prospectively and radiographic and medical records were reviewed for clinical and functional outcomes as well as postoperative complications. Time to union was determined by radiographic evaluation. Mankin functional outcome and Musculoskeletal Tumor Society (MSTS) rating scales were recorded for each patient.
*Results*: There were 8 male and 9 female patients, with an average age of 11(range 5–18) years at the time of surgery. Average follow-up was 6 (range 2–12) years. Limb salvage was successful in all patients. The average time to union of the allograft and fibula to the native bone was 10 months. Locked plating significantly reduced the time to union ($p = 0.03$). Six patients (35 %) underwent an additional procedure for symptomatic non-union. Sixteen (94 %) of the patients had a good or excellent Makin score, with an average MSTS rating of 94 at last follow-up. Six patients required a limb lengthening procedure. Two patients experienced wound infection and late allograft fracture occurred in 3 patients. Femoral reconstruction and male gender increased the odds of complications; however this was not statistically significant.
*Conclusion:* The Capanna technique is a reliable option for the reconstruction of large bone tumors of the lower extremity in paediatric patients. The use of locked plates improves union times. Limb preservation rates are excellent, and complication rates are acceptable.
*Significance*: The Capanna technique should be considered for paediatric and adolescent patients undergoing reconstruction of large, lower extremity, segmental bone defects.

**April 18**
Scientific session: LCP; tumours + innovative
10:40–12:20
AUDITORIUM
**OP95**/11:26–11:32

## Is “Softcast” (3 M) Strong Enough for Acute Potentially Unstable Paediatric Forearm Fractures?

Nimesh Patel[1], Lance Wilson[2], Guy Wansbrough[1]

[1]Torbay Hospital, Torquay, United Kingdom
[2]University of Technology, Queensland, Australia

**LEVEL-II**

*Introduction:* After orthopaedic assessment, the vast majority of paediatric forearm fractures are treated using a circumferential splint, with prior manipulation as necessary. Plaster of Paris is often chosen for its ease of application, cost and proven reliability. *Softcast* is an attractive alternative, as it provides a comfortable and water-resistant splint that can be removed without a plaster saw. Patient satisfaction has been demonstrated when used to protect buckle fractures, and its cost may be offset by reducing the number and duration of hospital visits. Unreinforced *Softcast* has not, however, been recommended for acute potentially unstable fractures.
*Objectives:* To establish whether a *Softcast* splint can provide sufficient mechanical stability to control a potentially unstable paediatric forearm fracture, and protect the wearer from further injury.
*Methods:* A laboratory study was undertaken to compare the bending, kinking and torsion loads withstood by standardised POP, *Softcast* and reinforced *Softcast* splints at clinically relevant endpoints.
*Results:* The load at clinical failure of a 6-wrap *Softcast* forearm splint was 504 N in bending, 202 N in kinking, and 11Nm in torsion (equalling 30.4, 26 and 42.2 % of the equivalent values for a circumferential 4-wrap POP). The 6-wrap *Softcast* was however stronger in all modes than a fibreglass-reinforced *Softcast* splint, such has been recommended for acute fractures. Furthermore, the load to failure in all modes exceeds that which can be exerted by body weight in many paediatric patients. *Softcast* demonstrated complete recovery of its original shape on unloading, where as POP was permanently deformed. POP splints were 4 % heavier than 6-wrap*Softcast*.
*Conclusion:* A 6-wrap *Softcast* splint provides adequate mechanical stability and protection for paediatric patients up to 20 kg, not engaged in high-risk activities. The primary risk is not of fracture angulation and loss of position, but temporary indentation of the splint against a point or edge, causing discomfort or pain. Considering its ease of removal, *Softcast* may be preferable for younger paediatric patients.

**April 18**
Scientific session: LCP; tumours + innovative
10:40–12:20
AUDITORIUM
**OP96**/11:32–11:38

## Two novel radiographic measurements for atlanto-axial instability in Down syndrome children

Naoyuki Nakamura[1]

[1]Kanagawa Child. Med. Center, Yokohama city, Japan

**LEVEL-IV**

*Introduction/background:* In pediatric Atlanto-axial Istability (AAI), early diagnosis is important for improved outcomes because of poor postoperative recovery in progressed neurological symptoms. Conventional dynamic radiography of the cervical spine is associated with a potential risk of worsening neurological symptoms. Therefore, we assessed the usefulness and reproducibility of two novel safe and simple radiographic measurements for atlantoaxial instability (AAI) in Down syndrome (DS) children.
*Materials and methods:* We retrospectively reviewed the medical records of 50 patients (24 boys and 26 girls) with AAI associated with DS. Of the 50 children, 11 had undergone and 4 had been scheduled for surgery (surgical group). In this investigation, in addition to the atlas-dens interval (ADI) and space available for spinal cord (SAC), we measured C1 inclination angle and C1/4 SAC ratio on lateral radiographs of the cervical spine in the neutral position. To assess the diagnostic abilities of these indices to determine indications for surgery, receiver operating characteristic (ROC) analysis of each index was performed. Their diagnostic abilities were compared using the area under the ROC curve (AUC). Moreover, we assessed reproducibility of our two proposed indices.
*Results:* The discriminatory abilities of C1/4 SAC (AUC, 1.00) and C1 inclination angle (0.91) were comparable with those of ADI (0.98) and SAC (0.95). For the interobserver and intraobserver reliability of the novel indices, the correlation coefficients ranged from 0.88 to 0.99. Correlation was observed between the ADI and C1/4 SAC ratio ($r = 0.507$, $p < 0.01$) and between the ADI and C1 inclination angle ($r = 0.407$, $p < 0.01$).
*Conclusion:* The diagnostic abilities of the two novel radiographic measurements were comparable with those of ADI and SAC. Moreover, these novel measurements can be obtained safely on lateral radiographs of the cervical spine in the neutral position.
*Significance:* Two novel measurements (C1 inclination angle, C1/4 SAC ratio) devised for AAI in DS children can be obtained safely on lateral radiographs of the cervical spine in the neutral position and indicated excellent ability of assessment of indication of surgery for AAI and high reproducibility.

**April 18**
Scientific session: LCP; tumours + innovative
10:40–12:20
AUDITORIUM
**OP97**/11:38–11:44

## A novel computer-assisted drill guide template for angular deformity

Qiang Shi[1], Xu Li[2]

[1]Academy of Orthopaedics, Guangzhou, China
[2]Academy of Orthopaedics, Guang, Guangzhou, China

**LEVEL-II**

*Introduction:* The present method of hemiepiphysiodesis for correction of angular deformity relies on anatomical landmarks for 8-plates placement. Placement of 8-plates using drill template has not been described in the literature. The authors reported on their experience with placement of 8-plates using a novel computer-assisted drill template in nine patients.
*Materials and methods:* 3D model of tibia was reconstructed by software MIMICS 10.01. The 3D tibial model was then exported in STL format, and opened in a workstation running software UG imageware12.0 for determining the optimal screw size and orientation. A virtual navigational template was established according to the tibial anatomic trait. The physical tibia and navigational template were manufactured using rapid prototyping. The navigational template was sterilized and used intraoperative to assist the placement of 8-plates.
*Results:* Nine 8-plates were placed and the accuracy of screw placement was confirmed with postoperative X-ray and CT scanning. There were not complications of related screws insertion. Average follow-up was 9 months (range 4–13 months), Postoperative computed tomographic scanning (CT) was available for allowing the evaluation of placement of 8-plates, all of which were in good position. The advantages of this technology over traditional techniques includes planning of the screw trajectory, which is done completely in the pre-surgical period as well as the ability to size the screw to the patient's anatomy.
*Conclusion:* This study shows a patient-specific template technique is easy to use, can simplify the operation and generates highly accurate screw placement.
*Significance:* The advantages of this technology over traditional techniques includes planning of the screw trajectory, which is done completely in the pre-surgical period as well as the ability to size the screw to the patient's anatomy.

**April 18**
Scientific session: LCP; tumours + innovative
10:40–12:20
AUDITORIUM
**OP98**/11:44–11:50

## Planovalgus foot deformity in hemiplegic children: a clinical and radiological evaluation after botulinum toxin injection into peroneus longus

Christophe Boulay[1], Michel Jacquemier[2], Elisabeth Castanier[2], Hadrien Giorgi[2], Guillaume Authier[2], Vincent Pomero[2], Brigitte Chabrol[2], Gérard Bollini[2], Jean-Luc Jouve[2], Elke Viehweger[2]

[1]CHU Timone Enfants, MARSEILLE, France
[2]CHU Timone Enfants, Marseille, France

**LEVEL-IV**

5. Miller [1] describes the natural history of planovalgus deformity in cerebral palsy (CP) as the most common foot deformity in all ages of CP children. He emphasizes that the planovalgus causes are especially an early muscle imbalance by overactivity of peroneal muscles and the primary pathology begins when the talus head is uncovered medially and inferiorly, associated with a talus equinus position on X-rays and a subluxated talar head causing a midfoot planus.
In hemiplegic children(<6 years) with planovalgus (in stance phase), a premature overactivity on EMG of Peroneus Longus (PL) during swing phase is described by Boulay [2]. with a dynamic equinus and hindfoot valgus at initial contact. This suggests that PL could be a therapeutic target by Botulinum Toxin (BoNT-A) injections. This hypothesis is tested in a retrospective study. The clinical and radiological efficiency is assessed.
Sixteen hemiplegic children GMFCS 1 or 2 (3.25 years ± 1.5) with a planovalgus foot deformity during the stance phase and, a dynamic equinus with hindfoot valgus and a premature overactivity of PL during the swing phase, were treated by a BoNT-A injection (Dysport®,Ipsen) into PL. Radiological foot parameters measured forefoot pronation, midfoot planus, valgus and equinus of hindfoot. These parameters are validated in the healthy and hemiplegic children. A paired T-test compared for each angle before and after toxin injection. The parameters described between pre-toxin vs normal data:calcaneal-pitch(8° vs17° $p < 0.001$), talo-calcaneal angle(55° vs 49° $p < 0.05$), lateral talo-first metatarsal angle(29° vs13°$p < 0.001$) and metatarsal-stacking angle(2°vs 8° $p < 0.001$). There was a non-significative difference, between pre vs post-toxin, for the calcaneal-pitch angle(7°vs9°) and the antero-posterior ankle angle(14° vs 15° $p < 0.05$). There was a significative difference, between pre vs post-toxin, for the talo-calcaneal angle(55°vs 46°$p < 0.001$), the lateral talo-first metatarsal angle(29° vs 18° $p < 0.01$) and the metatarsal stacking angle (2°vs 7° $p < 0.001$).
Before injections, the radiographs showed a hindfoot valgus, a dorsiflexed calcaneus (no equinus) with midfoot planus and a forefoot pronation. After injections, midfoot planus was reduced by a decrease of talus subluxation and there was not forefoot pronation. Injections into PL seemed to have a therapeutic actions on the forefoot and midfoot which clinically improved the hindfoot valgus but without action on the dorsalflexed of calcalenus depending on the gastrocnemius and soleus complex. PL could be an early therapeutic target for BoNT-A in planovalgus. Prospective studies will be needed to confirm these observations in the natural history of planovalgus.

[1] Miller F. Knee, leg and foot. In Cerebral palsy. Edited by Miller F. New York: Springer; 2005:667–802.
[2] Boulay C. Dynamic equinus with hindfoot valgus in children with hemiplegia.Gait Posture 2012, 36(1):108–112.

**April 16**
Scientific session: Foot & Ankle + Cerebral palsy
09:02 - 10:00
AUDITORIUM
**OP7** / 09:02 - 09:08

## Analysis of Serial Radiographs of the Foot to Determine Normative Values for the Growth of the First Metatarsal to Guide Hemiepiphyseodesis for Immature Hallux Valgus

Janelle Greene[1], Allen Nicholson[2], James Sanders[3], Daniel Cooperman[2], Raymond Liu[1]

[1]Case Western Reserve Univ, Cleveland, United States; [2]Yale University, New Haven, United States; [3]University of Rochester, Rochester, United States

**LEVEL-II**

**Purpose**: Hallux valgus deformity in the immature patient can be difficult to manage, as osteotomy can result in recurrence with additional growth. Lateral hemiepiphyseodesis of the first metatarsal offers a promising alternative, by permitting gradual correction of the intermetatarsal angle with growth. An important limitation of this approach is the lack of normative tables of first metatarsal growth to guide timing of intervention.

**Methods**: First-metatarsal lengths were measured from AP foot radiographs of children with an average of 9 serial radiographs from the historic Bolton Brush collection. For females, 95 patients totaling 894 x-rays were used ranging from 6 months to 18 years of age. For males, 120 patients totaling 1,003 x-rays were measured ranging from 8 months to 19.5 years of age. All patients with image series including a closed proximal metatarsal physis were sorted into the older group, with multipliers generated by setting last image to a multiplier of 1. Patients with serial imaging not inclusive of a closed physis were classified as the younger group, with multipliers based off of the multiplier at age 7 from the older group. First metatarsal multiplier values were then compared to published multiplier values for the overall foot.

**Results**: For both females and males, the multipliers followed a logarithmic curve versus age, with R-squared values of 0.922 and 0.894, respectively. Comparison of the first metatarsal multiplier values with previously studied multiplier values of the entire foot found small average differences of 0.053 in females and 0.034 in males, with R-squared values of 0.985 and 0.997.

**Conclusion**: The pattern of growth of the first metatarsal follows a logarithmic regression curve. These normative tables can be used to predict the amount of additional growth expected in the first metatarsal based on age and gender, and in turn predict the ideal age for hemiepiphyseodesis treatment.

**Significance**: Generation of normative multiplier tables of the first metatarsal allow for clinical prediction of first metatarsal remaining growth to guide timing of hemiepiphysiodesis for the surgical correction of hallux valgus deformity.

Open Access

This article is distributed under the terms of the Creative Commons Attribution License which permits any use, distribution, and reproduction in any medium, provided the original author(s) and the source are credited.

